# Sustained long-term disease correction in a murine model of MPSII following stem cell gene therapy

**DOI:** 10.1016/j.omtm.2023.101127

**Published:** 2023-10-20

**Authors:** Stuart Ellison, Aiyin Liao, Hélène F.E. Gleitz, Helen Parker, Laura Booth, John Robinson, Shaun Wood, Jessica Taylor, Rebecca Holley, Brian W. Bigger

**Affiliations:** 1 Stem Cell & Neurotherapies Group, University of Manchester, Manchester M13 9PT, UK

**Keywords:** Mucopolysaccharidosis, hematopoietic stem cell gene therapy, iduronate-2-sulfatase, Hunter disease, lentiviral vectors, MPSII, lysosomal storage disease

## Abstract

Mucopolysaccharidosis type II (MPSII) is a pediatric lysosomal storage disease caused by deficiencies in the IDS (iduronate-2-sulfatase) gene resulting in accumulation of glycosaminoglycans, multisystem disease, and profound neurodegeneration in severe forms. Although enzyme replacement therapy is available for somatic forms of disease, the inability of native IDS to pass the blood-brain barrier renders it ineffective for the brain. We previously demonstrated the short-term efficacy of a brain-targeted hematopoietic stem cell gene therapy approach to treat MPSII mice using lentiviral IDS fused to the blood-brain-barrier-crossing peptide ApoEII (IDS.ApoEII) in comparison with a lentivirus expressing native IDS and an unmanipulated bone marrow transplant. Here we evaluated the longevity of disease correction for 12–16 months following treatment. We observed sustained IDS enzyme activity in organs of long-term IDS.ApoEII-treated MPSII mice, similar to those analyzed 6 months post-treatment, with continued clearance of storage material in the brain and peripheral organs, maintained correction of astrogliosis, microgliosis, and correction of altered cytokines and chemokines. IDS.ApoEII also significantly reduced retinal atrophy, characteristic of MPSII. Overall, IDS.ApoEII resulted in systemic prevention of the MPSII phenotype, with no observed toxicity following treatment. This provides evidence of the sustained efficacy and safety of this treatment ahead of a recently opened clinical trial.

## Graphical abstract

**Figure:**
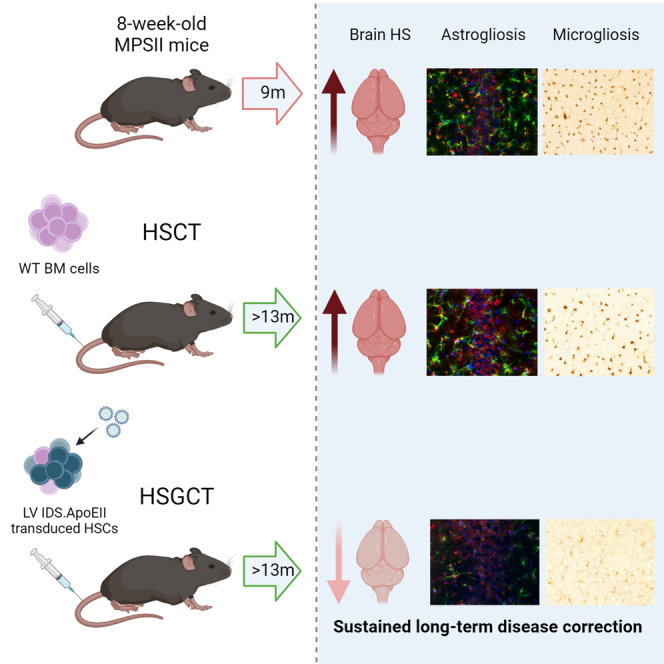

## Teaser

Bigger and colleagues developed a hematopoietic stem cell gene therapy for MPSII, a rare lysosomal storage disorder with significantly impaired quality and longevity of life. Long-term sustained efficacy and safety of the treatment were demonstrated in a murine disease model, ahead of a phase I/II clinical trial in MPSII patients.

## Introduction

Mucopolysaccharidosis type II (MPSII) is a rare pediatric lysosomal storage disorder (LSD) caused by iduronate-2-sulfatase (IDS) enzyme deficiency due to mutations in the *IDS* gene (GenBank: NM_000202). MPSII is X linked and therefore typically affects males, with a reported incidence of approximately 1.3 per 100,000 live births.^1^ The absence of functional IDS severely disrupts the normal degradation of both heparan sulfate (HS) and dermatan sulfate (DS), leading to their accumulation in all cells throughout the body. MPSII is a multisystemic disease with a range of characteristic symptoms, including skeletal abnormalities (dysostosis multiplex), joint stiffness and pain, short stature, cardiorespiratory disease, and hepatosplenomegaly.^2^^,^^3^ The most severe form of MPSII, characterized by progressive neurodegeneration and premature death, affects two-thirds of patients, with the majority not surviving into adulthood.^3^^,^^4^

Somatic symptoms in MPSII patients are generally well managed by intravenous delivery of replacement recombinant enzyme (Elaprase).^5^ Enzyme replacement therapy (ERT) is possible because circulating enzyme can enter affected cells via the M6P receptor and degrade HS and DS storage. Unfortunately, ERT is not effective at treating the CNS, as it is unable to cross the blood-brain barrier (BBB), although approaches are being investigated to remedy this.^6^^,^^7^ In addition, neutralizing antibodies and anaphylactic reactions are not uncommon following ERT, which can diminish the effectiveness of treatment.^8^

Historically, allogeneic hematopoietic stem cell transplantation (HSCT) has been the standard of care treatment of the similar LSD mucopolysaccharidosis type I (MPSI) (Hurler syndrome), with the ability to alleviate neurological symptoms and improve peripheral disease.^9^ However, although the treatment is usually successful, skeletal abnormalities, cardiac pathology, and occasionally CNS manifestations may be only partially improved after long-term follow-up.^10^ MPSII patients who undergo HSCT typically show poorer and more unpredictable clinical outcomes compared with MPSI patients, and the majority continue along the disease spectrum often with poor clinical correction, although a small percentage of patients do experience some improvements to the neurological indication.^11^

In a murine model of MPSII, we previously showed the efficacy of hematopoietic stem cell gene therapy (HSCGT) by using a lentiviral vector (LV)-based approach. This involved delivering a therapeutic transgene containing a codon-optimized IDS fused with a tandem repeat of the receptor binding domain of apolipoprotein E (ApoEII), which was driven by a myeloid-specific CD11b promoter in hematopoietic stem cells (HSCs).^12^^,^^13^ The rationale for inclusion of the ApoEII peptide was to further enhance CNS-targeted delivery of IDS enzyme from gene-modified cells in the periphery via receptor-mediated transcytosis across brain endothelial cells. The addition of the ApoEII peptide resulted in a more effective treatment (LV.IDS.ApoEII), completely normalizing brain pathology and behavior 6 months post-treatment, providing significantly enhanced correction compared with unmodified IDS (LV.IDS). Moreover, LV.IDS.ApoEII exhibited higher activity levels in the plasma and demonstrated a 5-fold increase in uptake in an *in vitro* model of brain endothelial cells. It was also found to undergo transcytosis through both ApoE-dependent receptors and M6P receptors.^12^

Here, we present findings from a long-term follow up study to determine whether efficacy and safety of HSCGT treatment is maintained throughout the lifetime of treated MPSII mice. The IDS and IDS.ApoEII treatment groups maintained supraphysiological levels of IDS activity in somatic organs, and IDS activity in the brain was sufficient to clear HS storage, correct sulfation patterns, and provide lasting reductions in astrogliosis, lysosome swelling, and microgliosis. Histological analysis of tissues from animals treated with LV.IDS and LV.IDS.ApoEII confirmed sustained efficacy beyond 12 months post-treatment, with no indication of toxicity except for effects associated with busulfan. Furthermore, both LV.IDS and LV.IDS.ApoEII treatments were able to rectify altered chemokine and cytokine profiles in MPSII, with LV.IDS.ApoEII demonstrating superior overall efficacy. These findings provide additional safety and efficacy data to support our recently opened phase I/II clinical trial in MPSII patients (NCT05665166).

## Results

### MPSII HSCGT study design to assess long-term efficacy and safety

**Figure 1:**
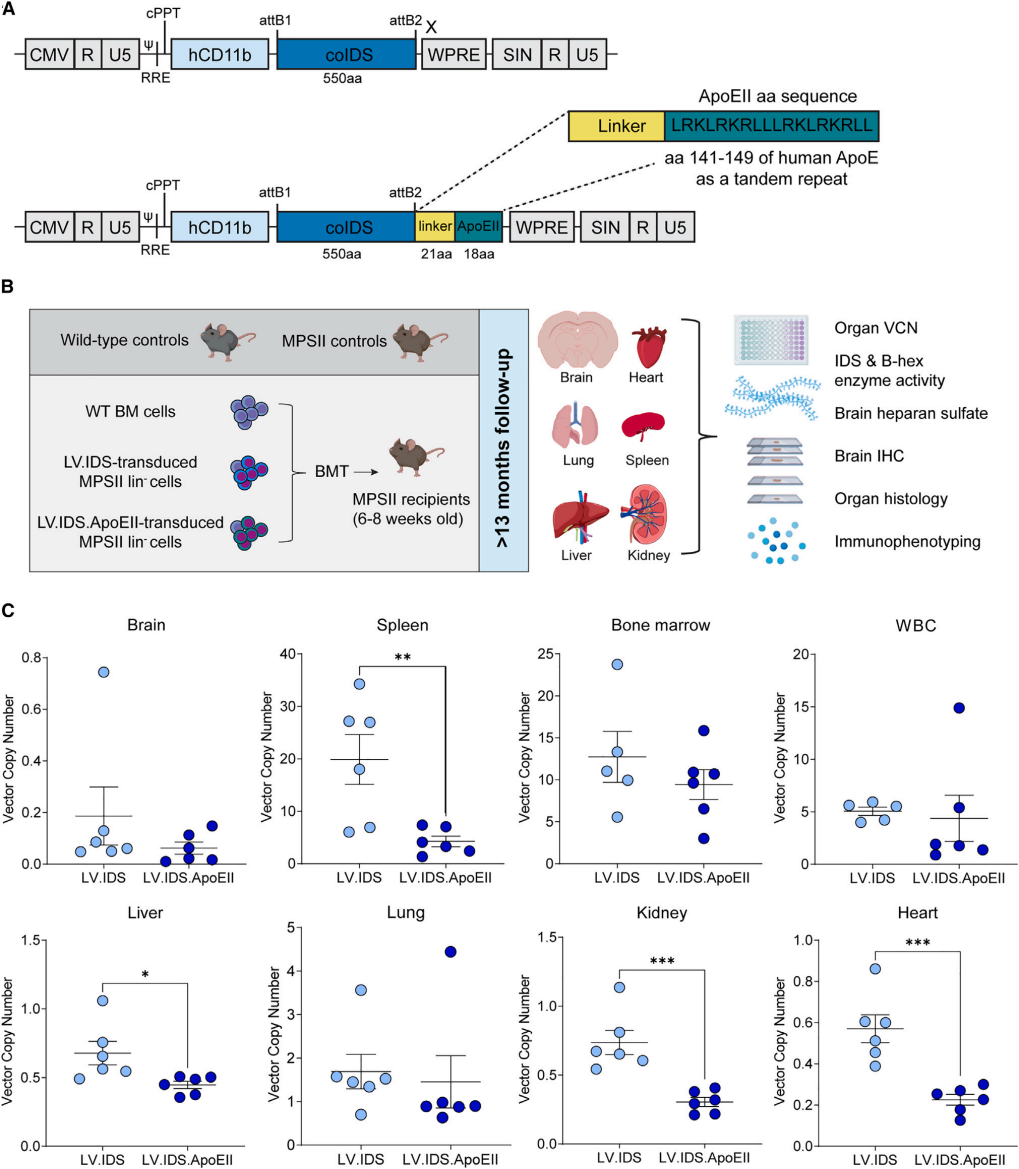
Lentiviral vector design and long-term study overview (A) pCCL lentiviral vectors containing the CD11b promoter encoding codon-optimized human IDS gene or the human IDS gene followed by a flexible linker and the ApoEII peptide sequence tandem repeat. (B) The long-term HSCGT strategy. Busulfan-conditioned 6- to 8-week-old MPSII mice were transplanted with 2–3 × 10^5^ lineage-depleted (Lin^−^) HSCs transduced with LV.IDS or LV.IDS.ApoEII or 1 × 10^7^ total bone marrow cells for the WT-HSCT group. Untreated WT and MPSII mice were included as controls. Six animals per group were sacrificed at ≥13 months of age for biochemical and microscopic analysis including qPCR for vector copy number (VCN), IDS enzyme activity, β-hex activity, brain heparan sulfate, brain immunohistochemistry, immunophenotyping, and histology of organs. (C) VCN analysis in brain, spleen, bone marrow, liver, heart, lung, kidney, and white blood cells (WBCs). Data are mean ± SEM. Student's t-test: ∗∗p < 0.01, ∗∗∗p < 0.001.

The aim of this study was to assess the long-term effectiveness of *ex vivo* HSCGT as a treatment for MPSII. We previously designed 2 LVs to express either IDS alone or IDS fused to the BBB-targeting ApoEII peptide (Figure 1A).^12^ Lineage-depleted MPSII HSCs were transduced with either LV.IDS or LV.IDS.ApoEII and transplanted into 16 busulfan-conditioned 6- to 8-week-old MPSII recipient mice (Figure 1B). As a control, wild-type (WT) HSCs were transplanted into MPSII mice to mimic an allogeneic bone marrow (BM) transplant (WT-HSCT). These cohorts and the methodology for transplantation were identical to our short-term study analyzed at 8 months of age and were performed at the same time.^12^ These mice formed half of the behavioral cohort analyzed, and fully presented, by Gleitz et al.^12^ Mice were culled at their humane endpoint between 14 and 18 months of age, via perfusion with PBS, and 9 organs taken for subsequent analysis.

The number of integrated vector copies (vector copy number [VCN]) was assessed in the brain, spleen, BM, liver, heart, lung, kidney, and white blood cells [WBCs] of LV.IDS- and LV.IDS.ApoEII-treated mice (Figure 1C). Overall, VCN levels were higher in the LV.IDS-treated animals compared with LV.IDS.ApoEII, with a significantly higher VCN in the spleen (19.9 vs. 4.3), liver (0.68 vs. 0.45), heart (0.57 vs. 0.22), and kidney (0.74 and 0.3) and a non-significant trend of higher VCN in the brain (0.19 vs. 0.06), BM (12.72 vs. 9.43), lung (1.69 vs. 1.45), and WBCs (5.05 vs. 4.38) between the IDS and IDS.ApoEII groups, respectively. Lower VCNs in the LV.IDS.ApoEII group could be a consequence of either engraftment of HSCs with lower transduction after transplantation, as there was a very wide spread of pre-transplantation transduction efficiency of IDS.ApoEII seen in Figure 1 in Gleitz et al.,^12^ or possibly lower engraftment potential in peripheral organs by IDS.ApoEII.

### Supraphysiological IDS enzyme activity levels are sustained in long-term LV.IDS and LV.IDS.ApoEII mice

**Figure 2:**
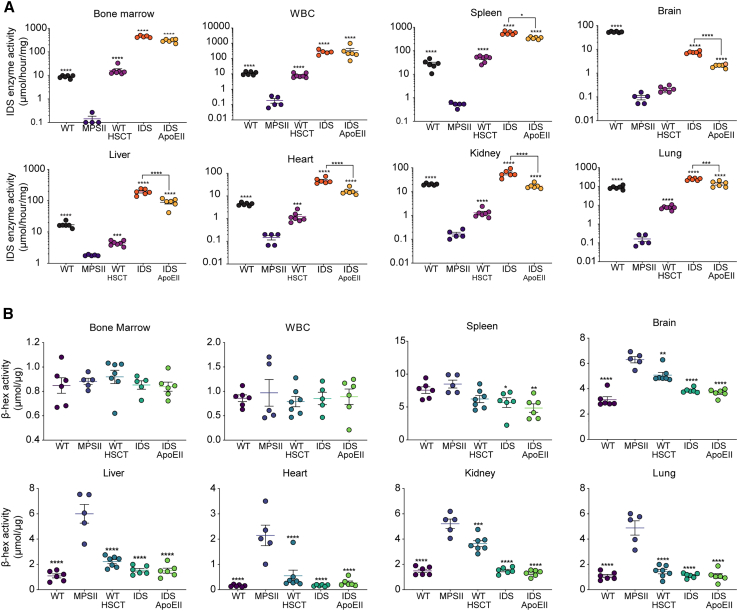
LV.IDS and LV.IDS.ApoEII provide sustained brain-specific IDS activity and supra-physiological levels of active IDS in peripheral organs (A) IDS enzyme activity levels measured in organs taken at ≤13 months of age, including brain, spleen, bone marrow, liver, heart, lung, kidney, and WBCs from control and treated mice (n = 6–10 mice/group). Black dots, WT; purple dots, MPSII; pink dots, WT-HSCT; orange dots, IDS; yellow dots, IDS ApoEII. (B) Levels of lysosomal enzyme β-hexosaminidase activity in brain, spleen, bone marrow, liver, heart, lung, kidney, and WBCs of ≤13-month-old mice (n = 6–10 mice/group). Purple dots, WT; blue dots, MPSII; turquoise dots, WT-HSCT; green dots, IDS; light green dots, IDS ApoEII. All data are mean ± SEM. One-way ANOVA: ns, p > 0.05; ∗p < 0.05, ∗∗p < 0.01, ∗∗∗p < 0.001, and ∗∗∗∗p < 0.0001 vs. MPSII; other comparisons are indicated by brackets. For IDS enzyme activity, log-transformed data were used for statistical analysis.

We measured iduronate sulfatase and β-hexosaminidase (β-hex) activity levels in long-term-treated MPSII mice (Figure 2) and observed supraphysiological levels of IDS activity in most organs from the IDS and IDS.ApoEII treatment groups (Figure 2A), with particularly high levels recorded in the spleen (1,984% and 1,244% of WT levels, respectively), and hematopoietic system (BM, 5,068% and 1,006%; WBCs, 2,450% and 2,930%). Very high IDS activity was also detected in the liver (1,134% and 524% of WT), heart (1,100% and 370%), and lung (277% and 156%) in the IDS and IDS.ApoEII transplantation groups, respectively (Figure 2A). In the kidney, the IDS treatment group provided approximately 3.5-fold WT IDS levels where the IDS.ApoEII treatment IDS activity was comparable with WT. In the brain, we observed 14% and 4% of WT IDS activity levels in the IDS and IDS.ApoEII treatment groups, respectively (Figure 2A). Overall higher levels of IDS activity were recorded in the IDS only treatment group, in line with the VCN data (Figure 1C). Elevated endogenous lysosomal enzyme β-hex levels are typical of MPS disorders and believed to rise as a consequence of lysosomal enzyme deficiency.^14^ As expected, we observed significantly elevated β-hex levels in the brain, liver, heart, lung, and kidney of untreated MPSII mice (Figure 2B). Both IDS and IDS.ApoEII treatments were able to correct β-hex activity to WT levels in the liver, heart, lung, and kidney more effectively than the WT-HSCT cohort. In the brain, both IDS and IDS.ApoEII treatments significantly reduced β-hex activity in MPSII mice by 38.6% and 41.6%, respectively, improving upon the 19.3% reduction seen following WT-HSCT.

### HS storage in brain and liver is corrected with LV.IDS and LV.IDS.ApoEII

**Figure 3:**
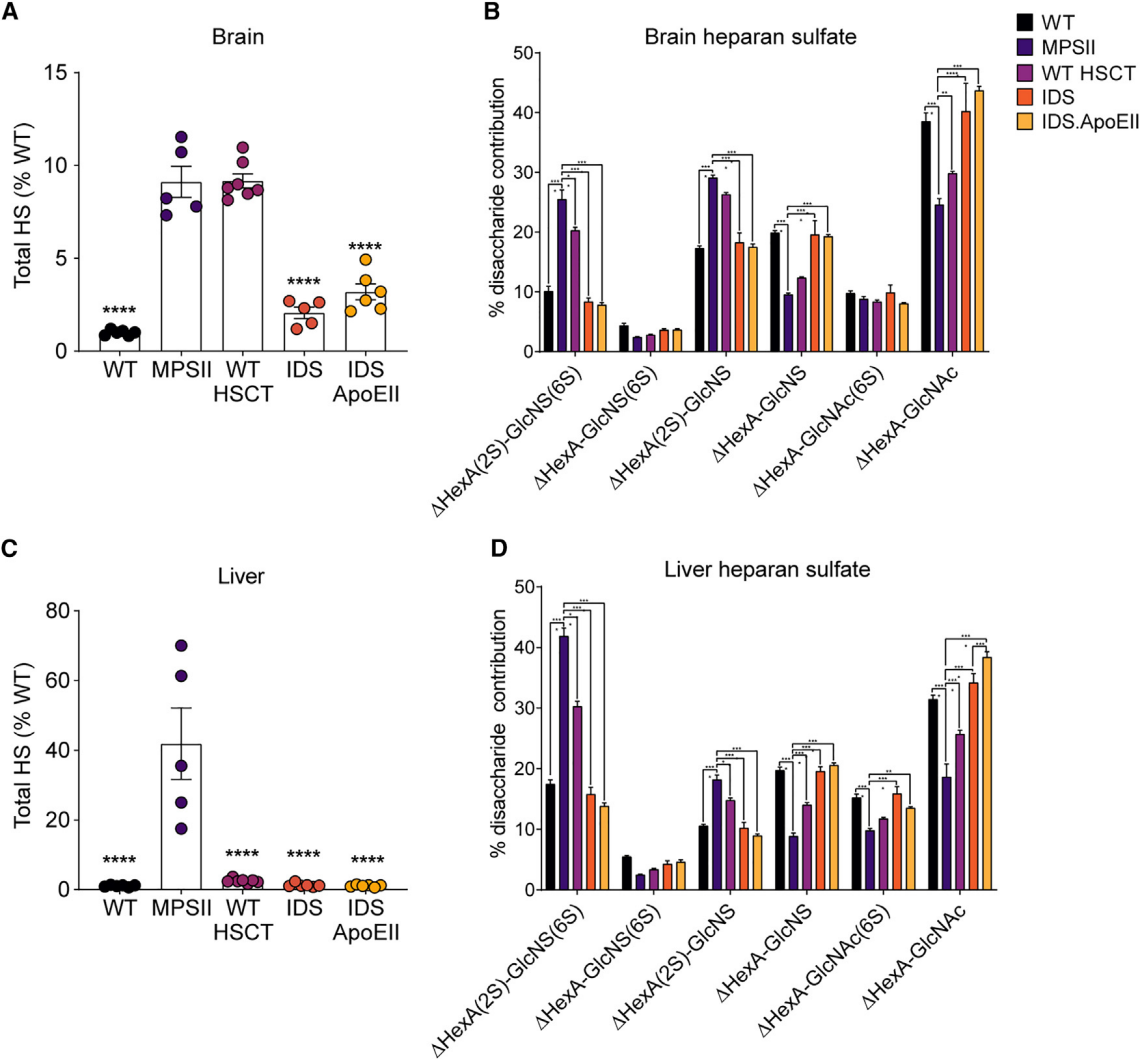
LV.IDS and LV.IDS.ApoEII significantly reduce brain HS accumulation and normalize HS sulfation patterning Total relative amounts of HS and compositional disaccharide analysis of HS from control and treated mice brain (A and B) and liver (C and D) samples (n = 6–10 mice/group). Black, WT; purple, MPSII; pink, WT-HSCT; orange, IDS; yellow, IDS ApoEII group. All data are mean ± SEM. One-way ANOVA: ns, p > 0.05; ∗p < 0.05, ∗∗p < 0.01, ∗∗∗p < 0.001, and ∗∗∗∗p <0.0001 vs MPSII.

HS accumulation in the brain is believed to be a major contributing factor to neurocognitive decline in MPS patients.^15^ In addition, the altered sulfation patterning is also a trigger for neuroinflammatory events that accelerate disease progression.^16^ Both IDS and IDS.ApoEII were able to significantly reduce MPSII brain HS levels by 4.3- and 2.8-fold, respectively, and normalize the HS sulfation patterning in comparison with WT-HSCT, where total HS levels were not reduced in treated MPSII mice, and only a partial change in HS sulfation patterning was observed (Figures 3A and 3B). In the liver, all treatment groups were able to restore HS to WT levels, but only IDS and IDS.ApoEII treatments were able to normalize HS sulfation patterning (Figures 3C and 3D).

### Sustained reductions in astrogliosis, lysosome swelling, and microgliosis in LV.IDS- and LV.IDS.ApoEII-treated MPSII mice

**Figure 4:**
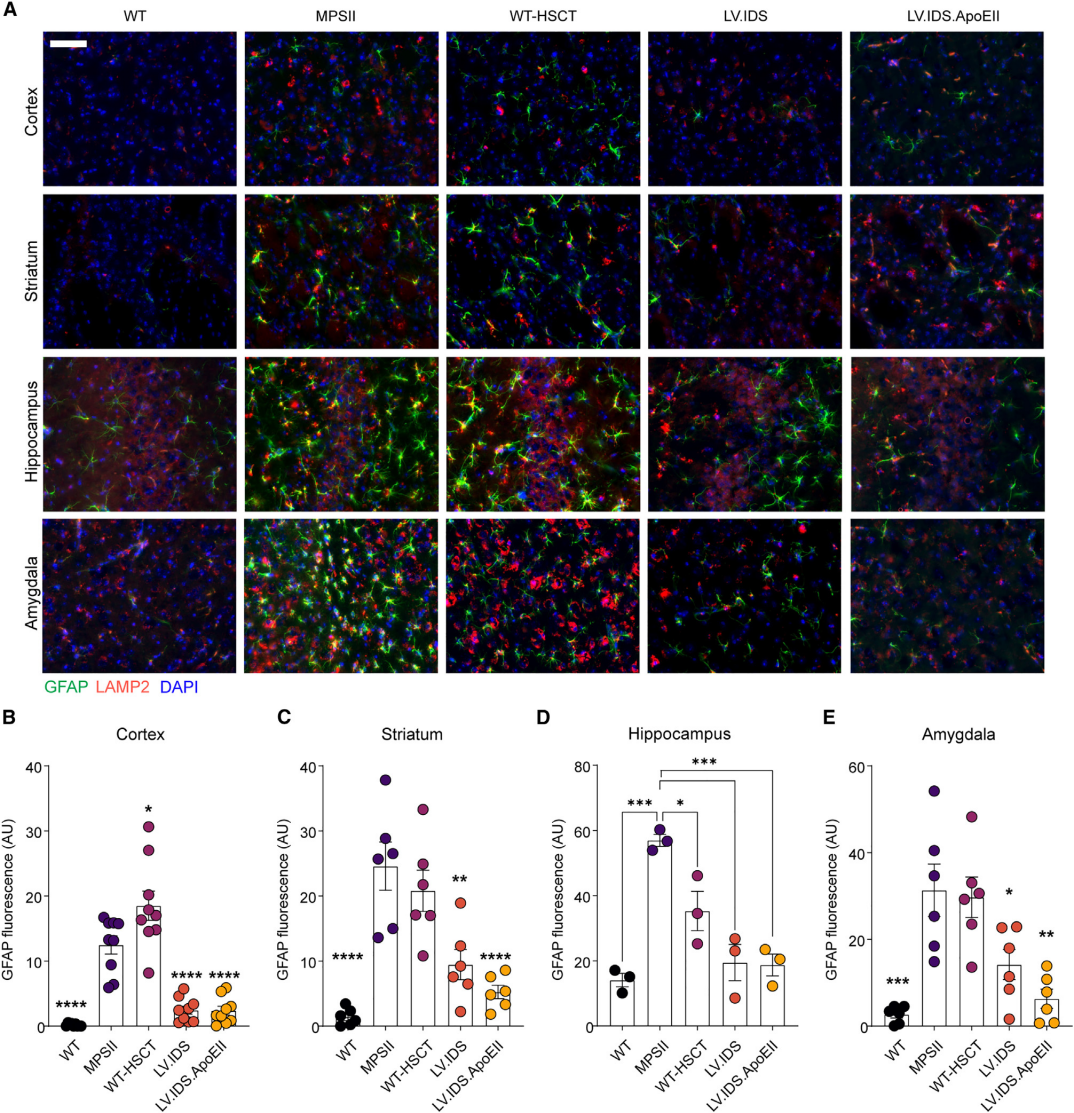
LV.IDS.ApoEII normalizes astrogliosis in the brains of ≤13-month-old MPSII mice (A) Representative images of 30 μm brain sections of the motor cortex (M2), striatum (both −0.46 mm from bregma), hippocampus (CA3), and amygdala (both −1.22 mm from bregma) from control and treated mice stained with GFAP (green) and LAMP2 (red) (n = 6 mice/group), 40×; nonlinear adjustments were made equally to reduce background: gamma 0.72 and input levels of 0–190. Scale bar: 50 μm. GFAP immunofluorescence was quantified in the cortex (B), striatum (C), hippocampus (D), and amygdala (E) of ≤13-month-old MPSII mice (n = 6 mice/group). Black dots, WT; purple dots, MPSII; pink dots, WT-HSCT; orange dots, IDS; yellow dots, IDS ApoEII. AU, arbitrary units. Data are mean ± SEM. One-way ANOVA: ∗p < 0.05, ∗∗p <0.01, ∗∗∗p < 0.001, and ∗∗∗∗p < 0.0001 vs. MPSII.

**Figure 5:**
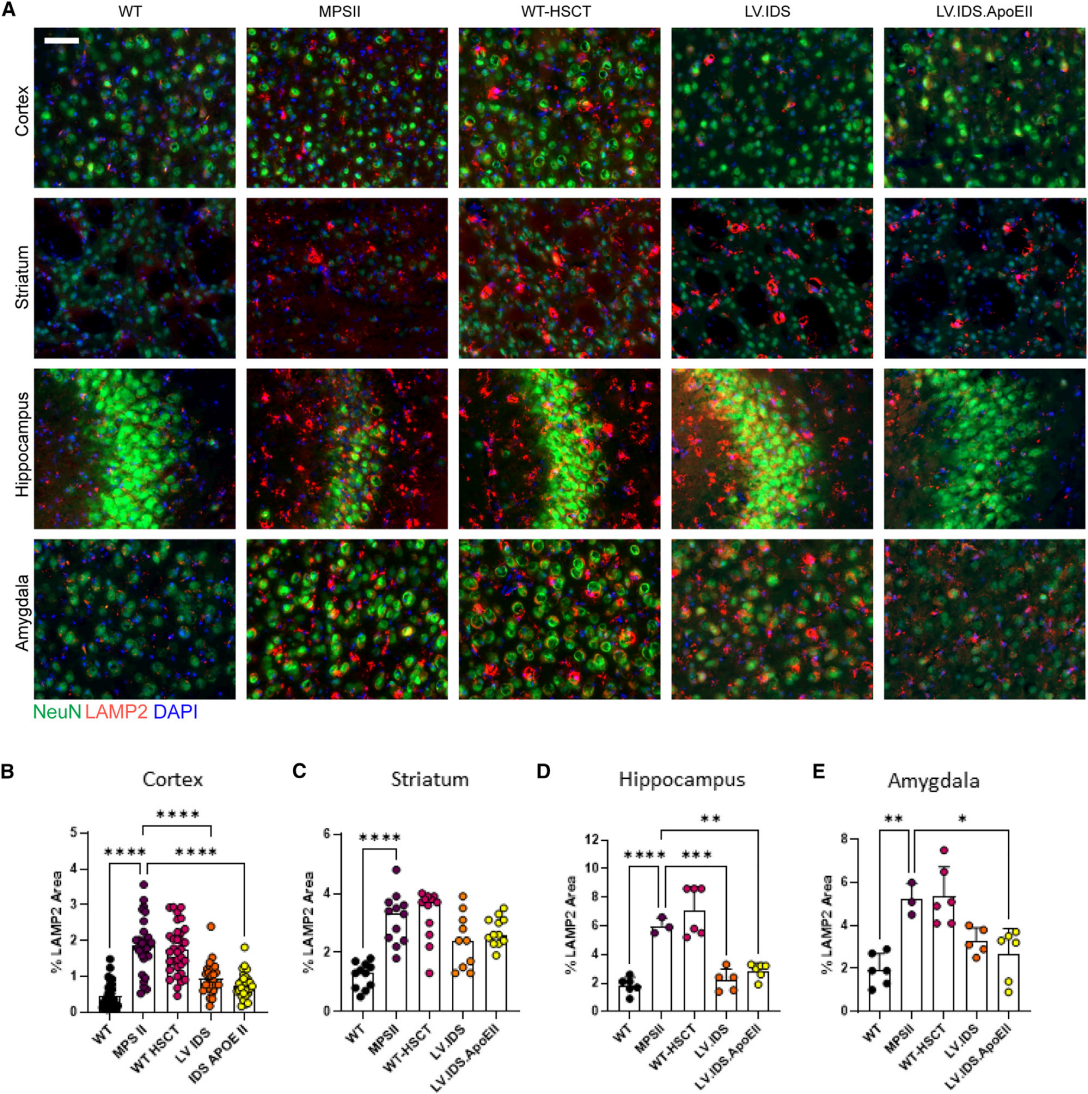
LV.IDS.ApoEII reduces lysosomal swelling and neuronal cell shrinkage in the brains of ≤13-month-old MPSII mice (A) Representative images of 30 μm brain sections of the motor cortex (M2), striatum (both −0.46 mm from bregma), hippocampus (CA3), and amygdala (both −1.22 mm from bregma) from control and treated mice stained with NeuN (green) and LAMP2 (red) (n = 6–10 mice/group). Quantification of percentage area of LAMP2 staining in the cortex (B), striatum (C), hippocampus (D), and amygdala (E). Black dots, WT; purple dots, MPSII; pink dots, WT-HSCT; orange dots, IDS; yellow dots, IDS ApoEII. Data are mean ± SEM. One-way ANOVA: ∗p < 0.05, ∗∗p <0.01, ∗∗∗p < 0.001, and ∗∗∗∗p < 0.0001 vs. MPSII.

**Figure 6:**
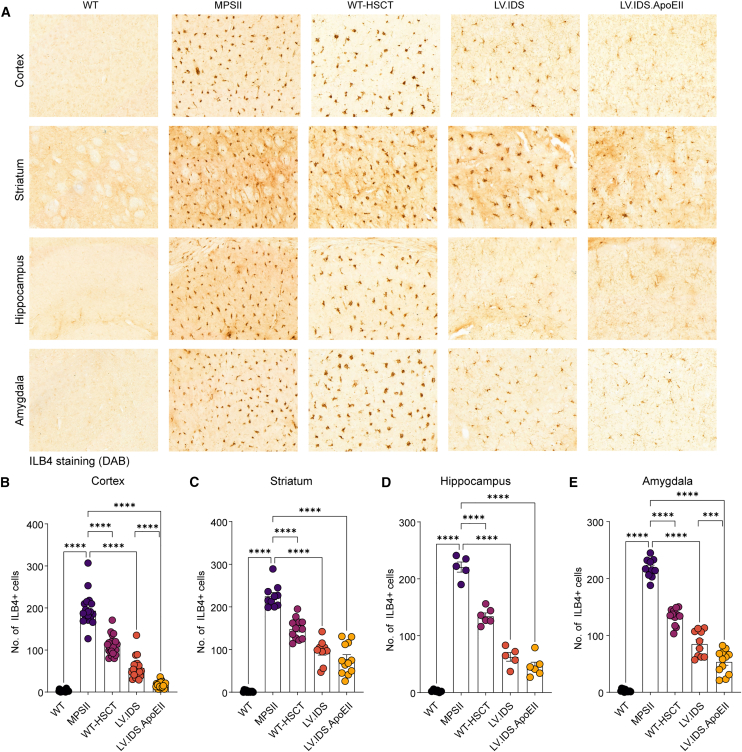
LV.IDS.ApoEII normalizes microgliosis in the brains of ≤13-month-old MPSII mice (A) Representative images of 30 μm brain sections of the motor cortex (M2), striatum (both −0.46 mm from bregma), hippocampus (CA3), and amygdala (both −1.22 mm from bregma) from control and treated mice stained with isolectin B4 (ILB4) to identify activated microglia, 40×. Scale bar: 50 μm. (B) Four 30 μm sections per mouse of the cortex (B), striatum (C), hippocampus (D), and amygdala (E) were counted for the number of ILB4-positive cells (0.26 to −1.94 mm from bregma), n = 6–10 mice/group. Black dots, WT; purple dots, MPSII; pink dots, WT-HSCT; orange dots, IDS; yellow dots, IDS ApoEII. Data are mean ± SEM. One-way ANOVA: ∗p < 0.05, ∗∗p <0.01, ∗∗∗p < 0.001, and ∗∗∗∗p < 0.0001 vs. MPSII.

Astrogliosis and lysosomal swelling are hallmark features of MPSII and can be observed throughout various regions of the brain, most notably in the cortex, striatum, hippocampus, and amygdala (Figure 4A). Immunohistochemical staining using a glial fibrillary associated protein (GFAP) stain for reactive astrocytes (green) demonstrated significantly increased astrogliosis in untreated MPSII mice compared with WT in all brain regions tested. In the cortex and hippocampus, LV.IDS and LV.IDS.ApoEII significantly reduced MPSII associated astrogliosis by similar levels (Figures 4A, 4B, and 4D). In the striatum and amygdala, LV.IDS.ApoEII was the most effective treatment for astrogliosis reduction and was more significantly reduced than LV.IDS with almost complete normalization (Figures 4C and 4E). Co-staining of the same coronal sections with the lysosome marker LAMP2, and sections co-stained with the neuronal markers NeuN and LAMP2, indicated a substantial reduction of swollen lysosomes in the IDS and IDS.ApoEII treatment groups to near normal levels in both astrocytes (Figure 4A) and neurons (Figure 5) in the cortex and hippocampus, but only for IDS.ApoEII in the amygdala, compared with the WT-HSCT group, in which there appeared to be little to no correction of lysosomal swelling. Interestingly, lysosomal swelling in the striatum was less effectively corrected with both IDS and IDS.ApoEII treatment groups compared with other brain regions (Figures 5A and 5C). In untreated MPSII mice, we observed increases of 67-, 118-, 82.6-, and 79.3-fold of isolectin B4 (ILB4)-positive activated microglial cells in the cortex, striatum, hippocampus, and amygdala, respectively (Figure 6). In the four brain regions evaluated, IDS.ApoEII was the most effective at normalizing the number of activated microglia with significant reduction observed in the cortex and amygdala compared with LV.IDS (Figures 6B and 6C), and this was also the most effective treatment at reducing lysosomal storage in neurons (Figure 5A). Overall, these findings suggest that the characteristic astrogliosis and microglial activation in the brains of MPSII mice can be prevented over a sustained period following treatment with LV.IDS and LV.IDS.ApoEII, with LV.IDS.ApoEII being the most effective, despite four times lower IDS enzyme activity levels than LV.IDS.

### LV.IDS.ApoEII treatment corrects MPSII associated eye abnormalities

**Figure 7:**
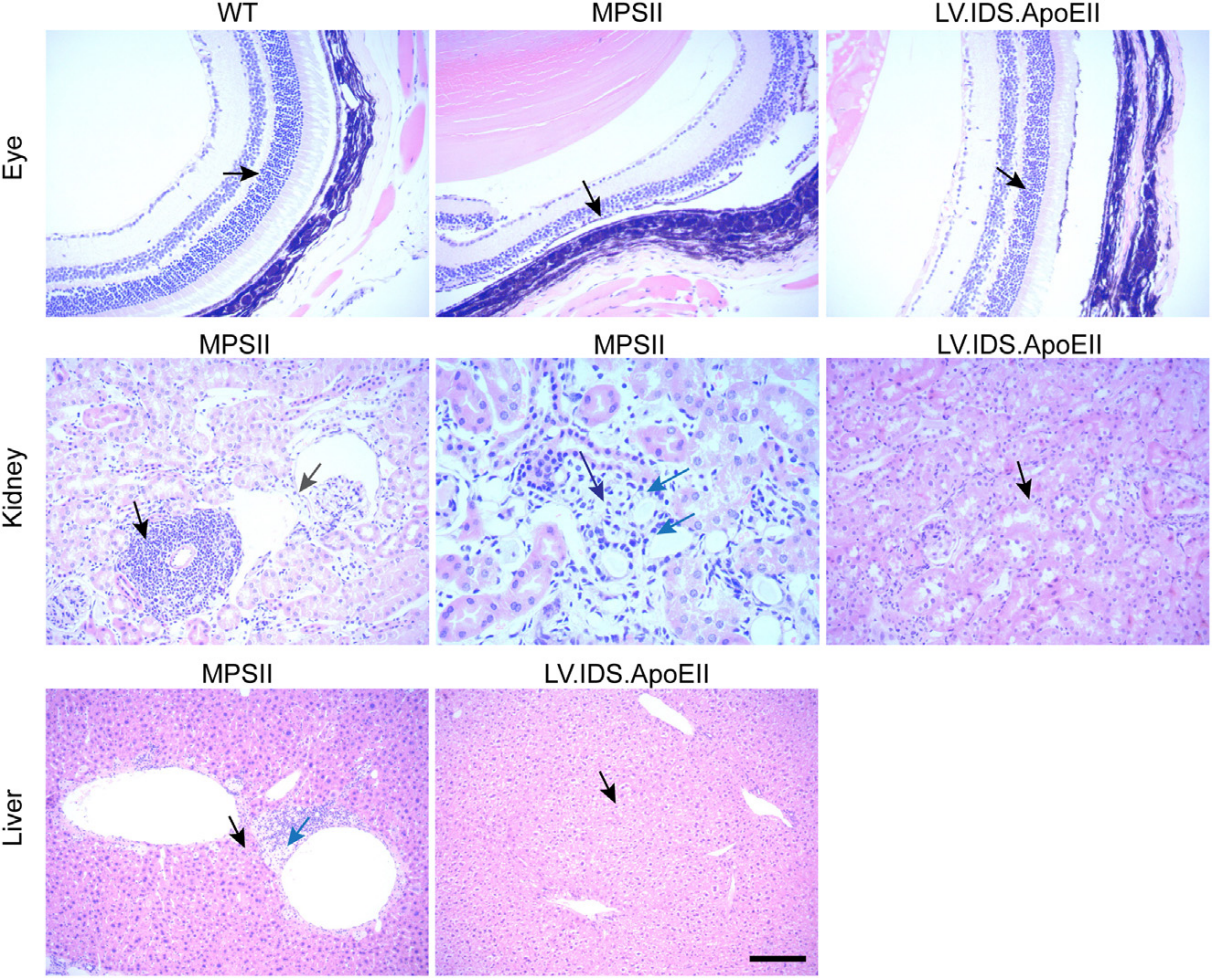
H&E staining of the eye, kidney, and liver to evaluate efficacious effect of treatment upon the MPSII phenotype and potential toxicological effects Sections were analyzed from control and treatment groups by light microscopy and initially blinded (n = 7 mice/group). For the eye, black arrows highlight the near total loss of outer nuclear rods and cones observed in MPSII mice compared with the minimal loss observed in IDS.ApoEII-treated mice, similar to WT. In MPSII kidneys, the black arrow indicates regions of peri-vascular inflammation, the gray arrow highlights peri-glomerular vacuolation, the dark blue arrow indicates vacuolated macrophages with the interstitium, and the light blue arrows highlight vacuolated tubular epithelium. Minimal tubular vacuolation is observed in the kidneys of IDS.ApoEII-treated MPSII mice (black arrow). In MPSII liver, regions of peri-vascular inflammation (black arrow) and expansion of peri-venous space by vacuolated macrophages (blue arrow) are indicated. In livers from IDS.ApoEII-treated MPSII mice, minimal hepatocellular vacuolation was observed.

Within the scope of pre-clinical safety and efficacy investigations, fixed tissue samples from each experimental group were submitted to an external organization, Histologix, to conduct H&E evaluations under good clinical laboratory practice (GCLP)-like conditions, to identify any potential organ toxicity alongside biodistribution. These studies were designed to (1) assess the efficacy of treatment on the MPSII phenotype; (2) evaluate any toxicological effects following WT-HSCT, LV.IDS, or LV.IDS.ApoEII HSCGT; and (3) assess any toxicological effects of busulfan co-administration. As expected, findings associated with the intra-cellular accumulation of GAG (cytoplasmic vacuolation), along with secondary atrophic, inflammatory, and degenerative findings were most pronounced in the MPSII control group (Figure S1). There was an attenuation of these findings with WT-HSCT administration, most notably in the eye, heart, kidney, liver, and spleen (Figure S1). This effect was mirrored with both LV.IDS and LV.IDS.ApoEII administration, and therefore indicative of favorable efficacy. Of particular note was the profound reduction in the severity of retinal atrophy among the LV.IDS.ApoEII treatment group (Figure 7). Similarly, in the kidney and liver, LV.IDS.ApoEII treatment resulted in minimal tubular vacuolation and minimal hepatocellular vacuolation compared with untreated animals (Figure 7). Overall, the administration of LV.IDS or LV.IDS.ApoEII offered multisystem efficacy in the attenuation of the severity of the MPSII phenotype. There was no indication of toxicity following treatment using either vector, apart from some expected toxicological effects observed as a result of busulfan co-administration, especially in kidney and lung (Figure S1). No neoplasms or lymphomas or evidence of malignancy were reported in treated or untreated mice.

### LV.IDS.ApoEII treatment reverses the altered cytokine and chemokine profiles of MPSII mice

**Figure 8:**
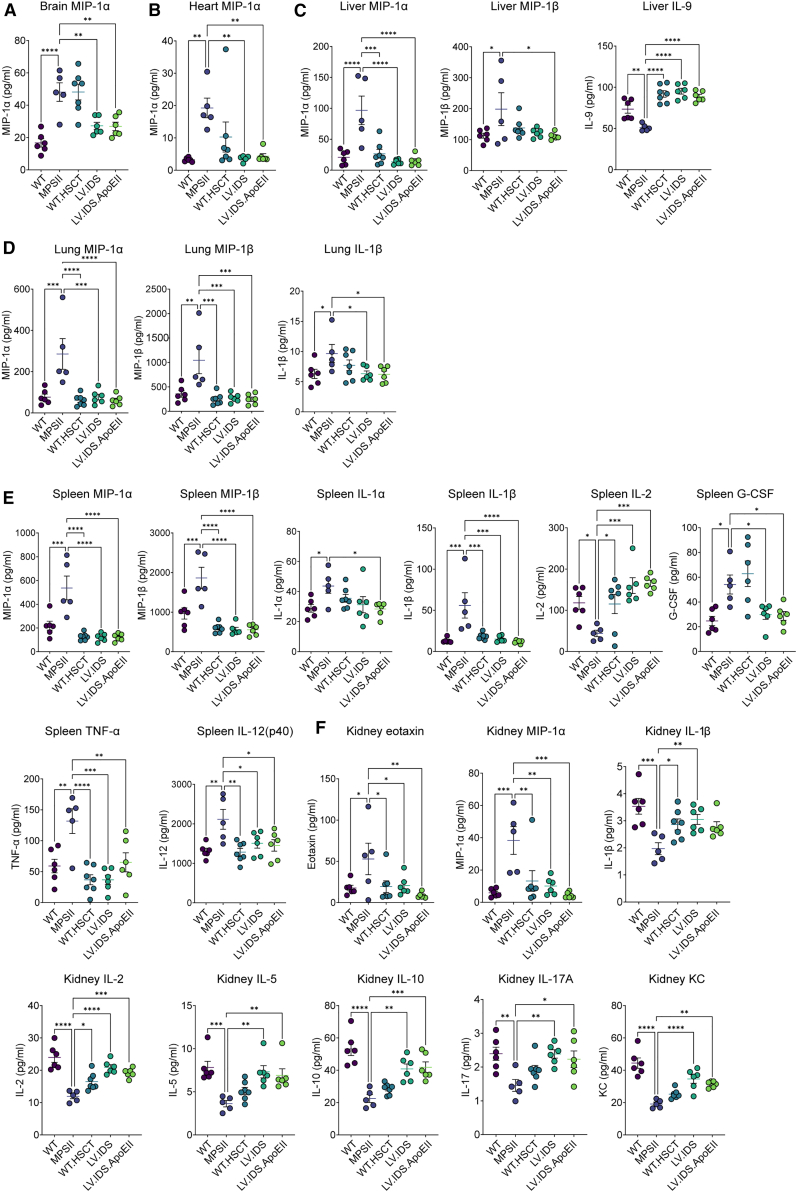
LV.IDS.ApoEII corrects altered cytokine and chemokine profiles in MPSII mice Brain (A), heart (B), liver (C), lung (D), spleen (E), and kidney (F) samples were evaluated in a 23-plex Bio-Plex assay. Only cytokines/chemokines profiles with significant differences between MPSII and WT are shown. Purple dots, WT; blue dots, MPSII; turquoise dots, WT-HSCT; green dots, LV.IDS; light green dots, LV.IDS.ApoEII. Data are mean ± SEM. One-way ANOVA: ns, p > 0.05; ∗p < 0.05, ∗∗p < 0.01, ∗∗∗p < 0.001, and ∗∗∗∗p < 0.0001 vs. MPSII.

Among the numerous clinical manifestations described for MPS, chronic immune dysregulation has been consistently reported.^16^^,^^17^^,^^18^ The molecular mechanisms involved in the inflammatory response however are still not fully understood, but inflammasome activation has been described in MPSII, which is usually associated with elevated IL-1β. We used a Bio-Plex multiplex immunoassay system to identify significantly dysregulated cytokine and chemokine levels in MPSII mice compared with WT mice. Furthermore, we assessed whether our WT-HSCT or HSCGT treatments could restore any observed alterations back to WT levels. Additionally, we investigated whether the inclusion of the ApoEII tag in LV.IDS.ApoEII treatment led to an unfavorable cytokine response compared with LV.IDS treatment. A 23-plex assay was used to profile the following cytokines and immune mediators: IL-1α, IL-1β, IL-2, IL-3, IL-4, IL-5, IL-6, IL-9, IL-10, IL-12 (p40), IL-12 (p70), IL-13, IL-17A, eotaxin, G-CSF, GM-CSF, IFN-γ, KC, MCP-1, MIP-1α, MIP-1β, RANTES, and TNF-α. Cytokine profiles in the brain, heart, liver, lung, spleen, and kidney were evaluated in control and treated groups (Figure 8). Only cytokines or chemokines where a significant difference was observed between MPSII and WT are presented. In the brain, MIP-1α levels were elevated in MPSII mice compared with WT. Both LV.IDS and LV.IDS.ApoEII treatments were able to significantly reduce MIP-1α levels in the brain, whereas the WT-HSCT did not (Figure 8A). MIP-1α was also elevated in the MPSII heart, which was normalized back to WT levels in all treatment groups, including WT-HSCT. In the liver, MIP-1α and MIP-1β were significantly elevated in MPSII and IL-9 levels reduced (Figure 8C). All treatment groups were able to reduce MIP-1α levels, whereas only IDS.ApoEII was able to normalize MIP-1β. All 3 treatment groups were able to significantly improve IL-9 levels above MPSII (Figure 8C). In the lung, MIP-1α, MIP-1β, and IL-1β were elevated in MPSII. MIP-1α and MIP-1β were normalized in all treatment groups, while IL-1β levels were normalized with IDS and IDS.ApoEII treatment (Figure 8D). In the spleen and kidney, there were significant changes in 8 different cytokines and chemokines identified in MPSII mice compared with WT (Figures 8E and 8F). In the spleen, the elevated levels of MPI-1α, MIP-1α, IL-1β, TNF-α, and IL-12 (p40) observed in MPSII were all corrected in WT-HSCT, IDS, and IDS.ApoEII treatment groups (Figure 8E). However, only IDS.ApoEII treatment significantly reduced IL-1α levels. IL-2 levels were significantly reduced in MPSII spleens compared with WT, and all 3 treatment groups were able to reverse this. G-CSF levels were elevated in the MPSII group, which were significantly reduced following treatment with IDS and IDS.ApoEII but not WT-HSCT. Interestingly, in the kidney, only eotaxin and MIP-1α saw significantly elevated levels in MPSII mice compared with significantly reduced levels of IL-1β, IL-2, IL-5, IL-10, IL-17A, and KC (Figure 8F). The elevated levels of eotaxin and MIP-1α and the reduced level of IL-2 were normalized in all 3 treatment groups. The decreased levels of IL-5, IL-10, IL-17A, and KC were significantly increased by both IDS and IDS.ApoEII but not by WT-HSCT. The decreased level of IL-1β was significantly increased by WT-HSCT and IDS treatment and elevated but not statistically significant in the IDS.ApoEII treatment group. Overall, IDS and IDS.ApoEII were similarly effective at normalizing altered cytokine and chemokine levels observed in MPSII mice. Notably, cytokine responses between LV.IDS and LV.IDS.ApoEII were consistently similar to each other, suggesting no toxic effects from the ApoEII tag.

## Discussion

The purpose of this study was to determine if efficacy and safety of HSCGT treatment with LV.IDS.ApoEII can be maintained over the lifetime of treated MPSII mice. In our initial proof-of-concept (PoC) investigation, which provided efficacy data 6 months post-treatment, we observed significant improvements in working memory deficits, neuro-inflammation, and HS storage in the brain of MPSII mice treated with LV.IDS.ApoEII. Additionally, we observed normalized rotarod activity, reduced peripheral inflammation, and improvements in other somatic disease markers associated with MPSII.^12^ These improvements were only partially achieved with the unmodified LV.IDS vector, suggesting that LV.IDS.ApoEII offers superior correction capabilities. For the long-term study, the mice included in this analysis constituted half of the behavioral cohort previously reported in Gleitz et al.^12^ Treated mice were allowed to reach their humane endpoint, which on average occurred 12–15 months after treatment. Encouragingly, we observed similar biochemical outcomes to those observed at the six-month time point, suggesting this therapy will likely provide long-term therapeutic benefit once translated to the clinic.^12^ Mice were treated at a pre-symptomatic time point to offer the greatest potential for disease correction before the onset of irreversible neurocognitive and peripheral damage. We have recently shown that MPSII mice transplanted at 4 months of age with HSGCT have worse outcomes compared with those transplanted at 2 months of age prior to symptom onset.^19^ Our recently opened phase I/II clinical trial is recruiting MPSII patients aged 4–12 months, which is before neuronopathic patients begin to miss cognitive milestones (ClinicalTrials.gov identifier NCT05665166).

In the brain, we observed 14% and 4% of WT IDS activity levels in the 12–15 month time point for IDS and IDS.ApoEII treatment groups, respectively, compared with 3.4% and 3.7% at the 6 month time point.^12^ The higher levels of brain IDS enzyme in the LV.IDS treatment group potentially explains why there is increased clearance of brain HS compared with mice in the LV.IDS.ApoEII group (4.3- vs. 2.8-fold reduction in MPSII brain HS storage, respectively). At the 6 month time point, the LV IDS cohort demonstrated no improvement in working memory,^12^ but given the superior IDS activity levels observed here at 10–13 months post-treatment, it is plausible that behavioral outcomes (measured at 6 months by Gleitz et al.^12^) could be improved by LV IDS if evaluated at a later time point. Despite overall lower VCN and commensurate IDS enzyme activity in the LV.IDS.ApoEII treatment group, compared with LV.IDS, brain HS sulfation patterning was corrected, astrogliosis and microglial activation were completely abrogated, and lysosomes were restored to normal compartment size, similar to the observation at the 6 month post-treatment time point and equivalent to (or more effectively so), in some brain regions, especially the amygdala, which is critical for behavior. In line with other clinical findings,^20^^,^^21^ brain IDS levels in our PoC study increase over time, from 3.4% to 14%, at 6 and 12–15 month time points, respectively. This suggests that continued engraftment of LV.IDS modified cells over time. In contrast, in the IDS.ApoEII group, enzyme levels remain constant at the 6 and 12–15 month time points, with 3.7% and 4% detected, respectively, which could potentially be explained by lower engraftment in this group, but a more likely explanation is a lower level of transduction in IDS.ApoEII in hematopoietic organs and similar changes in enzyme activity. The mice in each cohort were selected at random for either 6 month (Gleitz et al.^12^) or 12–15 month histological and pathological analysis (this study), so this could have been by chance.

Since our initial PoC study, other groups have also evaluated the potential of HSGCT for the treatment of MPSII. Smith et al.^22^ used an MPSII mouse model to evaluate the efficacy of *ex vivo* LV gene-modified HSPCs encoding a codon-optimized human IDS under the control of a strong constitutively active MNDU3 viral-derived promoter (MNDU3-IDS). In the study of Smith et al., 13% of WT IDS levels were achieved in the brain with GAG accumulation restored, comparable with our findings with LV.IDS in our long-term evaluations. However, weaker, but more specific, mammalian promoters such as CD11b or PGK may prove to be safer than viral-derived promoters such as MND, as they may be less likely to activate downstream genes on random genome integration. The MND promoter, despite a good safety record to date, may have been implicated in the recent trial halt reported for X-ALD, following the development of myelodysplastic syndrome in a single patient, which has since re-started.^23^^,^^24^

No reports of blood cancers have been reported in other HSCGT clinical trials using either the ubiquitous PGK or CD11b promoter, as used in our vector.^20^^,^^25^ We used the CD11b promoter for our vector, as it is a human specific promoter that restricts transgene expression to myeloid cells, some of which enter the brain and other organs. Together with the ApoEII BBB-crossing peptide, which helps direct enzyme distribution from the periphery toward the brain and improves bEND3 cell uptake by 5-fold, this combination of approaches, reduces the disparity between somatic enzyme activity levels and brain levels, which are typically in the order of 10- to 100-fold higher in somatic organs than the brain, and is potentially a more effective enzyme because of its improved uptake.^13^^,^^26^^,^^27^^,^^28^

As part of good pharmocovigilance practice, we evaluated potential toxicity of the LV.IDS.ApoEII drug product and treatment efficacy by performing histological analysis on the organs of treated and untreated MPSII and control WT mice. The study concluded that administration of either LV.IDS or LV.IDS.ApoEII treatment offered multisystem efficacy, in the attenuation of the MPSII phenotype. The adverse structural findings within the MPSII control cohort, most notably in the kidney and liver, were largely absent in tissues from WT-HSCT, LV.IDS, and LV.IDS.ApoEII treatment groups. Both the MPSII control group and the WT-HSCT treated group of mice displayed retinal atrophy, which is considered adverse, as it would likely result in significant visual impairment. However, in the LV.IDS- and LV.IDS.ApoEII-treated groups, the occurrence of retinal atrophy was profoundly reduced. This suggests that the therapeutic effect of both treatments extends across the blood-eye barrier, potentially mitigating the risk for sight impairment. Lenticular cataracts were identified in treatment groups in the study but were considered a secondary effect arising from the co-administration of busulfan rather than from administration of WT-HSCT, LV.IDS, or LV.IDS.ApoEII.^29^^,^^30^

To investigate inflammatory responses in MPSII and their correction following treatment, we chose to use a multiplex system, as multiple cytokine and chemokine profiles can be evaluated at once in a single reaction for each organ. In practice, we discovered this approach lacks sensitivity compared with the CBA flex set methodology used in our previous study.^12^ Despite this, we were able to identify interesting findings in the long-term-treated MPSII mice. In MPSII mice, the levels of MIP-1α, a marker of macrophage activation, were found to be elevated in the brain, heart, liver, lung, spleen, and kidney, indicating a state of heightened macrophage activity in these affected organs. However, all three treatment groups showed normalization of MIP-1α levels, except in the brain, in which WT-HSCT was ineffective compared with HSCGT using either LV.IDS or LV.IDS.ApoEII. Several studies have indicated that IL-9 is a cytokine commonly associated with inflammatory, allergic, and autoimmune diseases as well as parasitic infections and lack of IL-9 prolongs inflammation resolution in models of arthritis and ulcerative colitis.^31^^,^^32^^,^^33^^,^^34^ Here, IL-9 is specifically downregulated in MPSII livers. Recently a study focused on alcoholic liver injury (ALI) showed IL-9 downregulation in a mouse model of ALI, with concomitant secretion of hepatic macrophage pro-inflammatory factors, inflammatory cell infiltration, and the appearance of a large number of hepatic vacuoles, similar to those observed in MPSII livers.^35^ Our findings suggest that T cell polarization may be affected in MPSII, as indicated by decreased levels of specific cytokines in the liver. These include reduced levels of IL-9, typically produced by T helper type 9 (Th9) CD4^+^ T cells, as well as decreased IL-2, which is involved in T cell expansion. Additionally, IL-5, associated with Th2 CD4^+^ T cells, IL-10 produced by Foxp3^+^ regulatory T cells (Tregs), and IL-17A/KC produced by Th17 CD4^+^ T cells, were also found to be downregulated. T cells could potentially be becoming exhausted or may have an inability to polarize from their naive state in MPSII mice. Similarly, persistent stimulation of the immune system by viral and bacterial infection can induce T cell exhaustion, leading to loss of cytokine production and reduced immune cell function.^36^^,^^37^ Treg deficiency is reported in some chronic autoimmune deficiencies, and MPSII may exhibit similar traits.^38^^,^^39^

Interestingly, WT-HSCT treatment has positive biochemical benefit in certain organs, such as the lung, heart, spleen, and liver, in the context of normalizing β-hex activity, likely a consequence of IDS activity in these organs being close to WT levels. In contrast, WT-HSCT demonstrates limited effectiveness in correcting the brain and kidney. This could potentially be attributed to relatively low enzyme levels, possibly resulting from insufficient macrophage engraftment in the kidney. On the other hand, treatment with LV.IDS and LV.IDS.ApoEII shows notable improvements, approaching levels similar to those observed in unaffected individuals (WT levels). Neuronal cross-correction is prominently observed in this study, as has been previously documented in other HSGCT studies.^12^^,^^13^^,^^27^ The reduction in lysosomal compartment size toward normal levels is particularly evident in neurons of LV.IDS.ApoEII mice, as illustrated in Figure 5.

On the basis of the outcomes of this long-term PoC study and the findings obtained at the six-month time point, we effectively demonstrate the safety, efficacy, and durability of HSCGT treatment using IDS.ApoEII in the murine MPSII model. Subsequently, we have initiated a phase I/II clinical trial in MPSII patients using the IDS.ApoEII vector (ClinicalTrials.gov identifier NCT05665166). This is a first-in-human clinical trial to explore the safety, tolerability, and clinical efficacy of an *ex vivo* gene therapy for MPSII patients.

## Materials and methods

### Expression vectors

LVs containing the human IDS gene or human IDS gene tagged with ApoEII, under the CD11b promoter, were made as previously described.^12^

### LV production and titration

LV was produced as previously described^13^ by transient transfection of HEK293T cells with pRSV-Rev, pMDLg/pRRE, pMD2.G (Didier Trono, Addgene plasmids), and LV genome plasmid^13^^,^^40^^,^^41^^,^^42^ and 7.5 mM polyethylenimine (40 kDa; Polysciences^43^). In order to titer the vector, EL4 mouse lymphoma cells (TIB-39; American Type Culture Collection [ATCC], Manassas, VA) were transduced with a dilution series of concentrated LV for 6 h and transduced cells harvested 72 h later. The number of integrated viral genomes per cell, measured using a primer and probe set against HIV-1 ψ sequence, was determined using qPCR using a standard curve generated by dilutions of genomic DNA from an EL4 cell line containing 2 integrated copies/cell of pHRsin.SFFV.eGFP.att.wpre.^40^

### Mice and transplantation procedures

Female mice heterozygous for the X-linked allele on a C57BL/6 background were obtained from Prof. Joseph Muenzer (University of North Carolina at Chapel Hill) and bred with WT C57BL/6J males (Envigo, Alconbury, UK). MPSII mice were backcrossed onto the Pep3 CD45.1 congenic background (B6.SJL-Ptprc a Pepc b/BoyJ) to distinguish donor and recipient cells, as previously described.^40^ WT littermates were used as controls throughout. Mice were housed in individually ventilated cages with *ad libitum* access to food and water and were kept in a 12 h light/dark cycle. Male mice were used in this study and housed in groups of 2–5 with littermates.

For transplantation studies, total BM mononuclear cells from 6- to 12-week-old male MPSII mice (CD45.1^+^) were isolated from femurs and tibias and lineage-depleted using the murine lineage cell depletion kit (Miltenyi Biotec, Bisley, UK), as previously described.^13^ Cells were stimulated using 100 ng/mL murine stem cell factor, 100 ng/mL murine fms-like tyrosine kinase-3, and 10 ng/mL recombinant murine interleukin-3 (Peprotech, Rocky Hill, NJ) for 3 h prior to transduction with an LV for 24 h at a multiplicity of infection (MOI) of 100.

Six- to eight-week-old male MPSII mice housed in groups in autoclaved individually ventilated cages were myeloablated using 125 mg/kg busulfan (Busilvex; Pierre Fabre, Boulogne, France) in five daily doses (25 mg/kg/day) via intraperitoneal (i.p.) injection. Within 24 h of myeloablation, mice were injected with 3–4 × 10^5^ lineage-depleted transduced HSCs via the lateral tail vein. For WT transplants (WT-HSCT), busulfan-conditioned MPSII mice received 1–2 × 10^7^ untransduced total BM cells from 6- to 12-week-old WT donors (CD45.1^+^). No adverse effects were detected after engraftment. Engraftment of donor HSCs was assessed at 4 weeks post-transplantation in peripheral blood by flow cytometry, as previously described.^12^

### Sample processing

For this long-term study, mice were allowed to reach their humane endpoint before being culled and tissues harvested. The humane endpoint, as defined in our home office license, is the point at which mice started showing symptoms of sickness such as >15% weight loss, hair loss, lack of grooming or abnormal appearance, hunched posture, piloerection, lethargy on touching, facial grimace, respiratory distress, or neurological signs such as circling. The majority of treated and untreated MPSII mice demonstrated a gradual decline in health following the 6 month time point and demonstrating one or more of the above symptoms. A few mice in the long-term cohorts, including WT mice, demonstrated circling and were culled immediately and tissues harvested. MPSII controls and treated animals were harvested between 13 and 16 months on average. WT controls were typically harvested at 16–19 months. Six mice per group were anesthetized and transcardially perfused with 37°C PBS. One brain hemisphere was fixed in 4% paraformaldehyde (PFA) for 24 h and transferred to 30% sucrose and 2 mmol/L MgCl2/PBS solution for 48 h before freezing at −80°C. Samples of brain, spleen, heart, and liver were snap-frozen on dry ice. BM samples were collected by flushing one tibia and femur with 1 mL 2% fetal bovine serum (FBS)/PBS, filtered using a 70 μm cell strainer and lysed using red blood cell lysis buffer (150 mM NH_4_Cl, 10 mM KHCO_3_, 0.1 mM EDTA [pH 7.2–7.4]). For enzyme activity assays, samples were homogenized and sonicated in homogenization buffer (0.5 M NaCl, 0.02 M Tris, 0.1% Triton X-100 [pH 7]). Genomic DNA used for organ VCN analysis was extracted using the GenElute Mammalian Genomic DNA Miniprep kit (Sigma-Aldrich).

### Enzyme activity assays

IDS enzyme activity was measured in a two-step protocol using the fluorescent substrate MU-αIdoA-2S (Carbosynth) and Aldurazyme (Genzyme), as previously described.^44^ Starting material was standardized to 20 μg total protein or plasma; 40 μg for liver, heart, lung, spleen, and BM; and 60 μg for brain using a BCA assay (Thermo Fisher Scientific). For β-hexosaminidase activity, 1 μg total protein from brain or 2 μg from spleen and plasma were added to 0.5 mM 4-methylumbelliferyl-N-acetyl-β-d-glucosaminide substrate (Sigma-Aldrich), incubated for 40 min at 37°C, and stopped with 200 μL of 0.2 M carbonate buffer. Fluorescence was measured using the BioTek Synergy HT plate reader (excitation 360 nm, emission 460 nm).

### Immunohistochemistry

Free-floating immunohistochemistry (IHC) was performed on 30 μm PFA-fixed coronal brain sections using rabbit anti-NeuN (1:1,000; ab177487; Abcam), rabbit anti-GFAP (1:1,500; Z0334; Dako, Stockport, UK), and rat anti-LAMP2 (1:500; ab13524; Abcam) primary antibodies using standard protocols.^12^ ILB4 (5 μg/mL; L5391; Sigma-Aldrich) was visualized on 30 μm coronal brain sections using DAB substrate for 40 s (Vector, Peterborough, UK) using standard protocols.^13^ Images were acquired on a 3DHISTECH Pannoramic-250 microscope slide-scanner using a 20×/0.30 Plan Achromat objective (Zeiss) with extended focus and the DAPI, fluorescein isothiocyanate (FITC), and tetramethyl rhodamine (TRITC) filter sets. Snapshots of the slide-scans were taken using CaseViewer software (3DHISTECH). Nonlinear adjustments were made to all immunofluorescence images equally to eliminate background: gamma 0.72 and input levels of 0–190. GFAP immunofluorescence was quantified using ImageJ software on four sections per mouse for cortex, 2 sections per mouse for striatum and one section per mouse for the hippocampus and amygdala (n = 3–6/group). Counts of ILB4-positive cells were performed on four sections per mouse (n = 3–6/group) at 20× magnification and counted manually using ImageJ software. LAMP2 area quantification was performed using CellProfiler software on 40× images capture using CaseViewer. H&E staining was performed at Histologix using validated GLP protocols.

### Glycosaminoglycan analysis

Soluble brain and liver fractions were collected and processed as previously described.^45^ HS chains were digested using 0.2 mI/U each of heparinase I, II, and III, and CS/DS chains were digested using 2 mI/U chondroitinase ABC (Sigma-Aldrich) in 50 mM Tris/50 mM NaCl (pH 7.9). Resulting disaccharides were freeze-dried and 2-aminoacridone (AMAC) labeled. HS and CS/DS disaccharides were separated by reverse-phase high-performance liquid chromatography (HPLC) using a Zorbax Eclipse XDB-C18 column (4.6 × 100 mm, 3.5 μm; Agilent), equilibrated in 95% 0.1 M ammonium acetate/5% acetonitrile on an Agilent 1200 Series HPLC system. Disaccharides were eluted over 5%–20% acetonitrile gradient at 0.2 mL/min. AMAC-labeled HS and CS/DS disaccharide standards (Iduron) were used for peak identification. Total fluorescence was compared with known quantities of HS or CS/DS to calculate absolute amounts of each disaccharide. Correction factors were calculated as described.^12^

### VCN analysis

To determine the VCN in mice tissues, qDNA was extracted using GenElute Mammalian Genomic DNA Miniprep kit (Sigma-Aldrich) and number of vector integrations determined using qPCR using the WPRE and rodent GAPDH primer probe sets described,^46^ and a standard curve was generated by serial dilution of DNA sample derived from an EL4 cell line clone (ALS EL4 eGFP 2.2) containing two copies of integrated WPRE gene/cell.^13^

### Bio-Plex multiplex immunoassay

The expression levels of mouse cytokines, chemokines, and growth factors were quantified using Bio-Plex Pro Mouse Cytokine 23-plex Assay kit (M60009RDPD; Bio-Rad), according to the manufacturer’s instructions and read on the Bio-Plex 200 suspension array system using Luminex technology. Tissue samples were homogenized in 100 μL homogenization buffer (0.5 M NaCl, 0.02 M Tris [pH 7]) using a motor pestle in 1.5 mL Eppendorf tubes. The samples were then further processed by adding 100 μL homogenization buffer plus 0.1% Triton X-100 and sonicated on ice for 3 × 5 s at 5 μm amplitude using an MSE Soniprep 150 Plus Ultrasonic Disintegrator. Homogenized samples were then centrifuged at 2000 × *g* at 4°C for 20 min, and the supernatant was eluted and stored at −80°C until required. Prior to loading, protein concentrations were quantified using BCA assay (Thermo Fisher Scientific) and loaded at an optimized protein concentration of 5,000 μg. The data produced were analyzed using the Bio-Plex software manager.

### Study approval

Animal experiments were ethically approved by the Manchester Research Ethics Committee and performed under UK Home Office regulations and PPLs 40/3658 and POC3AEEB0.

### Statistics

Statistical analysis was performed using Prism 9 software (GraphPad, La Jolla, CA). One-way or two-way ANOVA was performed for multi-group analysis followed by Tukey’s multi-comparisons test. Significance was set at p < 0.05. MPSII mutant mice and WT littermates were randomly assigned to control or transplantation groups, although transplantation group allocation was also partially determined by the number of donor animals and the amount of cells available for transplant.

## Data and code availability

Raw data were generated at University of Manchester. Derived data supporting the findings of this study are available from the corresponding author (B.W.B.) on request if authorized by our study sponsor.

## Supplemental information

Document S1. Figure S1 and Tables S1–S6

Document S2. Article plus supplemental information
