# Supplementary material for: Sustained long-term disease correction in a murine model of MPSII following stem cell gene therapy
Source: Mol Ther Methods Clin Dev. 2023 Oct 20;31:101127. doi: 10.1016/j.omtm.2023.101127 (PMC10618237; doi:10.1016/j.omtm.2023.101127)
Supplement: Document S1. Figure S1 and Tables S1–S6 [file mmc1.pdf]

OMTM, Volume *31*

Supplemental information

Sustained long-term disease correction in a murine model of MPSII following stem cell gene therapy

Stuart Ellison, Aiyin Liao, Hélène F.E. Gleitz, Helen Parker, Laura Booth, John Robinson, Shaun Wood, Jessica Taylor, Rebecca Holley, and Brian W. Bigger

Immunohistochemical findings by HistologiX

The eye, heart, kidney, liver, spleen and brain slides from treated and untreated mice underwent the standard HistologiX H&E staining protocol.

All H&E stained slides were checked for quality and repeated if required standards were not met.

Pathologist Review

Note: GLP compliance is not claimed for the Pathologist review.

Examination of the stained slides was conducted by a British (FRCPath) and American (Dipl.ACVP) board-certified Histopathologist at PathCelerate. Full toxicologic evaluation of tissues, including assessment of MPSII-mediated morphological change were recorded based upon morphology, severity, and distribution.

Semi-quantitative manual counting was performed on blood smears, with 100 white blood cells counted to give a differential lineage calculation. Undifferentiated lymphoblast quantities were also recorded.

1) Blood smears

Where evaluable, blood smears from all control and treated groups did not show evidence of test article-(inclusive of busulfan) related abnormality. Rare, scattered, and undifferentiated monocytes with expanded, foamy, cytoplasm, containing granular debris and similar in nature to a morphology consistent with “Alder anomaly”, were noted within the blood smears of animals in the MPSII control group, and, to a lesser degree, amongst animals in the WT-HSCT treatment group (Table S1 and Figure S1). This finding was considered to reflect the intra-cellular storage of GAG/breakdown product, amongst white blood cells of various lineage (Bhuyan et al., 2013).

Table S1: Blood smears – Pathologist’s comments

Group	Study	Mouse ID	Pathologist’s Comments
Wild-type	Medium Term (6 months)	238	NA
		239	NA
		240	NA
		265	NA
		266	NA
		267	NA
		286	Neutrophils (mature): 33; Neutrophils (immature): 8; Lymphocytes (mature): 59
	Long Term (12-16 months)	257	Neutrophils (mature): 25; Neutrophils (immature): 18; Lymphocytes (mature): 57
		261	Neutrophils (mature): 18; Neutrophils (immature): 27; Lymphocytes (mature): 55
		262	Neutrophils (mature): 31; Neutrophils (immature): 13; Lymphocytes (mature): 56
		271	Neutrophils (mature): 22; Neutrophils (immature): 12; Lymphocytes (mature): 66
		313	Neutrophils (mature): 24; Neutrophils (immature): 25; Lymphocytes (mature): 51
		328	Neutrophils (mature): 30; Neutrophils (immature): 15; Lymphocytes (mature): 55
		329	Neutrophils (mature): 31; Neutrophils (immature): 25; Lymphocytes (mature): 44
MPSII	Medium Term (6 months)	270	NA
		278	NA
		327	SI (cell number)
		332	Neutrophils (mature): 33; Neutrophils (immature): 17; Lymphocytes (mature): 23;<br>Undifferentiated Lymphocytes/Monocytes (Alder Anomaly); 27
		333	SI (cell number) (Evidence in scattered cells with Alder Anomaly)
		334	Neutrophils (mature): 24; Neutrophils (immature): 12; Lymphocytes (mature): 21;<br>Undifferentiated Lymphocytes/Monocytes (Alder Anomaly); 43
	Long Term (12-16 months)	256	SI (preservation) (Evidence in scattered cells with Alder Anomaly)
		259	Neutrophils (mature): 40; Neutrophils (immature): 18; Lymphocytes (mature): 36;<br>Undifferentiated Lymphocytes/Monocytes (Alder Anomaly); 6
		260	Neutrophils (mature): 24; Neutrophils (immature): 25; Lymphocytes (mature): 39;<br>Undifferentiated Lymphocytes/Monocytes (Alder Anomaly); 12
		346	Neutrophils (mature): 33; Neutrophils (immature): 17; Lymphocytes (mature): 30;<br>Undifferentiated Lymphocytes/Monocytes (Alder Anomaly); 20
		348	Neutrophils (mature): 35; Neutrophils (immature): 25; Lymphocytes (mature): 28;<br>Undifferentiated Lymphocytes/Monocytes (Alder Anomaly); 12
WT-HSCT	Medium Term (6 months)	285	NA
		287	NA
		317	Neutrophils (mature): 26; Neutrophils (immature): 38; Lymphocytes (mature): 30;<br>Undifferentiated Lymphocytes/Monocytes (Alder Anomaly); 6

Group	Study	Mouse ID	Pathologist's Comments
		320	Neutrophils (mature): 28; Neutrophils (immature): 42; Lymphocytes (mature): 26; Undifferentiated Lymphocytes/Monocytes (Alder Anomaly); 4
		321	Neutrophils (mature): 35; Neutrophils (immature): 20; Lymphocytes (mature): 27; Undifferentiated Lymphocytes/Monocytes (Alder Anomaly); 18
		323	SI (cell number)
	Long Term (12-16 months)	301	Neutrophils (mature): 39; Neutrophils (immature): 27; Lymphocytes (mature): 29; Undifferentiated Lymphocytes/Monocytes (Alder Anomaly); 5
		302	Neutrophils (mature): 27; Neutrophils (immature): 45; Lymphocytes (mature): 22; Undifferentiated Lymphocytes/Monocytes (Alder Anomaly); 6
		309	Neutrophils (mature): 30; Neutrophils (immature): 31; Lymphocytes (mature): 39
		310	SI (preservation) (Evidence in rare cells with Alder Anomaly)
		314	NA
		315	SI (preservation) (Evidence in rare cells with Alder Anomaly)
		316	Neutrophils (mature): 32; Neutrophils (immature): 28; Lymphocytes (mature): 40
		421	Neutrophils (mature): 25; Neutrophils (immature): 41; Lymphocytes (mature): 34
		422	Neutrophils (mature): 24; Neutrophils (immature): 54; Lymphocytes (mature): 19; Undifferentiated Lymphocytes/Monocytes (Alder Anomaly); 3
LV.IDS	Medium Term (6 months)	324	Neutrophils (mature): 47; Neutrophils (immature): 26; Lymphocytes (mature): 27
		325	Neutrophils (mature): 35; Neutrophils (immature): 45; Lymphocytes (mature): 20
		361	NA
		362	NA
		363	NA
		364	NA
	Long Term (12-16 months)	355	NA
		341	Neutrophils (mature): 38; Neutrophils (immature): 18; Lymphocytes (mature): 44
		342	Neutrophils (mature): 20; Neutrophils (immature): 20; Lymphocytes (mature): 60
		350	Neutrophils (mature): 26; Neutrophils (immature): 15; Lymphocytes (mature): 59
		357	SI (cell number)
		386	NA
LV.IDS.ApoEII	Medium Term (6 months)	397	Neutrophils (mature): 27; Neutrophils (immature): 22; Lymphocytes (mature): 51
		399	Neutrophils (mature): 18; Neutrophils (immature): 34; Lymphocytes (mature): 48
		406	Neutrophils (mature): 31; Neutrophils (immature): 38; Lymphocytes (mature): 31
		407	Neutrophils (mature): 28; Neutrophils (immature): 20; Lymphocytes (mature): 52
		408	Neutrophils (mature): 16; Neutrophils (immature): 19; Lymphocytes (mature): 65
		409	Neutrophils (mature): 22; Neutrophils (immature): 20; Lymphocytes (mature): 58
	Long Term (12-16 months)	4060	NA
		424	NA
		425	NA
		426	NA
		452	NA
		454	NA
		465	NA

2) Eye

Where available, eye samples from individual animals in all groups co-administered busulfan showed evidence of cataract formation, ranging from minimal-to-moderate in severity, and characterized by lenticular fiber fragmentation, with sporadic epithelial hypertrophy/hyperplasia, and occasional Morgagnian globule formation. These were considered a secondary and adverse effect arising from the co-administration of busulfan (Fraunfelder & Meyer, 1983).

Table S2: Eye – Pathologist's comments

Group	Study	Mouse ID	Pathologist's Comments
Wild-type	Medium Term (6 months)	238	NA
		239	NA
		240	NA
		265	NA
		266	NA
		267	NA
		286	NA
	Long Term (12-16 months)	257	NA
		261	NA
		262	NA
		271	NA
		313	-
		328	-
		329	-
MPSII	Medium Term (6 months)	270	NA
		278	NA
		327	NA
		332	NA

Group	Study	Mouse ID	Pathologist's Comments
		333	NA
		334	NA
	Long Term (12-16 months)	256	Atrophy, retina (outer nuclear layer); 2 d
		259	Atrophy, retina (outer nuclear layer); 3 d
		260	Atrophy, retina (outer nuclear layer); 3 d
		346	Atrophy, retina (outer nuclear layer); 2 d
		348	Atrophy, retina (outer nuclear layer); 3 d
WT-HSCT	Medium Term (6 months)	285	NA
		287	NA
		317	NA
		320	NA
		321	NA
		323	NA
	Long Term (12-16 months)	301	NA
		302	NA
		309	Atrophy, retina (outer nuclear layer); 2 d. Cataract, lenticular; 3 mf
		310	Atrophy, retina (outer nuclear layer); 2 d. Cataract, lenticular; 1 mf
		314	Atrophy, retina (outer nuclear layer); 3 d. Cataract, lenticular; 1 f
		315	Atrophy, retina (outer nuclear layer); 2 d. Cataract, lenticular; 1 mf
		316	Atrophy, retina (outer nuclear layer); 1 d
		421	NA
		422	NA
LV.IDS	Medium Term (6 months)	324	NA
		325	NA
		361	-
		362	Cataract, lenticular; 1 f
		363	Atrophy, retina (outer nuclear layer); 1 d. Cataract, lenticular; 1 mf
		364	Atrophy, retina (outer nuclear layer); 1 d. Cataract, lenticular; 2 mf
	Long Term (12-16 months)	355	Atrophy, retina (outer nuclear layer); 1 d
		341	NA
		342	NA
		350	Atrophy, retina (outer nuclear layer); 1 d. Cataract, lenticular; 1 mf
		357	NA
		386	Atrophy, retina (outer nuclear layer); 1 d. Cataract, lenticular; 2 mf
LV.IDS.ApoEII	Medium Term (6 months)	397	Cataract, lenticular; 1 mf
		399	Atrophy, retina (outer nuclear layer); 1 d. Cataract, lenticular; 2 mf
		406	Atrophy, retina (outer nuclear layer); 1 d. Cataract, lenticular; 1 mf
		407	Cataract, lenticular; 1 mf
		408	Cataract, lenticular; 1 mf
		409	Cataract, lenticular; 1 mf
	Long Term (12-16 months)	4060	Atrophy, retina (outer nuclear layer); 1 d. Cataract, lenticular; 2 mf
		424	Atrophy, retina (outer nuclear layer); 1 d. Cataract, lenticular; 1 f
		425	Atrophy, retina (outer nuclear layer); 1 d. Cataract, lenticular; 1 mf
		426	Atrophy, retina (outer nuclear layer); 1 d. Cataract, lenticular; 1 mf
		452	Atrophy, retina (outer nuclear layer); 1 d. Cataract, lenticular; 2 mf
		454	Atrophy, retina (outer nuclear layer); 1 d. Cataract, lenticular; 2 mf
		465	Cataract, lenticular; 1 mf

3) Heart

Minimal interstitial aggregates of vacuolated macrophages were observed in animals from the MPSII group, at both time-points, in isolation.

Table S3: Heart - Pathologist's Comments

Group	Study	Mouse ID	Pathologist's Comments
	Medium Term (6 months)	238	-
		239	-
		240	-
		265	NA
		266	NA
		267	NA
		286	NA
	Long Term (12-16 months)	257	-
		261	-
		262	-
		271	-
		313	-
		328	-
		329	-
MPSII		270	NA
		278	NA

Group	Study	Mouse ID	Pathologist's Comments
	Medium Term (6 months)	327	Infiltrate, vacuolated macrophages, interstitium; 1 mf
		332	Infiltrate, vacuolated macrophages, interstitium; 1 mf
		333	NA
		334	Infiltrate, vacuolated macrophages, interstitium; 1 mf
	Long Term (12-16 months)	256	Infiltrate, vacuolated macrophages, interstitium; 1 mf
		259	Infiltrate, vacuolated macrophages, interstitium; 1 mf
		260	Infiltrate, vacuolated macrophages, interstitium; 1 mf
		346	Infiltrate, vacuolated macrophages, interstitium; 1 mf
		348	Infiltrate, vacuolated macrophages, interstitium; 1 mf
WT-HSCT	Medium Term (6 months)	285	-
		287	-
		317	-
		320	-
		321	-
		323	-
	Long Term (12-16 months)	301	-
		302	-
		309	-
		310	-
		314	-
		315	-
		316	-
		421	-
		422	Inflammation, vessel; 1 f
LV.IDS	Medium Term (6 months)	324	-
		325	-
		361	-
		362	-
		363	-
		364	-
	Long Term (12-16 months)	355	-
		341	-
		342	-
		350	-
		357	NA
		386	-
LV.IDS.ApoEII	Medium Term (6 months)	397	-
		399	-
		406	-
		407	-
		408	-
		409	-
	Long Term (12-16 months)	4060	Inflammation, vessel; 1 f
		424	-
		425	-
		426	-
		452	-
		454	-
		465	-

4) Kidney

Amongst MPSII mice, there was a spectrum of findings associated with the intra-cellular accumulation of GAG. This encompassed minimal-to-slight accumulations of vacuolated macrophages/histiocytes within the interstitium, spreading to involve the renal tubular epithelium, and glomeruli. At the 12-16 month time-point, there was accompanying inflammatory cell infiltration around local vascular profiles, indicative of mild inflammation (nephritis). In all treatment groups, and at both time-points, this spectrum of findings was attenuated, with an increased level of efficacy noted amongst animals administered LV.IDS or LV.IDS.ApoEII.

Table S4: Kidney- Pathologist's Comments

Group	Study	Mouse ID	Pathologist's Comments
Wild-type	Medium Term (6 months)	238	-
		239	-
		240	Infiltrate, inflammatory cell; 1 f
		265	Infiltrate, inflammatory cell; 1 f
		266	-
		267	Basophilia, tubule; 1 f
		286	NA
		257	-

Group	Study	Mouse ID	Pathologist’s Comments
	Long Term (12-16 months)	261	Basophilia, tubule; 1 f
	Long Term (12-16 months)	262	-
	Long Term (12-16 months)	271	Infiltrate, inflammatory cell; 1 f
	Long Term (12-16 months)	313	-
	Long Term (12-16 months)	328	-
	Long Term (12-16 months)	329	-
MPSII	Medium Term (6 months)	270	Vacuolation, glomerular; 1 mf. Vacuolation, tubular; 2 mf. Infiltrate, vacuolated macrophages, interstitium; 1 mf
MPSII	Medium Term (6 months)	278	Infiltrate, vacuolated macrophages, interstitium; 1 mf. Infiltrate, inflammatory cell; 1 f
MPSII	Medium Term (6 months)	327	Vacuolation, glomerular; 1 mf. Vacuolation, tubular; 2 mf. Infiltrate, vacuolated macrophages, interstitium; 1 mf
MPSII	Medium Term (6 months)	332	Vacuolation, glomerular; 1 mf. Vacuolation, tubular; 2 mf. Infiltrate, vacuolated macrophages, interstitium; 1 mf
MPSII	Medium Term (6 months)	333	Vacuolation, glomerular; 1 mf. Infiltrate, vacuolated macrophages, interstitium; 1 mf
MPSII	Medium Term (6 months)	334	Vacuolation, glomerular; 1 mf. Vacuolation, tubular; 1 mf. Infiltrate, vacuolated macrophages, interstitium; 1 mf. Infiltrate, inflammatory cell; 1 f
MPSII	Long Term (12-16 months)	256	Vacuolation, glomerular; 1 mf. Vacuolation, tubular; 2 mf. Infiltrate, vacuolated macrophages, interstitium; 2 mf. Infiltrate, inflammatory cell, perivascular; 1 mf
MPSII	Long Term (12-16 months)	259	Vacuolation, glomerular; 1 mf. Vacuolation, tubular; 1 mf. Infiltrate, vacuolated macrophages, interstitium; 1 mf. Infiltrate, inflammatory cell, perivascular; 1 mf
MPSII	Long Term (12-16 months)	260	Vacuolation, glomerular; 1 mf. Vacuolation, tubular; 1 mf. Infiltrate, vacuolated macrophages, interstitium; 2 mf. Infiltrate, inflammatory cell, perivascular; 1 f
MPSII	Long Term (12-16 months)	346	Vacuolation, glomerular; 1 mf. Vacuolation, tubular; 1 mf. Infiltrate, vacuolated macrophages, interstitium; 1 mf. Infiltrate, inflammatory cell, perivascular; 1 f
MPSII	Long Term (12-16 months)	348	NA
WT-HSCT	Medium Term (6 months)	285	Vacuolation, tubular; 1 mf
WT-HSCT	Medium Term (6 months)	287	-
WT-HSCT	Medium Term (6 months)	317	Vacuolation, glomerular; 1 mf. Vacuolation, tubular; 1 mf
WT-HSCT	Medium Term (6 months)	320	Vacuolation, glomerular; 1 mf. Vacuolation, tubular; 1 mf. Infiltrate, inflammatory cell; 1 f
WT-HSCT	Medium Term (6 months)	321	Vacuolation, tubular; 1 mf. Infiltrate, inflammatory cell; 1 f
WT-HSCT	Medium Term (6 months)	323	Vacuolation, tubular; 1 mf. Infiltrate, inflammatory cell; 1 f
WT-HSCT	Long Term (12-16 months)	301	Vacuolation, glomerular; 1 mf. Vacuolation, tubular; 1 mf. Infiltrate, inflammatory cell; 1 f
WT-HSCT	Long Term (12-16 months)	302	Vacuolation, tubular; 1 mf. Infiltrate, inflammatory cell; 1 f
WT-HSCT	Long Term (12-16 months)	309	Vacuolation, tubular; 1 mf. Infiltrate, inflammatory cell; 1m f
WT-HSCT	Long Term (12-16 months)	310	Vacuolation, tubular; 1 mf. Infiltrate, inflammatory cell; 1 f
WT-HSCT	Long Term (12-16 months)	314	Vacuolation, tubular; 1 mf. Infiltrate, inflammatory cell; 1 f
WT-HSCT	Long Term (12-16 months)	315	Vacuolation, glomerular; 1 mf. Vacuolation, tubular; 1 mf. Basophilia, tubule; 1 f
WT-HSCT	Long Term (12-16 months)	316	Vacuolation, tubular; 1 mf
WT-HSCT	Long Term (12-16 months)	421	Vacuolation, tubular; 1 mf. Infiltrate, inflammatory cell; 1 f
WT-HSCT	Long Term (12-16 months)	422	Vacuolation, glomerular; 1 mf. Vacuolation, tubular; 2 mf. Infiltrate, inflammatory cell; 1 mf
LV.IDS	Medium Term (6 months)	324	Infiltrate, inflammatory cell; 1 f
LV.IDS	Medium Term (6 months)	325	Vacuolation, tubular; 1 mf. Infiltrate, inflammatory cell; 1 mf
LV.IDS	Medium Term (6 months)	361	Vacuolation, tubular; 1 mf. Infiltrate, inflammatory cell; 1 mf
LV.IDS	Medium Term (6 months)	362	Vacuolation, tubular; 1 mf
LV.IDS	Medium Term (6 months)	363	Vacuolation, tubular; 1 mf. Infiltrate, inflammatory cell; 1 mf
LV.IDS	Medium Term (6 months)	364	Infiltrate, inflammatory cell; 1 f
LV.IDS	Long Term (12-16 months)	355	Infiltrate, inflammatory cell; 1 mf
LV.IDS	Long Term (12-16 months)	341	Infiltrate, inflammatory cell; 1 f
LV.IDS	Long Term (12-16 months)	342	Vacuolation, tubular; 1 mf. Infiltrate, inflammatory cell; 1 mf
LV.IDS	Long Term (12-16 months)	350	Vacuolation, glomerular; 1 mf. Vacuolation, tubular; 1 mf. Infiltrate, inflammatory cell; 1 mf. Basophilia, tubule; 1 f
LV.IDS	Long Term (12-16 months)	357	NA
LV.IDS	Long Term (12-16 months)	386	Vacuolation, tubular; 1 mf. Basophilia, tubule; 1 f
LV.IDS.ApoEII	Medium Term (6 months)	397	Vacuolation, tubular; 1 mf
LV.IDS.ApoEII	Medium Term (6 months)	399	Vacuolation, tubular; 1 mf. Infiltrate, inflammatory cell; 1 mf
LV.IDS.ApoEII	Medium Term (6 months)	406	Vacuolation, tubular; 1 mf. Infiltrate, inflammatory cell; 1 mf
LV.IDS.ApoEII	Medium Term (6 months)	407	Infiltrate, inflammatory cell; 1 mf
LV.IDS.ApoEII	Medium Term (6 months)	408	Vacuolation, tubular; 1 mf
LV.IDS.ApoEII	Medium Term (6 months)	409	Vacuolation, tubular; 1 mf. Infiltrate, inflammatory cell; 1 mf
LV.IDS.ApoEII	Long Term (12-16 months)	4060	Infiltrate, inflammatory cell; 1 mf
LV.IDS.ApoEII	Long Term (12-16 months)	424	Infiltrate, inflammatory cell; 1 f
LV.IDS.ApoEII	Long Term (12-16 months)	425	Vacuolation, tubular; 1 mf. Infiltrate, inflammatory cell; 1 mf
LV.IDS.ApoEII	Long Term (12-16 months)	426	Infiltrate, inflammatory cell; 1 mf
LV.IDS.ApoEII	Long Term (12-16 months)	452	Infiltrate, inflammatory cell; 1 f
LV.IDS.ApoEII	Long Term (12-16 months)	454	Vacuolation, tubular; 1 mf. Infiltrate, inflammatory cell; 1 mf
LV.IDS.ApoEII	Long Term (12-16 months)	465	Vacuolation, tubular; 1 mf

5) Liver

Amongst MPSII mice, there were a spectrum of findings associated with the intra-cellular accumulation of GAG. This encompassed minimal-to-slight accumulations of vacuolated macrophages/histiocytes within the interstitium, spreading to involve hepatocytes, at minimal-to-moderate severity. At the 12-16 month time-point, there was accompanying minimal-to-slight hepatocellular necrosis. In all treatment groups, and at both time-points, this spectrum of findings was attenuated, with findings of minimal-to-slight hepatocellular vacuolation, in isolation.

Table S5: Liver- Pathologist's Comments

Group	Study	Mouse ID	Pathologist's Comments
Wild-type	Medium Term (6 months)	238	Infiltrate, inflammatory cell; 1 f
Wild-type	Medium Term (6 months)	239	-
Wild-type	Medium Term (6 months)	240	Infiltrate, inflammatory cell; 1 f
Wild-type	Medium Term (6 months)	265	-
Wild-type	Medium Term (6 months)	266	-
Wild-type	Medium Term (6 months)	267	-
Wild-type	Medium Term (6 months)	286	NA
Wild-type	Long Term (10-12 months)	257	Accumulation, fat, hepatocyte; 1 d. Infiltrate, inflammatory cell; 1 f
Wild-type	Long Term (10-12 months)	261	Accumulation, fat, hepatocyte; 2 d. Infiltrate, inflammatory cell; 1 mf
Wild-type	Long Term (10-12 months)	262	Accumulation, fat, hepatocyte; 1 mf
Wild-type	Long Term (10-12 months)	271	Accumulation, fat, hepatocyte; 1 mf. Infiltrate, inflammatory cell; 1 f
Wild-type	Long Term (10-12 months)	313	Accumulation, fat, hepatocyte; 2 d. Infiltrate, inflammatory cell; 1 mf
Wild-type	Long Term (10-12 months)	328	Accumulation, fat, hepatocyte; 2 d
Wild-type	Long Term (10-12 months)	329	Accumulation, fat, hepatocyte; 2 mf
MPSII	Medium Term (6 months)	270	Vacuolation; hepatocyte; 1 d. Infiltrate, vacuolated macrophages, interstitium; 1 mf
MPSII	Medium Term (6 months)	278	Vacuolation; hepatocyte; 1 mf. Infiltrate, vacuolated macrophages, interstitium; 1 mf
MPSII	Medium Term (6 months)	327	Vacuolation; hepatocyte; 2 d. Infiltrate, vacuolated macrophages, interstitium; 1 mf
MPSII	Medium Term (6 months)	332	Vacuolation; hepatocyte; 2 d. Infiltrate, vacuolated macrophages, interstitium; 1 mf
MPSII	Medium Term (6 months)	333	Infiltrate, vacuolated macrophages, interstitium; 1 mf
MPSII	Medium Term (6 months)	334	Vacuolation; hepatocyte; 1 d. Infiltrate, vacuolated macrophages, interstitium; 1 mf
MPSII	Long Term (10-12 months)	256	Vacuolation; hepatocyte; 3 d. Infiltrate, vacuolated macrophages, interstitium; 2 mf. Necrosis, hepatocyte; 1 mf
MPSII	Long Term (10-12 months)	259	Vacuolation; hepatocyte; 1 d. Infiltrate, vacuolated macrophages, interstitium; 1 mf. Necrosis, hepatocyte; 1 mf
MPSII	Long Term (10-12 months)	260	Vacuolation; hepatocyte; 1 mf. Infiltrate, vacuolated macrophages, interstitium; 2 mf. Necrosis, hepatocyte; 2 mf
MPSII	Long Term (10-12 months)	346	Vacuolation; hepatocyte; 2 d. Infiltrate, vacuolated macrophages, interstitium; 2 mf. Necrosis, hepatocyte; 1 f
MPSII	Long Term (10-12 months)	348	Vacuolation; hepatocyte; 1 mf. Infiltrate, vacuolated macrophages, interstitium; 2 mf. Necrosis, hepatocyte; 1 f
WT-HSCT	Medium Term (6 months)	285	Infiltrate, inflammatory cell; 1 f
WT-HSCT	Medium Term (6 months)	287	-
WT-HSCT	Medium Term (6 months)	317	Vacuolation; hepatocyte; 1 d
WT-HSCT	Medium Term (6 months)	320	Vacuolation; hepatocyte; 1 d. Infiltrate, inflammatory cell; 1 f
WT-HSCT	Medium Term (6 months)	321	Infiltrate, inflammatory cell; 1 f
WT-HSCT	Medium Term (6 months)	323	Vacuolation; hepatocyte; 1 d. Infiltrate, inflammatory cell; 1 f
WT-HSCT	Long Term (10-12 months)	301	Infiltrate, inflammatory cell; 1 f
WT-HSCT	Long Term (10-12 months)	302	Infiltrate, inflammatory cell; 1mf
WT-HSCT	Long Term (10-12 months)	309	Vacuolation; hepatocyte; 1 d.
WT-HSCT	Long Term (10-12 months)	310	Vacuolation; hepatocyte; 1 mf. Infiltrate, inflammatory cell; 1mf
WT-HSCT	Long Term (10-12 months)	314	Vacuolation; hepatocyte; 1 d
WT-HSCT	Long Term (10-12 months)	315	Vacuolation; hepatocyte; 2 d
WT-HSCT	Long Term (10-12 months)	316	Vacuolation; hepatocyte; 1 d
WT-HSCT	Long Term (10-12 months)	421	Vacuolation, hepatocyte; 1 d. Infiltrate, inflammatory cell; 1 f
WT-HSCT	Long Term (10-12 months)	422	Vacuolation, hepatocyte; 1 mf. Infiltrate, inflammatory cell; 1 mf
LV.IDS	Medium Term (6 months)	324	-
LV.IDS	Medium Term (6 months)	325	Vacuolation; hepatocyte; 1 d
LV.IDS	Medium Term (6 months)	361	Vacuolation; hepatocyte; 1 d
LV.IDS	Medium Term (6 months)	362	Vacuolation; hepatocyte; 1 d
LV.IDS	Medium Term (6 months)	363	Vacuolation; hepatocyte; 1 d
LV.IDS	Medium Term (6 months)	364	-
LV.IDS	Long Term (10-12 months)	355	Vacuolation; hepatocyte; 1 d
LV.IDS	Long Term (10-12 months)	341	Vacuolation; hepatocyte; 1 d
LV.IDS	Long Term (10-12 months)	342	Vacuolation; hepatocyte; 1 d. Infiltrate, inflammatory cell; 1 f
LV.IDS	Long Term (10-12 months)	350	Vacuolation; hepatocyte; 1 d. Infiltrate, inflammatory cell; 1 f
LV.IDS	Long Term (10-12 months)	357	NA
LV.IDS	Long Term (10-12 months)	386	Vacuolation; hepatocyte; 1 d
LV.IDS.ApoEII	Medium Term (6 months)	397	Vacuolation; hepatocyte; 1 d
LV.IDS.ApoEII	Medium Term (6 months)	399	Vacuolation; hepatocyte; 1 d
LV.IDS.ApoEII	Medium Term (6 months)	406	Vacuolation; hepatocyte; 1 d
LV.IDS.ApoEII	Medium Term (6 months)	407	Infiltrate, inflammatory cell; 1 mf
LV.IDS.ApoEII	Medium Term (6 months)	408	Infiltrate, inflammatory cell; 1 f

Group	Study	Mouse ID	Pathologist's Comments
		409	Infiltrate, inflammatory cell; 1 f
	Long Term (10-12 months)	4060	Vacuolation; hepatocyte; 1 d
		424	Vacuolation; hepatocyte; 2 d. Infiltrate, inflammatory cell; 1 mf
		425	Vacuolation; hepatocyte; 1 d
		426	Vacuolation; hepatocyte; 1 d
		452	Vacuolation; hepatocyte; 1 d
		454	Vacuolation; hepatocyte; 1 d. Infiltrate, inflammatory cell; 1 mf
		465	Vacuolation; hepatocyte; 2 d. Infiltrate, inflammatory cell; 1 mf

6) Spleen

Morphologic findings within the spleen were restricted to minimal findings of vacuolated macrophages/histiocytes within the red pulp, extending to the white pulp at up to slight severity, at the 12-16 month time-point, amongst MPSII mice only.

Table S6: Spleen- Pathologist's Comments

Group	Study	Mouse ID	Pathologist's Comments
Wild-type	Medium Term (6 months)	238	-
		239	Increased cellularity, germinal centers; 1 mf
		240	Increased cellularity, germinal centers; 2 mf
		265	-
		266	Increased cellularity, germinal centers; 1 mf
		267	Increased cellularity, germinal centers; 1 mf
		286	NA
	Long Term (10-12 months)	257	-
		261	-
		262	-
		271	-
		313	-
		328	-
		329	-
MPSII	Medium Term (6 months)	270	Infiltrate, vacuolated macrophages, red pulp; 1 mf
		278	Infiltrate, vacuolated macrophages, red pulp; 1 mf
		327	Infiltrate, vacuolated macrophages, red pulp; 1 mf. Increased cellularity, germinal centers; 1 mf
		332	Infiltrate, vacuolated macrophages, red pulp; 1 mf. Increased cellularity, germinal centers; 1 mf
		333	Infiltrate, vacuolated macrophages, red pulp; 1 mf
		334	Infiltrate, vacuolated macrophages, red pulp; 1 mf
	Long Term (10-12 months)	256	Infiltrate, vacuolated macrophages, red/white pulp; 2 mf
		259	Infiltrate, vacuolated macrophages, red/white pulp; 2 mf
		260	Infiltrate, vacuolated macrophages, red/white pulp; 2 mf
		346	Infiltrate, vacuolated macrophages, red/white pulp; 2 mf
		348	Infiltrate, vacuolated macrophages, red pulp; 1 mf
WT-HSCT	Medium Term (6 months)	285	Increased cellularity, germinal centers; 1 mf
		287	Increased cellularity, germinal centers; 1 mf
		317	-
		320	Increased cellularity, germinal centers; 1 mf
		321	-
		323	-
	Long Term (10-12 months)	301	-
		302	-
		309	-
		310	-
		314	-
		315	-
		316	-
		421	-
		422	-
LV.IDS	Medium Term (6 months)	324	NA
		325	NA
		361	Increased cellularity, germinal centers; 1 mf
		362	Increased cellularity, germinal centers; 1 mf
		363	Increased cellularity, germinal centers; 1 mf
		364	-
	Long Term (10-12 months)	355	-
		341	-
		342	-
		350	-
		357	NA
		386	NA
LV.IDS.ApoEII		397	-

Group	Study	Mouse ID	Pathologist's Comments
	Medium Term (6 months)	399	Increased cellularity, germinal centers; 1 mf
		406	-
		407	Increased cellularity, germinal centers; 1 mf
		408	-
		409	-
	Long Term (10-12 months)	4060	-
		424	-
		425	-
		426	-
		452	-
		454	-
		465	-

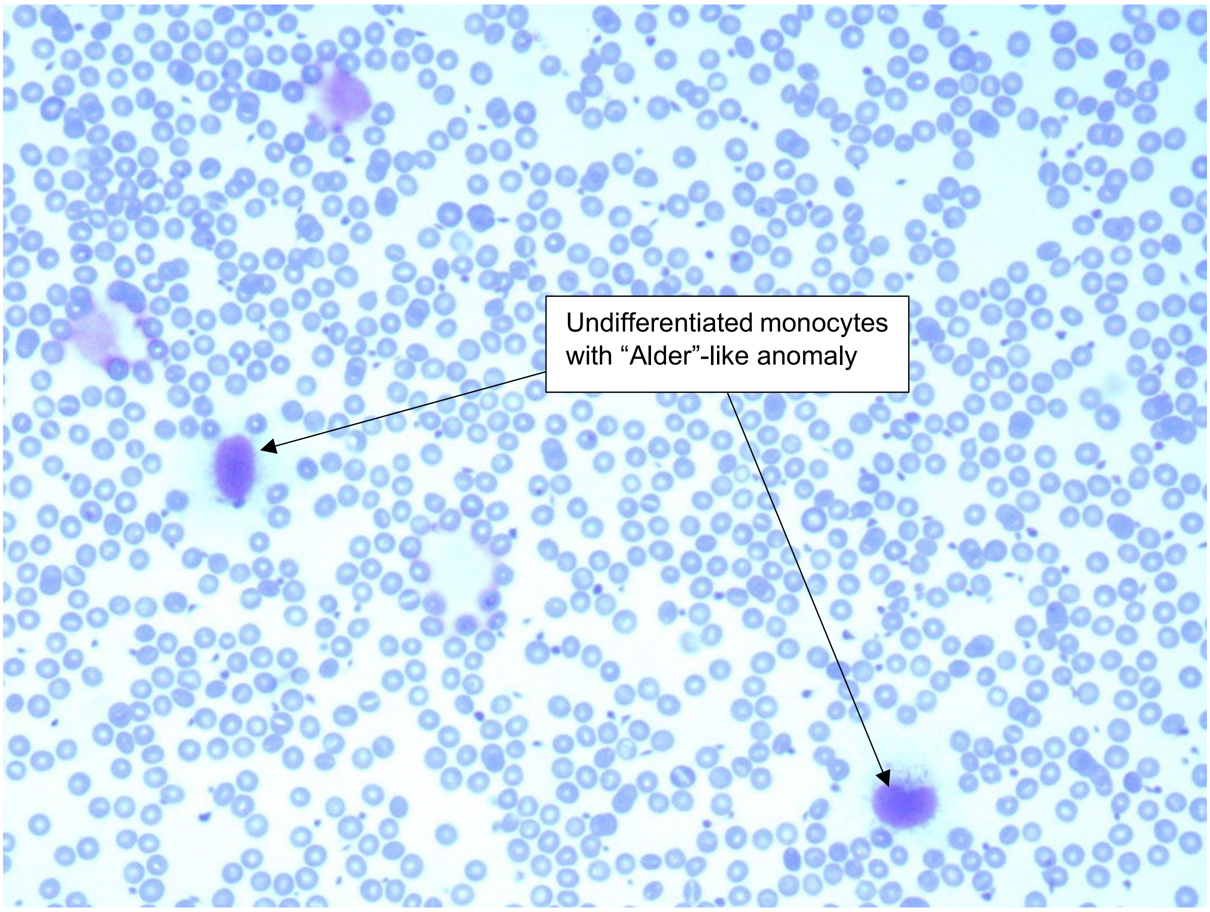

Figure S1: Specimen 334 (MPSII Group, 6 months); blood smear (40x Objective).
