# Supplementary material for: Sustained long-term disease correction in a murine model of MPSII following stem cell gene therapy
Source: Mol Ther Methods Clin Dev. 2023 Oct 20;31:101127. doi: 10.1016/j.omtm.2023.101127 (PMC10618237; doi:10.1016/j.omtm.2023.101127)
Supplement: Document S2. Article plus supplemental information [file mmc2.pdf]

Molecular Therapy
Methods & Clinical Development
Original Article

Sustained long-term disease correction in a murine model of MPSII following stem cell gene therapy

Stuart Ellison,[1,2] Aiyin Liao,[1,2] Hélène F.E. Gleitz,[1] Helen Parker,[1] Laura Booth,[1] John Robinson,[1] Shaun Wood,[1] Jessica Taylor,[1] Rebecca Holley,[1] and Brian W. Bigger[1]

[1]Stem Cell & Neurotherapies Group, University of Manchester, Manchester M13 9PT, UK

Mucopolysaccharidosis type II (MPSII) is a pediatric lysosomal storage disease caused by deficiencies in the IDS (iduronate-2-sulfatase) gene resulting in accumulation of glycosaminoglycans, multisystem disease, and profound neurodegeneration in severe forms. Although enzyme replacement therapy is available for somatic forms of disease, the inability of native IDS to pass the blood-brain barrier renders it ineffective for the brain. We previously demonstrated the short-term efficacy of a brain-targeted hematopoietic stem cell gene therapy approach to treat MPSII mice using lentiviral IDS fused to the blood-brain-barrier-crossing peptide ApoEII (IDS.ApoEII) in comparison with a lentivirus expressing native IDS and an unmanipulated bone marrow transplant. Here we evaluated the longevity of disease correction for 12–16 months following treatment. We observed sustained IDS enzyme activity in organs of long-term IDS.ApoEII-treated MPSII mice, similar to those analyzed 6 months post-treatment, with continued clearance of storage material in the brain and peripheral organs, maintained correction of astrogliosis, microgliosis, and correction of altered cytokines and chemokines. IDS.ApoEII also significantly reduced retinal atrophy, characteristic of MPSII. Overall, IDS.ApoEII resulted in systemic prevention of the MPSII phenotype, with no observed toxicity following treatment. This provides evidence of the sustained efficacy and safety of this treatment ahead of a recently opened clinical trial.

INTRODUCTION

Mucopolysaccharidosis type II (MPSII) is a rare pediatric lysosomal storage disorder (LSD) caused by iduronate-2-sulfatase (IDS) enzyme deficiency due to mutations in the *IDS* gene (GenBank: NM_000202). MPSII is X linked and therefore typically affects males, with a reported incidence of approximately 1.3 per 100,000 live births.[1] The absence of functional IDS severely disrupts the normal degradation of both heparan sulfate (HS) and dermatan sulfate (DS), leading to their accumulation in all cells throughout the body. MPSII is a multisystemic disease with a range of characteristic symptoms, including skeletal abnormalities (dysostosis multiplex), joint stiffness and pain, short stature, cardiorespiratory disease, and hepatosplenomegaly.[2,3] The most severe form of MPSII, characterized by progressive neurodegeneration and premature death, affects two-thirds of patients, with the majority not surviving into adulthood.[3,4]

Somatic symptoms in MPSII patients are generally well managed by intravenous delivery of replacement recombinant enzyme (Elaprase).[5] Enzyme replacement therapy (ERT) is possible because circulating enzyme can enter affected cells via the M6P receptor and degrade HS and DS storage. Unfortunately, ERT is not effective at treating the CNS, as it is unable to cross the blood-brain barrier (BBB), although approaches are being investigated to remedy this.[6,7] In addition, neutralizing antibodies and anaphylactic reactions are not uncommon following ERT, which can diminish the effectiveness of treatment.[8]

Historically, allogeneic hematopoietic stem cell transplantation (HSCT) has been the standard of care treatment of the similar LSD mucopolysaccharidosis type I (MPSI) (Hurler syndrome), with the ability to alleviate neurological symptoms and improve peripheral disease.[9] However, although the treatment is usually successful, skeletal abnormalities, cardiac pathology, and occasionally CNS manifestations may be only partially improved after long-term follow-up.[10] MPSII patients who undergo HSCT typically show poorer and more unpredictable clinical outcomes compared with MPSI patients, and the majority continue along the disease spectrum often with poor clinical correction, although a small percentage of patients do experience some improvements to the neurological indication.[11]

In a murine model of MPSII, we previously showed the efficacy of hematopoietic stem cell gene therapy (HSCGT) by using a lentiviral vector (LV)-based approach. This involved delivering a therapeutic transgene containing a codon-optimized IDS fused with a tandem repeat of the receptor binding domain of apolipoprotein E (ApoEII), which was driven by a myeloid-specific CD11b promoter in hematopoietic stem cells (HSCs).[12,13] The rationale for inclusion

Received 18 July 2023; accepted 4 October 2023;
https://doi.org/10.1016/j.omtm.2023.101127.

[2]These authors contributed equally

Correspondence: Brian W. Bigger, Stem Cell & Neurotherapies Group, University of Manchester, Manchester M13 9PT, UK.
E-mail: brian.bigger@manchester.ac.uk

This is an open access article under the CC BY license (http://creativecommons.org/licenses/by/4.0/).

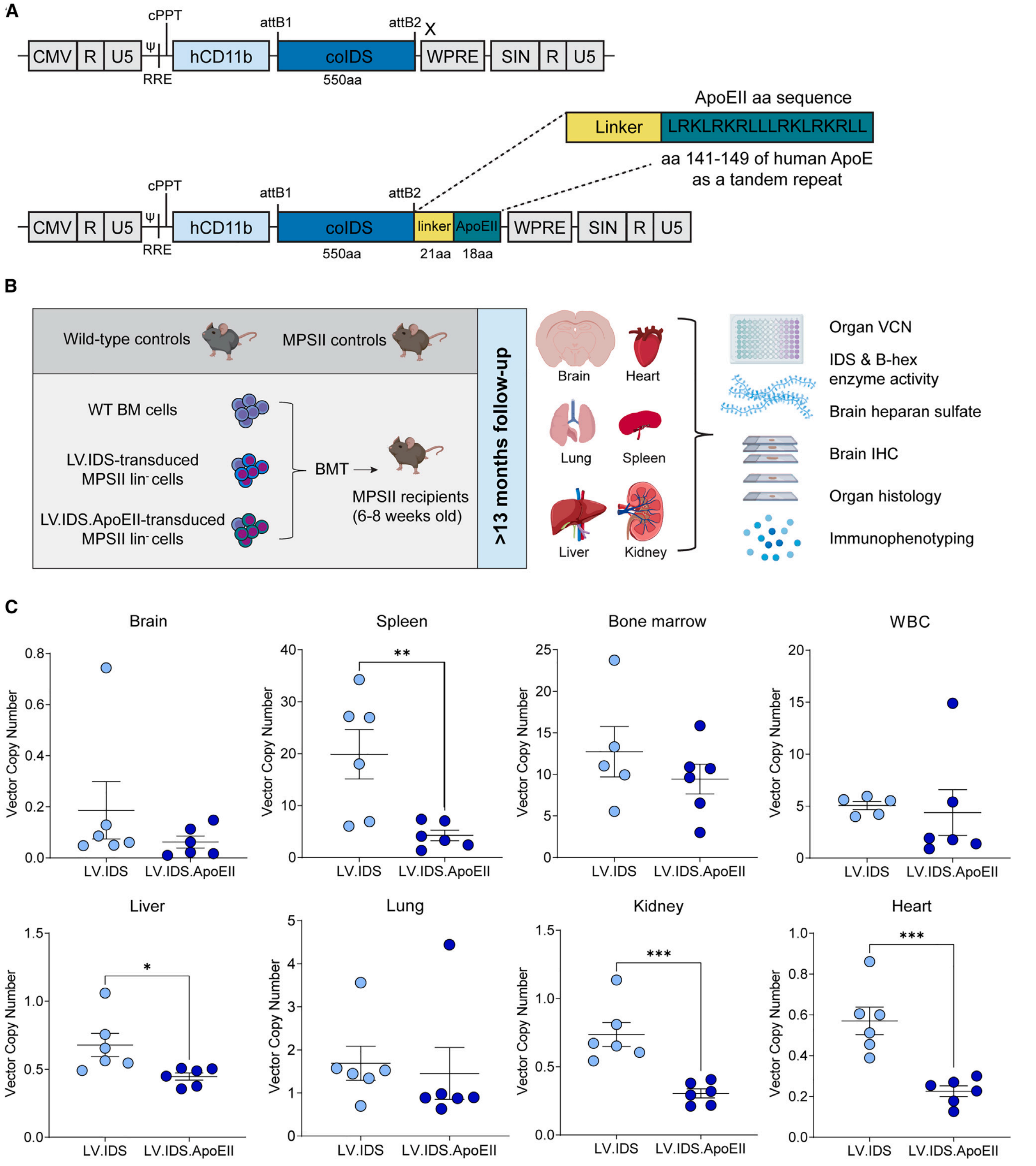

Figure 1. Lentiviral vector design and long-term study overview

(A) pCCL lentiviral vectors containing the CD11b promoter encoding codon-optimized human IDS gene or the human IDS gene followed by a flexible linker and the ApoEII peptide sequence tandem repeat. (B) The long-term HSCGT strategy. Busulfan-conditioned 6- to 8-week-old MPSII mice were transplanted with 2–3 $\times$ 10^5 lineage-depleted (Lin^-) HSCs transduced with LV.IDS or LV.IDS.ApoEII or 1 $\times$ 10^7 total bone marrow cells for the WT-HSCT group. Untreated WT and MPSII mice were included as controls.

(legend continued on next page)

of the ApoEII peptide was to further enhance CNS-targeted delivery of IDS enzyme from gene-modified cells in the periphery via receptor-mediated transcytosis across brain endothelial cells. The addition of the ApoEII peptide resulted in a more effective treatment (LV.IDS.ApoEII), completely normalizing brain pathology and behavior 6 months post-treatment, providing significantly enhanced correction compared with unmodified IDS (LV.IDS). Moreover, LV.IDS.ApoEII exhibited higher activity levels in the plasma and demonstrated a 5-fold increase in uptake in an *in vitro* model of brain endothelial cells. It was also found to undergo transcytosis through both ApoE-dependent receptors and M6P receptors.[12]

Here, we present findings from a long-term follow up study to determine whether efficacy and safety of HSCGT treatment is maintained throughout the lifetime of treated MPSII mice. The IDS and IDS.ApoEII treatment groups maintained supraphysiological levels of IDS activity in somatic organs, and IDS activity in the brain was sufficient to clear HS storage, correct sulfation patterns, and provide lasting reductions in astrogliosis, lysosome swelling, and microgliosis. Histological analysis of tissues from animals treated with LV.IDS and LV.IDS.ApoEII confirmed sustained efficacy beyond 12 months post-treatment, with no indication of toxicity except for effects associated with busulfan. Furthermore, both LV.IDS and LV.IDS.ApoEII treatments were able to rectify altered chemokine and cytokine profiles in MPSII, with LV.IDS.ApoEII demonstrating superior overall efficacy. These findings provide additional safety and efficacy data to support our recently opened phase I/II clinical trial in MPSII patients (NCT05665166).

RESULTS

MPSII HSCGT study design to assess long-term efficacy and safety

The aim of this study was to assess the long-term effectiveness of *ex vivo* HSCGT as a treatment for MPSII. We previously designed 2 LVs to express either IDS alone or IDS fused to the BBB-targeting ApoEII peptide (Figure 1A).[12] Lineage-depleted MPSII HSCs were transduced with either LV.IDS or LV.IDS.ApoEII and transplanted into 16 busulfan-conditioned 6- to 8-week-old MPSII recipient mice (Figure 1B). As a control, wild-type (WT) HSCs were transplanted into MPSII mice to mimic an allogeneic bone marrow (BM) transplant (WT-HSCT). These cohorts and the methodology for transplantation were identical to our short-term study analyzed at 8 months of age and were performed at the same time.[12] These mice formed half of the behavioral cohort analyzed, and fully presented, by Gleitz et al.[12] Mice were culled at their humane endpoint between 14 and 18 months of age, via perfusion with PBS, and 9 organs taken for subsequent analysis.

The number of integrated vector copies (vector copy number [VCN]) was assessed in the brain, spleen, BM, liver, heart, lung, kidney, and white blood cells [WBCs] of LV.IDS- and LV.IDS.ApoEII-treated mice (Figure 1C). Overall, VCN levels were higher in the LV.IDS-treated animals compared with LV.IDS.ApoEII, with a significantly higher VCN in the spleen (19.9 vs. 4.3), liver (0.68 vs. 0.45), heart (0.57 vs. 0.22), and kidney (0.74 and 0.3) and a non-significant trend of higher VCN in the brain (0.19 vs. 0.06), BM (12.72 vs. 9.43), lung (1.69 vs. 1.45), and WBCs (5.05 vs. 4.38) between the IDS and IDS.ApoEII groups, respectively. Lower VCNs in the LV.IDS.ApoEII group could be a consequence of either engraftment of HSCs with lower transduction after transplantation, as there was a very wide spread of pre-transplantation transduction efficiency of IDS.ApoEII seen in Figure 1 in Gleitz et al.,[12] or possibly lower engraftment potential in peripheral organs by IDS.ApoEII.

Supraphysiological IDS enzyme activity levels are sustained in long-term LV.IDS and LV.IDS.ApoEII mice

We measured iduronate sulfatase and β-hexosaminidase (β-hex) activity levels in long-term-treated MPSII mice (Figure 2) and observed supraphysiological levels of IDS activity in most organs from the IDS and IDS.ApoEII treatment groups (Figure 2A), with particularly high levels recorded in the spleen (1,984% and 1,244% of WT levels, respectively), and hematopoietic system (BM, 5,068% and 1,006%; WBCs, 2,450% and 2,930%). Very high IDS activity was also detected in the liver (1,134% and 524% of WT), heart (1,100% and 370%), and lung (277% and 156%) in the IDS and IDS.ApoEII transplantation groups, respectively (Figure 2A). In the kidney, the IDS treatment group provided approximately 3.5-fold WT IDS levels where the IDS.ApoEII treatment IDS activity was comparable with WT. In the brain, we observed 14% and 4% of WT IDS activity levels in the IDS and IDS.ApoEII treatment groups, respectively (Figure 2A). Overall higher levels of IDS activity were recorded in the IDS only treatment group, in line with the VCN data (Figure 1C). Elevated endogenous lysosomal enzyme β-hex levels are typical of MPS disorders and believed to rise as a consequence of lysosomal enzyme deficiency.[14] As expected, we observed significantly elevated β-hex levels in the brain, liver, heart, lung, and kidney of untreated MPSII mice (Figure 2B). Both IDS and IDS.ApoEII treatments were able to correct β-hex activity to WT levels in the liver, heart, lung, and kidney more effectively than the WT-HSCT cohort. In the brain, both IDS and IDS.ApoEII treatments significantly reduced β-hex activity in MPSII mice by 38.6% and 41.6%, respectively, improving upon the 19.3% reduction seen following WT-HSCT.

HS storage in brain and liver is corrected with LV.IDS and LV.IDS.ApoEII

HS accumulation in the brain is believed to be a major contributing factor to neurocognitive decline in MPS patients.[15] In addition, the altered sulfation patterning is also a trigger for neuroinflammatory events that accelerate disease progression.[16] Both IDS and IDS.ApoEII were able to significantly reduce MPSII brain HS levels

Six animals per group were sacrificed at ≥13 months of age for biochemical and microscopic analysis including qPCR for vector copy number (VCN), IDS enzyme activity, β-hex activity, brain heparan sulfate, brain immunohistochemistry, immunophenotyping, and histology of organs. (C) VCN analysis in brain, spleen, bone marrow, liver, heart, lung, kidney, and white blood cells (WBCs). Data are mean ± SEM. Student's t-test: $^{**}p < 0.01$, $^{***}p < 0.001$.

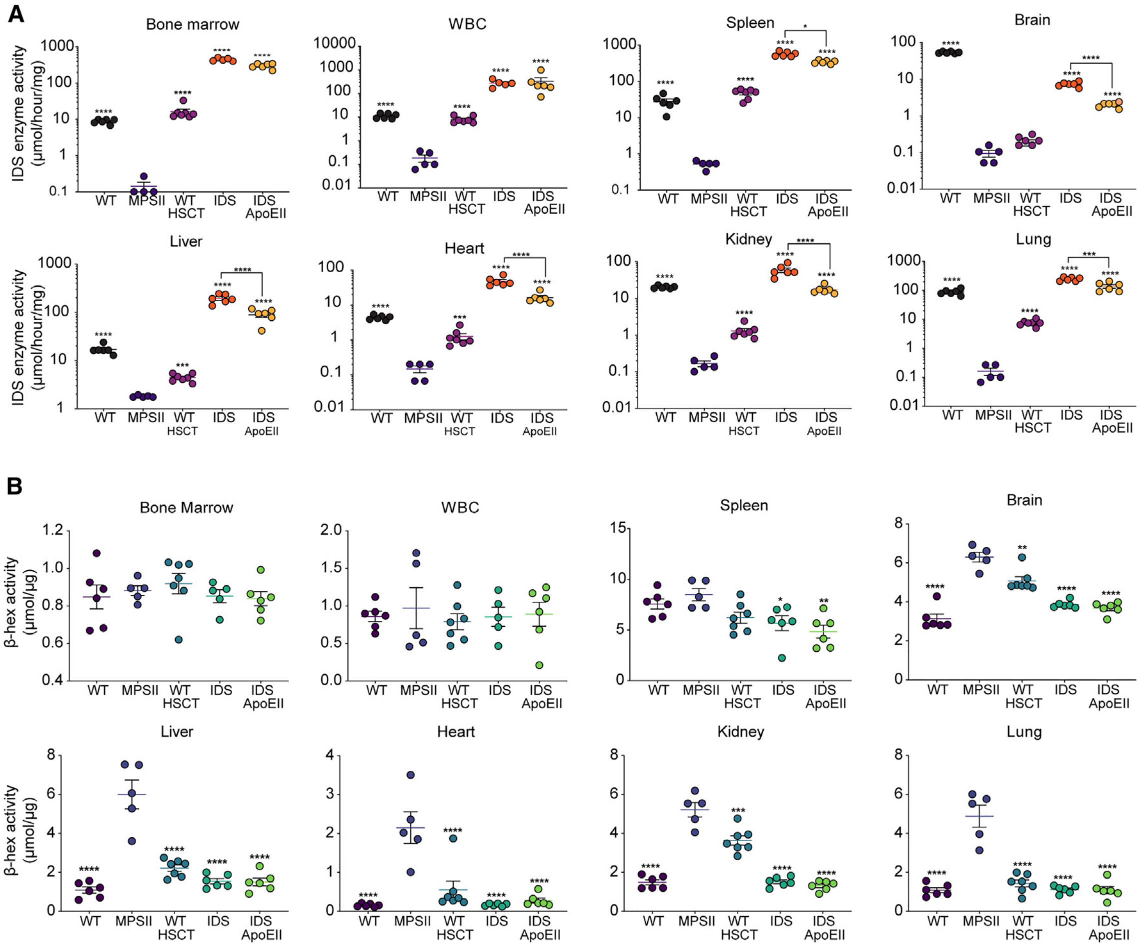

Figure 2. LV.IDS and LV.IDS.ApoEII provide sustained brain-specific IDS activity and supra-physiological levels of active IDS in peripheral organs

(A) IDS enzyme activity levels measured in organs taken at ≤13 months of age, including brain, spleen, bone marrow, liver, heart, lung, kidney, and WBCs from control and treated mice (n = 6–10 mice/group). Black dots, WT; purple dots, MPSII; pink dots, WT-HSCT; orange dots, IDS; yellow dots, IDS ApoEII. (B) Levels of lysosomal enzyme β-hexosaminidase activity in brain, spleen, bone marrow, liver, heart, lung, kidney, and WBCs of ≤13-month-old mice (n = 6–10 mice/group). Purple dots, WT; blue dots, MPSII; turquoise dots, WT-HSCT; green dots, IDS; light green dots, IDS ApoEII. All data are mean ± SEM. One-way ANOVA: ns, $p > 0.05$; *$p < 0.05$, **$p < 0.01$, ***$p < 0.001$, and ****$p < 0.0001$ vs. MPSII; other comparisons are indicated by brackets. For IDS enzyme activity, log-transformed data were used for statistical analysis.

by 4.3- and 2.8-fold, respectively, and normalize the HS sulfation patterning in comparison with WT-HSCT, where total HS levels were not reduced in treated MPSII mice, and only a partial change in HS sulfation patterning was observed (Figures 3A and 3B). In the liver, all treatment groups were able to restore HS to WT levels, but only IDS and IDS.ApoEII treatments were able to normalize HS sulfation patterning (Figures 3C and 3D).

Sustained reductions in astrogliosis, lysosome swelling, and microgliosis in LV.IDS- and LV.IDS.ApoEII-treated MPSII mice

Astrogliosis and lysosomal swelling are hallmark features of MPSII and can be observed throughout various regions of the brain, most notably in the cortex, striatum, hippocampus, and amygdala (Figure 4A). Immunohistochemical staining using a glial fibrillary associated protein (GFAP) stain for reactive astrocytes (green) demonstrated significantly increased astrogliosis in untreated MPSII mice compared with WT in all brain regions tested. In the cortex and hippocampus, LV.IDS and LV.IDS.ApoEII significantly reduced MPSII associated astrogliosis by similar levels (Figures 4A, 4B, and 4D). In the striatum and amygdala, LV.IDS.ApoEII was the most effective treatment for astrogliosis reduction and was more significantly reduced than LV.IDS with almost complete normalization (Figures 4C and 4E). Co-staining of the same coronal sections with the lysosome marker LAMP2, and sections co-stained with the neuronal markers NeuN and LAMP2, indicated a substantial reduction of swollen lysosomes in the IDS and IDS.ApoEII treatment groups to near normal levels in both astrocytes (Figure 4A) and neurons (Figure 5) in the cortex and hippocampus, but only for IDS.ApoEII in the amygdala, compared with the WT-HSCT group, in which there appeared to be little to no correction of lysosomal

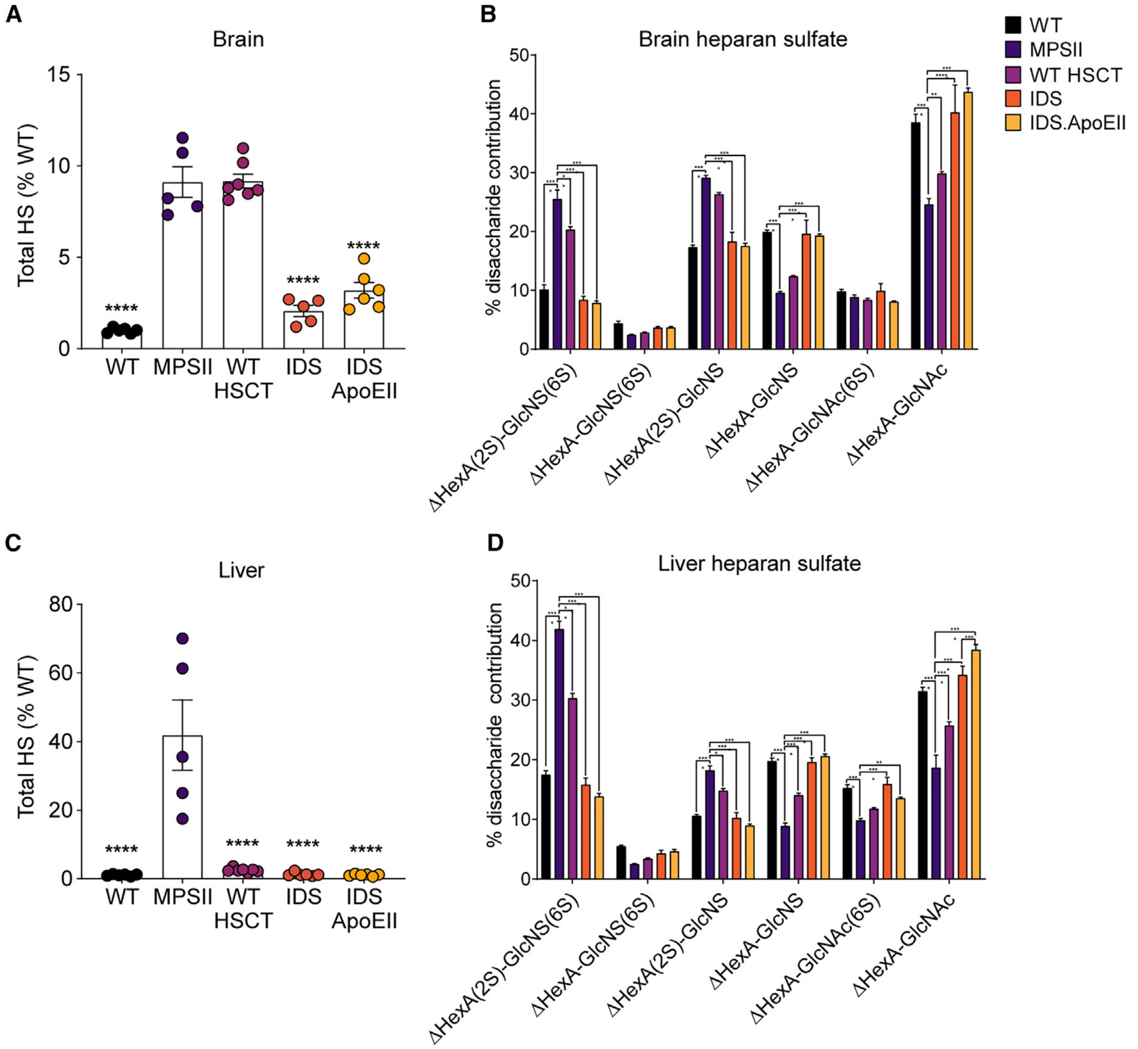

Figure 3. LV.IDS and LV.IDS.ApoEII significantly reduce brain HS accumulation and normalize HS sulfation patterning

Total relative amounts of HS and compositional disaccharide analysis of HS from control and treated mice brain (A and B) and liver (C and D) samples (n = 6–10 mice/group). Black, WT; purple, MPSII; pink, WT-HSCT; orange, IDS; yellow, IDS ApoEII group. All data are mean ± SEM. One-way ANOVA: ns, $p > 0.05$; *$p < 0.05$, **$p < 0.01$, ***$p < 0.001$, and ****$p < 0.0001$ vs MPSII.

swelling. Interestingly, lysosomal swelling in the striatum was less effectively corrected with both IDS and IDS.ApoEII treatment groups compared with other brain regions (Figures 5A and 5C). In untreated MPSII mice, we observed increases of 67-, 118-, 82.6-, and 79.3-fold of isolectin B4 (ILB4)-positive activated microglial cells in the cortex, striatum, hippocampus, and amygdala, respectively (Figure 6). In the four brain regions evaluated, IDS.ApoEII was the most effective at normalizing the number of activated microglia with significant reduction observed in the cortex and amygdala compared with LV.IDS (Figures 6B and 6C), and this was also the most effective treatment at reducing lysosomal storage in neurons (Figure 5A). Overall, these findings suggest that the characteristic astrogliosis and microglial activation in the brains of MPSII mice can be prevented over a sustained period following treatment with LV.IDS and LV.IDS.ApoEII, with LV.IDS.ApoEII being the most effective, despite four times lower IDS enzyme activity levels than LV.IDS.

LV.IDS.ApoEII treatment corrects MPSII associated eye abnormalities

Within the scope of pre-clinical safety and efficacy investigations, fixed tissue samples from each experimental group were submitted to an external organization, Histologix, to conduct H&E evaluations under good clinical laboratory practice (GCLP)-like conditions, to identify any potential organ toxicity alongside biodistribution. These studies were designed to (1) assess the efficacy of treatment on the MPSII phenotype; (2) evaluate any toxicological effects following WT-HSCT, LV.IDS, or LV.IDS.ApoEII HSCGT; and (3) assess any

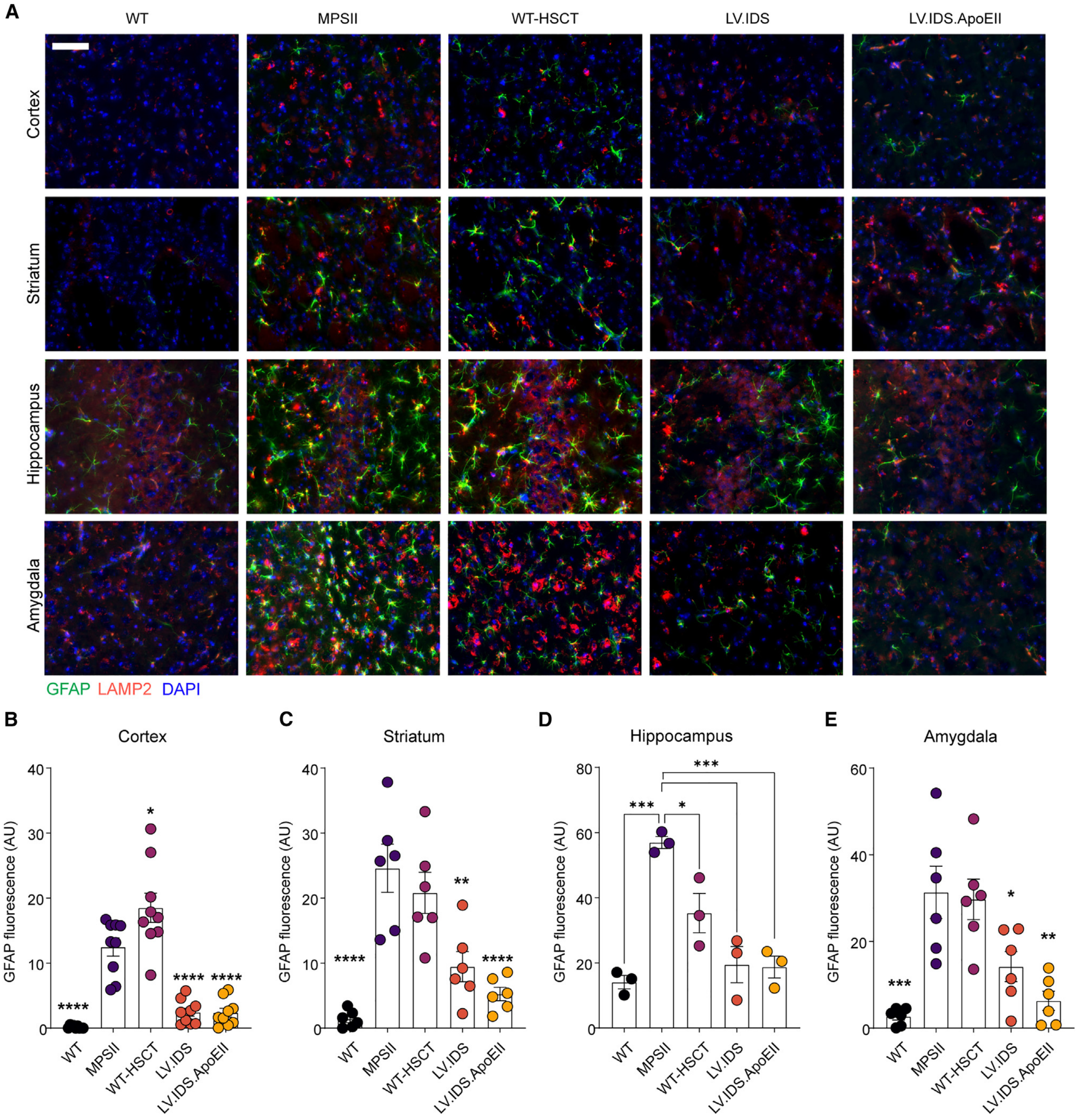

Figure 4. LV.IDS.ApoEII normalizes astrogliosis in the brains of ≤13-month-old MPSII mice

(A) Representative images of 30 μm brain sections of the motor cortex (M2), striatum (both −0.46 mm from bregma), hippocampus (CA3), and amygdala (both −1.22 mm from bregma) from control and treated mice stained with GFAP (green) and LAMP2 (red) (n = 6 mice/group), 40×; nonlinear adjustments were made equally to reduce background: gamma 0.72 and input levels of 0–190. Scale bar: 50 μm. GFAP immunofluorescence was quantified in the cortex (B), striatum (C), hippocampus (D), and amygdala (E) of ≤13-month-old MPSII mice (n = 6 mice/group). Black dots, WT; purple dots, MPSII; pink dots, WT-HSCT; orange dots, IDS; yellow dots, IDS ApoEII. AU, arbitrary units. Data are mean ± SEM. One-way ANOVA: *p < 0.05, **p <0.01, ***p < 0.001, and ****p < 0.0001 vs. MPSII.

toxicological effects of busulfan co-administration. As expected, findings associated with the intra-cellular accumulation of GAG (cytoplasmic vacuolation), along with secondary atrophic, inflammatory, and degenerative findings were most pronounced in the MPSII control group (Figure S1). There was an attenuation of these findings with WT-HSCT administration, most notably in the eye, heart, kidney, liver, and

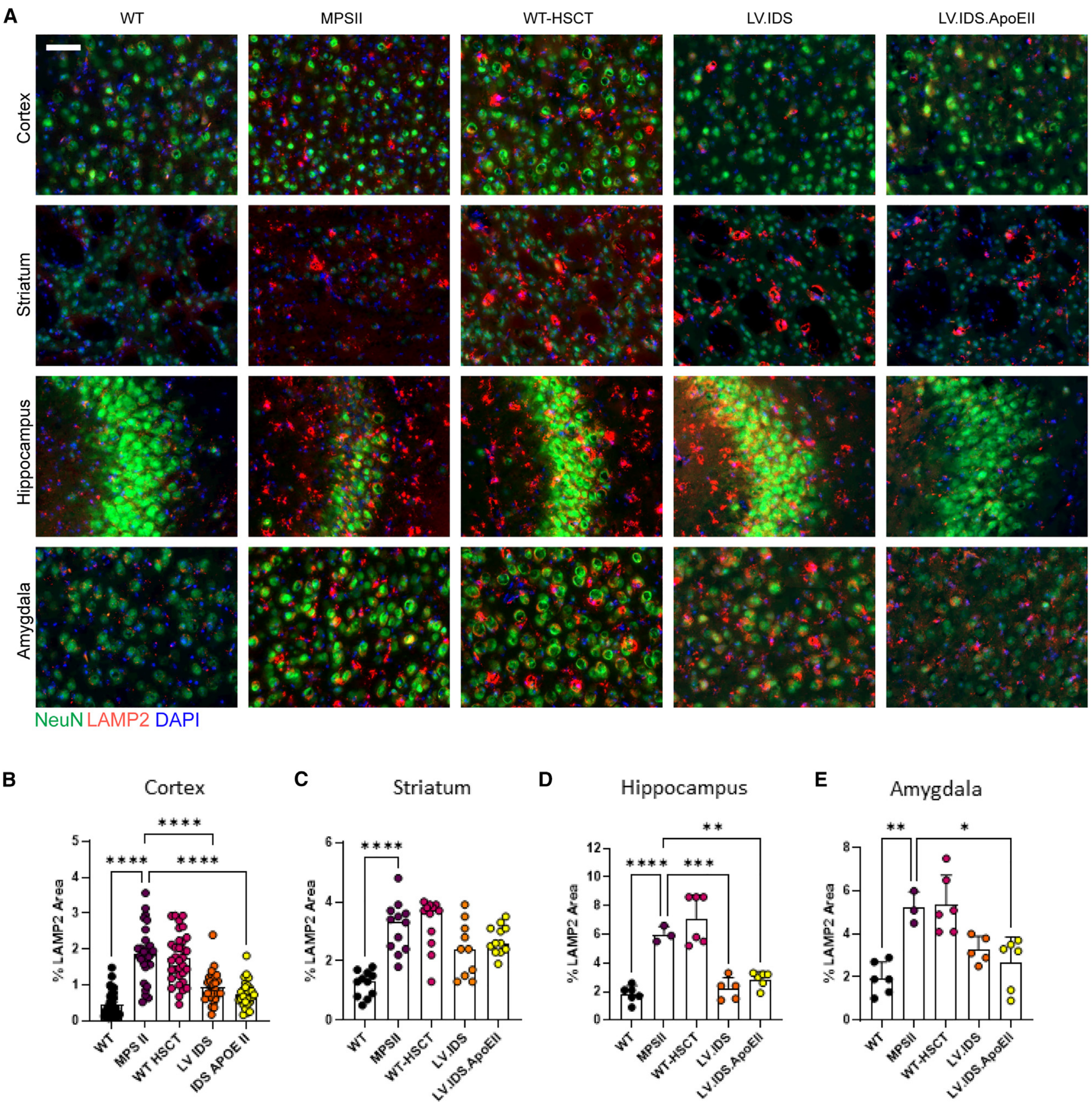

Figure 5. LV.IDS.ApoEII reduces lysosomal swelling and neuronal cell shrinkage in the brains of ≤13-month-old MPSII mice

(A) Representative images of 30 μm brain sections of the motor cortex (M2), striatum (both −0.46 mm from bregma), hippocampus (CA3), and amygdala (both −1.22 mm from bregma) from control and treated mice stained with NeuN (green) and LAMP2 (red) (n = 6–10 mice/group). Quantification of percentage area of LAMP2 staining in the cortex (B), striatum (C), hippocampus (D), and amygdala (E). Black dots, WT; purple dots, MPSII; pink dots, WT-HSCT; orange dots, IDS; yellow dots, IDS ApoEII. Data are mean ± SEM. One-way ANOVA: *p < 0.05, **p <0.01, ***p < 0.001, and ****p < 0.0001 vs. MPSII.

spleen (Figure S1). This effect was mirrored with both LV.IDS and LV.IDS.ApoEII administration, and therefore indicative of favorable efficacy. Of particular note was the profound reduction in the severity of retinal atrophy among the LV.IDS.ApoEII treatment group (Figure 7). Similarly, in the kidney and liver, LV.IDS.ApoEII treatment resulted in minimal tubular vacuolation and minimal hepatocellular vacuolation compared with untreated animals (Figure 7). Overall, the administration of LV.IDS or LV.IDS.ApoEII offered multisystem efficacy in the attenuation of the severity of the MPSII phenotype. There was no indication of toxicity following treatment using either vector,

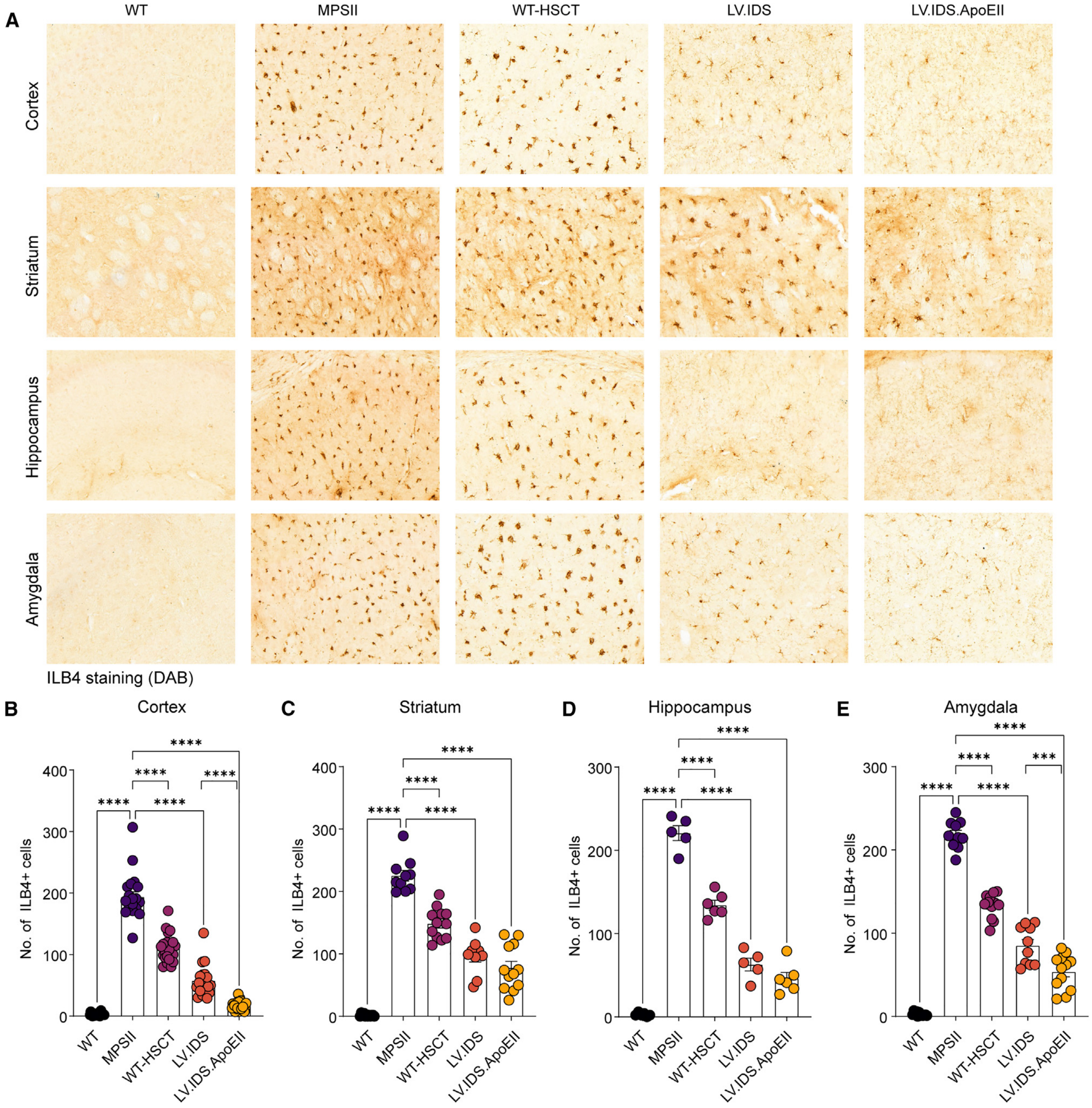

Figure 6. LV.IDS.ApoEII normalizes microgliosis in the brains of ≤13-month-old MPSII mice

(A) Representative images of 30 μm brain sections of the motor cortex (M2), striatum (both −0.46 mm from bregma), hippocampus (CA3), and amygdala (both −1.22 mm from bregma) from control and treated mice stained with isolectin B4 (ILB4) to identify activated microglia, 40×. Scale bar: 50 μm. (B) Four 30 μm sections per mouse of the cortex (B), striatum (C), hippocampus (D), and amygdala (E) were counted for the number of ILB4-positive cells (0.26 to −1.94 mm from bregma), n = 6–10 mice/group. Black dots, WT; purple dots, MPSII; pink dots, WT-HSCT; orange dots, IDS; yellow dots, IDS ApoEII. Data are mean ± SEM. One-way ANOVA: *p < 0.05, **p <0.01, ***p < 0.001, and ****p < 0.0001 vs. MPSII.

apart from some expected toxicological effects observed as a result of busulfan co-administration, especially in kidney and lung (Figure S1). No neoplasms or lymphomas or evidence of malignancy were reported in treated or untreated mice.

LV.IDS.ApoEII treatment reverses the altered cytokine and chemokine profiles of MPSII mice

Among the numerous clinical manifestations described for MPS, chronic immune dysregulation has been consistently reported.[16–18]

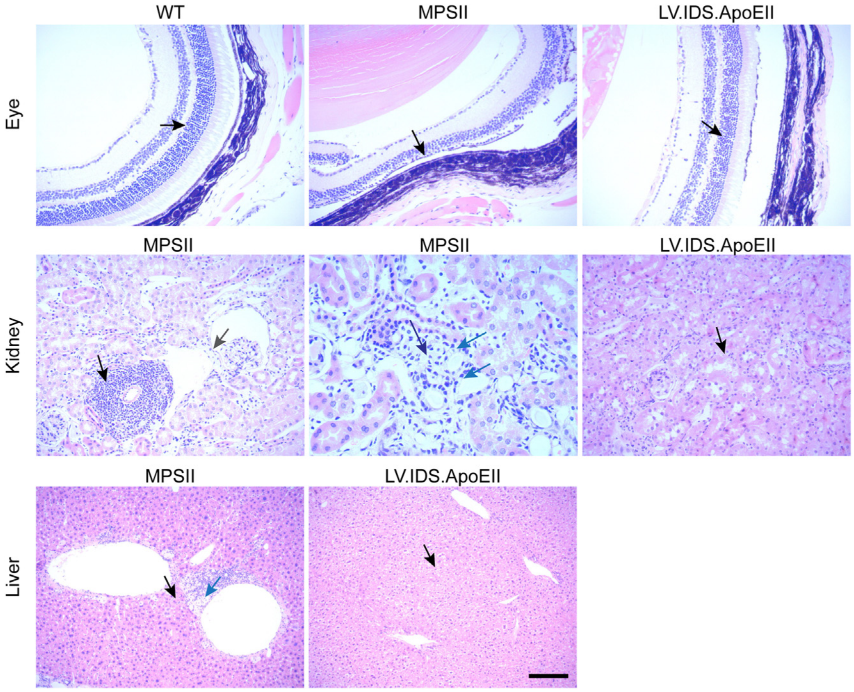

Figure 7. H&E staining of the eye, kidney, and liver to evaluate efficacious effect of treatment upon the MPSII phenotype and potential toxicological effects

Sections were analyzed from control and treatment groups by light microscopy and initially blinded (n = 7 mice/group). For the eye, black arrows highlight the near total loss of outer nuclear rods and cones observed in MPSII mice compared with the minimal loss observed in IDS.ApoEII-treated mice, similar to WT. In MPSII kidneys, the black arrow indicates regions of peri-vascular inflammation, the gray arrow highlights peri-glomerular vacuolation, the dark blue arrow indicates vacuolated macrophages with the interstitium, and the light blue arrows highlight vacuolated tubular epithelium. Minimal tubular vacuolation is observed in the kidneys of IDS.ApoEII-treated MPSII mice (black arrow). In MPSII liver, regions of peri-vascular inflammation (black arrow) and expansion of peri-venous space by vacuolated macrophages (blue arrow) are indicated. In livers from IDS.ApoEII-treated MPSII mice, minimal hepatocellular vacuolation was observed.

The molecular mechanisms involved in the inflammatory response however are still not fully understood, but inflammasome activation has been described in MPSII, which is usually associated with elevated IL-1β. We used a Bio-Plex multiplex immunoassay system to identify significantly dysregulated cytokine and chemokine levels in MPSII mice compared with WT mice. Furthermore, we assessed whether our WT-HSCT or HSCGT treatments could restore any observed alterations back to WT levels. Additionally, we investigated whether the inclusion of the ApoEII tag in LV.IDS.ApoEII treatment led to an unfavorable cytokine response compared with LV.IDS treatment. A 23-plex assay was used to profile the following cytokines and immune mediators: IL-1α, IL-1β, IL-2, IL-3, IL-4, IL-5, IL-6, IL-9, IL-10, IL-12 (p40), IL-12 (p70), IL-13, IL-17A, eotaxin, G-CSF, GM-CSF, IFN-γ, KC, MCP-1, MIP-1α, MIP-1β, RANTES, and TNF-α. Cytokine profiles in the brain, heart, liver, lung, spleen, and kidney were evaluated in control and treated groups (Figure 8). Only cytokines or chemokines where a significant difference was observed between MPSII and WT are presented. In the brain, MIP-1α levels were elevated in MPSII mice compared with WT. Both LV.IDS and LV.IDS.ApoEII treatments were able to significantly reduce MIP-1α levels in the brain, whereas the WT-HSCT did not (Figure 8A). MIP-1α was also elevated in the MPSII heart, which was normalized back to WT levels in all treatment groups, including WT-HSCT. In the liver, MIP-1α and MIP-1β were significantly elevated in MPSII and IL-9 levels reduced (Figure 8C). All treatment groups were able to reduce MIP-1α levels, whereas only IDS.ApoEII was able to normalize MIP-1β. All 3 treatment groups were able to significantly improve IL-9 levels above MPSII (Figure 8C). In the lung, MIP-1α, MIP-1β, and IL-1β were elevated in MPSII. MIP-1α and MIP-1β were normalized in all treatment groups, while IL-1β levels were normalized with IDS and IDS.ApoEII treatment (Figure 8D). In the spleen and kidney, there were significant changes in 8 different cytokines and chemokines identified in MPSII mice compared with WT (Figures 8E and 8F). In the spleen, the elevated levels of MPI-1α, MIP-1α, IL-1β, TNF-α, and IL-12 (p40) observed in MPSII were all corrected in WT-HSCT, IDS, and IDS.ApoEII treatment groups (Figure 8E). However, only IDS.ApoEII treatment significantly reduced IL-1α levels. IL-2 levels were significantly reduced in MPSII spleens compared with WT, and all 3 treatment groups were able to reverse this. G-CSF levels were elevated in the MPSII group, which were significantly reduced following treatment with IDS and IDS.ApoEII but not WT-HSCT. Interestingly, in the kidney, only eotaxin and MIP-1α saw significantly elevated levels in MPSII mice compared with significantly reduced levels of IL-1β, IL-2, IL-5, IL-10, IL-17A, and KC (Figure 8F). The elevated levels of eotaxin and MIP-1α and the reduced level of IL-2 were normalized in all 3 treatment groups. The decreased levels of IL-5, IL-10, IL-17A, and KC were significantly increased by both IDS and IDS.ApoEII but not by WT-HSCT. The decreased level of IL-1β was significantly increased by WT-HSCT and IDS treatment and elevated but not statistically significant in the IDS.ApoEII treatment group. Overall, IDS and IDS.ApoEII were similarly effective at normalizing altered cytokine and chemokine levels observed in MPSII mice. Notably, cytokine responses between LV.IDS and LV.IDS.ApoEII were consistently similar to each other, suggesting no toxic effects from the ApoEII tag.

DISCUSSION

The purpose of this study was to determine if efficacy and safety of HSCGT treatment with LV.IDS.ApoEII can be maintained over the lifetime of treated MPSII mice. In our initial proof-of-concept (PoC) investigation, which provided efficacy data 6 months

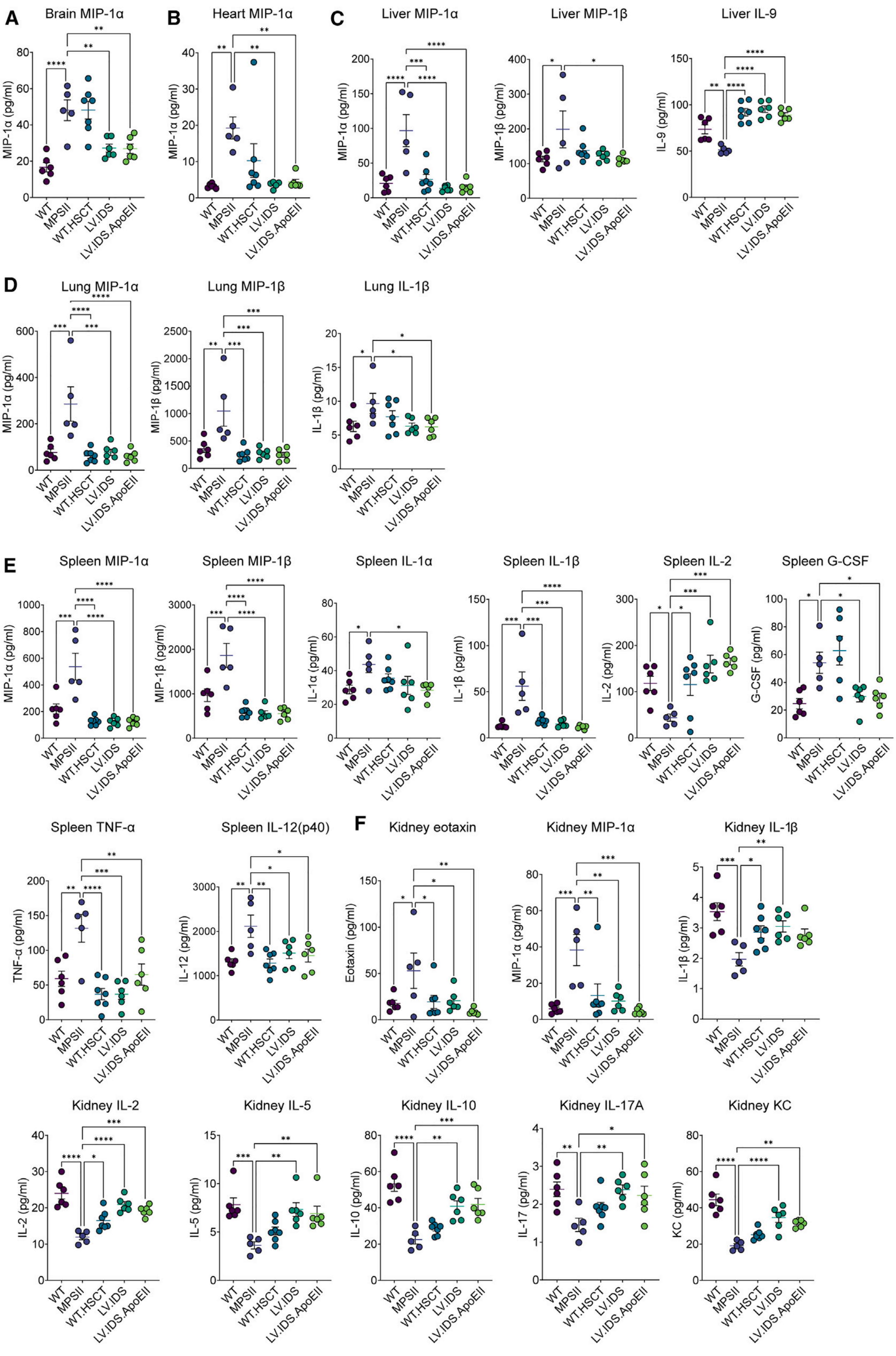

(legend on next page)

post-treatment, we observed significant improvements in working memory deficits, neuro-inflammation, and HS storage in the brain of MPSII mice treated with LV.IDS.ApoEII. Additionally, we observed normalized rotarod activity, reduced peripheral inflammation, and improvements in other somatic disease markers associated with MPSII.[12] These improvements were only partially achieved with the unmodified LV.IDS vector, suggesting that LV.IDS.ApoEII offers superior correction capabilities. For the long-term study, the mice included in this analysis constituted half of the behavioral cohort previously reported in Gleitz et al.[12] Treated mice were allowed to reach their humane endpoint, which on average occurred 12–15 months after treatment. Encouragingly, we observed similar biochemical outcomes to those observed at the six-month time point, suggesting this therapy will likely provide long-term therapeutic benefit once translated to the clinic.[12] Mice were treated at a pre-symptomatic time point to offer the greatest potential for disease correction before the onset of irreversible neurocognitive and peripheral damage. We have recently shown that MPSII mice transplanted at 4 months of age with HSGCT have worse outcomes compared with those transplanted at 2 months of age prior to symptom onset.[19] Our recently opened phase I/II clinical trial is recruiting MPSII patients aged 4–12 months, which is before neuronopathic patients begin to miss cognitive milestones (ClinicalTrials.gov identifier NCT05665166).

In the brain, we observed 14% and 4% of WT IDS activity levels in the 12–15 month time point for IDS and IDS.ApoEII treatment groups, respectively, compared with 3.4% and 3.7% at the 6 month time point.[12] The higher levels of brain IDS enzyme in the LV.IDS treatment group potentially explains why there is increased clearance of brain HS compared with mice in the LV.IDS.ApoEII group (4.3- vs. 2.8-fold reduction in MPSII brain HS storage, respectively). At the 6 month time point, the LV IDS cohort demonstrated no improvement in working memory,[12] but given the superior IDS activity levels observed here at 10–13 months post-treatment, it is plausible that behavioral outcomes (measured at 6 months by Gleitz et al.[12]) could be improved by LV IDS if evaluated at a later time point. Despite overall lower VCN and commensurate IDS enzyme activity in the LV.IDS.ApoEII treatment group, compared with LV.IDS, brain HS sulfation patterning was corrected, astrogliosis and microglial activation were completely abrogated, and lysosomes were restored to normal compartment size, similar to the observation at the 6 month post-treatment time point and equivalent to (or more effectively so), in some brain regions, especially the amygdala, which is critical for behavior. In line with other clinical findings,[20,21] brain IDS levels in our PoC study increase over time, from 3.4% to 14%, at 6 and 12–15 month time points, respectively. This suggests that continued engraftment of LV.IDS modified cells over time. In contrast, in the IDS.ApoEII group, enzyme levels remain constant at the 6 and 12–15 month time points, with 3.7% and 4% detected, respectively, which could potentially be explained by lower engraftment in this group, but a more likely explanation is a lower level of transduction in IDS.ApoEII in hematopoietic organs and similar changes in enzyme activity. The mice in each cohort were selected at random for either 6 month (Gleitz et al.[12]) or 12–15 month histological and pathological analysis (this study), so this could have been by chance.

Since our initial PoC study, other groups have also evaluated the potential of HSGCT for the treatment of MPSII. Smith et al.[22] used an MPSII mouse model to evaluate the efficacy of *ex vivo* LV gene-modified HSPCs encoding a codon-optimized human IDS under the control of a strong constitutively active MNDU3 viral-derived promoter (MNDU3-IDS). In the study of Smith et al., 13% of WT IDS levels were achieved in the brain with GAG accumulation restored, comparable with our findings with LV.IDS in our long-term evaluations. However, weaker, but more specific, mammalian promoters such as CD11b or PGK may prove to be safer than viral-derived promoters such as MND, as they may be less likely to activate downstream genes on random genome integration. The MND promoter, despite a good safety record to date, may have been implicated in the recent trial halt reported for X-ALD, following the development of myelodysplastic syndrome in a single patient, which has since re-started.[23,24]

No reports of blood cancers have been reported in other HSCGT clinical trials using either the ubiquitous PGK or CD11b promoter, as used in our vector.[20,25] We used the CD11b promoter for our vector, as it is a human specific promoter that restricts transgene expression to myeloid cells, some of which enter the brain and other organs. Together with the ApoEII BBB-crossing peptide, which helps direct enzyme distribution from the periphery toward the brain and improves bEND3 cell uptake by 5-fold, this combination of approaches, reduces the disparity between somatic enzyme activity levels and brain levels, which are typically in the order of 10- to 100-fold higher in somatic organs than the brain, and is potentially a more effective enzyme because of its improved uptake.[13,26–28]

As part of good pharmocovigilance practice, we evaluated potential toxicity of the LV.IDS.ApoEII drug product and treatment efficacy by performing histological analysis on the organs of treated and untreated MPSII and control WT mice. The study concluded that administration of either LV.IDS or LV.IDS.ApoEII treatment offered multisystem efficacy, in the attenuation of the MPSII phenotype. The adverse structural findings within the MPSII control cohort, most notably in the kidney and liver, were largely absent in tissues from WT-HSCT, LV.IDS, and LV.IDS.ApoEII treatment groups. Both the MPSII control group and the WT-HSCT treated group of mice displayed retinal atrophy, which is considered adverse, as it would likely result in significant visual impairment. However, in the LV.IDS- and LV.IDS.ApoEII-treated groups, the occurrence of retinal

Figure 8. LV.IDS.ApoEII corrects altered cytokine and chemokine profiles in MPSII mice

Brain (A), heart (B), liver (C), lung (D), spleen (E), and kidney (F) samples were evaluated in a 23-plex Bio-Plex assay. Only cytokines/chemokines profiles with significant differences between MPSII and WT are shown. Purple dots, WT; blue dots, MPSII; turquoise dots, WT-HSCT; green dots, LV.IDS; light green dots, LV.IDS.ApoEII. Data are mean ± SEM. One-way ANOVA: ns, $p > 0.05$; $^{*}p < 0.05$, $^{**}p < 0.01$, $^{***}p < 0.001$, and $^{****}p < 0.0001$ vs. MPSII.

atrophy was profoundly reduced. This suggests that the therapeutic effect of both treatments extends across the blood-eye barrier, potentially mitigating the risk for sight impairment. Lenticular cataracts were identified in treatment groups in the study but were considered a secondary effect arising from the co-administration of busulfan rather than from administration of WT-HSCT, LV.IDS, or LV.IDS.ApoEII.[29,30]

To investigate inflammatory responses in MPSII and their correction following treatment, we chose to use a multiplex system, as multiple cytokine and chemokine profiles can be evaluated at once in a single reaction for each organ. In practice, we discovered this approach lacks sensitivity compared with the CBA flex set methodology used in our previous study.[12] Despite this, we were able to identify interesting findings in the long-term-treated MPSII mice. In MPSII mice, the levels of MIP-1α, a marker of macrophage activation, were found to be elevated in the brain, heart, liver, lung, spleen, and kidney, indicating a state of heightened macrophage activity in these affected organs. However, all three treatment groups showed normalization of MIP-1α levels, except in the brain, in which WT-HSCT was ineffective compared with HSCGT using either LV.IDS or LV.IDS.ApoEII. Several studies have indicated that IL-9 is a cytokine commonly associated with inflammatory, allergic, and autoimmune diseases as well as parasitic infections and lack of IL-9 prolongs inflammation resolution in models of arthritis and ulcerative colitis.[31–34] Here, IL-9 is specifically downregulated in MPSII livers. Recently a study focused on alcoholic liver injury (ALI) showed IL-9 downregulation in a mouse model of ALI, with concomitant secretion of hepatic macrophage pro-inflammatory factors, inflammatory cell infiltration, and the appearance of a large number of hepatic vacuoles, similar to those observed in MPSII livers.[35] Our findings suggest that T cell polarization may be affected in MPSII, as indicated by decreased levels of specific cytokines in the liver. These include reduced levels of IL-9, typically produced by T helper type 9 (Th9) $CD4^+$ T cells, as well as decreased IL-2, which is involved in T cell expansion. Additionally, IL-5, associated with Th2 $CD4^+$ T cells, IL-10 produced by $Foxp3^+$ regulatory T cells (Tregs), and IL-17A/KC produced by Th17 $CD4^+$ T cells, were also found to be downregulated. T cells could potentially be becoming exhausted or may have an inability to polarize from their naive state in MPSII mice. Similarly, persistent stimulation of the immune system by viral and bacterial infection can induce T cell exhaustion, leading to loss of cytokine production and reduced immune cell function.[36,37] Treg deficiency is reported in some chronic autoimmune deficiencies, and MPSII may exhibit similar traits.[38,39]

Interestingly, WT-HSCT treatment has positive biochemical benefit in certain organs, such as the lung, heart, spleen, and liver, in the context of normalizing β-hex activity, likely a consequence of IDS activity in these organs being close to WT levels. In contrast, WT-HSCT demonstrates limited effectiveness in correcting the brain and kidney. This could potentially be attributed to relatively low enzyme levels, possibly resulting from insufficient macrophage engraftment in the kidney. On the other hand, treatment with LV.IDS and LV.IDS.ApoEII shows notable improvements, approaching levels similar to those observed in unaffected individuals (WT levels). Neuronal cross-correction is prominently observed in this study, as has been previously documented in other HSGCT studies.[12,13,27] The reduction in lysosomal compartment size toward normal levels is particularly evident in neurons of LV.IDS.ApoEII mice, as illustrated in Figure 5.

On the basis of the outcomes of this long-term PoC study and the findings obtained at the six-month time point, we effectively demonstrate the safety, efficacy, and durability of HSCGT treatment using IDS.ApoEII in the murine MPSII model. Subsequently, we have initiated a phase I/II clinical trial in MPSII patients using the IDS.ApoEII vector (ClinicalTrials.gov identifier NCT05665166). This is a first-in-human clinical trial to explore the safety, tolerability, and clinical efficacy of an *ex vivo* gene therapy for MPSII patients.

MATERIALS AND METHODS

Expression vectors

LVs containing the human IDS gene or human IDS gene tagged with ApoEII, under the CD11b promoter, were made as previously described.[12]

LV production and titration

LV was produced as previously described[13] by transient transfection of HEK293T cells with pRSV-Rev, pMDLg/pRRE, pMD2.G (Didier Trono, Addgene plasmids), and LV genome plasmid[13,40–42] and 7.5 mM polyethylenimine (40 kDa; Polysciences[43]). In order to titer the vector, EL4 mouse lymphoma cells (TIB-39; American Type Culture Collection [ATCC], Manassas, VA) were transduced with a dilution series of concentrated LV for 6 h and transduced cells harvested 72 h later. The number of integrated viral genomes per cell, measured using a primer and probe set against HIV-1 ψ sequence, was determined using qPCR using a standard curve generated by dilutions of genomic DNA from an EL4 cell line containing 2 integrated copies/cell of pHRsin.SFFV.eGFP.att.wpre.[40]

Mice and transplantation procedures

Female mice heterozygous for the X-linked allele on a C57BL/6 background were obtained from Prof. Joseph Muenzer (University of North Carolina at Chapel Hill) and bred with WT C57BL/6J males (Envigo, Alconbury, UK). MPSII mice were backcrossed onto the Pep3 CD45.1 congenic background (B6.SJL-Ptprc a Pepc b/BoyJ) to distinguish donor and recipient cells, as previously described.[40] WT littermates were used as controls throughout. Mice were housed in individually ventilated cages with *ad libitum* access to food and water and were kept in a 12 h light/dark cycle. Male mice were used in this study and housed in groups of 2–5 with littermates.

For transplantation studies, total BM mononuclear cells from 6- to 12-week-old male MPSII mice ($CD45.1^+$) were isolated from femurs and tibias and lineage-depleted using the murine lineage cell depletion kit (Miltenyi Biotec, Bisley, UK), as previously described.[13] Cells were stimulated using 100 ng/mL murine stem cell factor, 100 ng/mL murine fms-like tyrosine kinase-3, and 10 ng/mL recombinant murine interleukin-3 (Peprotech, Rocky Hill, NJ) for 3 h prior to

transduction with an LV for 24 h at a multiplicity of infection (MOI) of 100.

Six- to eight-week-old male MPSII mice housed in groups in autoclaved individually ventilated cages were myeloablated using 125 mg/kg busulfan (Busilvex; Pierre Fabre, Boulogne, France) in five daily doses (25 mg/kg/day) via intraperitoneal (i.p.) injection. Within 24 h of myeloablation, mice were injected with 3–4 × 10^5 lineage-depleted transduced HSCs via the lateral tail vein. For WT transplants (WT-HSCT), busulfan-conditioned MPSII mice received 1–2 × 10^7 untransduced total BM cells from 6- to 12-week-old WT donors ($CD45.1^+$). No adverse effects were detected after engraftment. Engraftment of donor HSCs was assessed at 4 weeks post-transplantation in peripheral blood by flow cytometry, as previously described.[12]

Sample processing

For this long-term study, mice were allowed to reach their humane endpoint before being culled and tissues harvested. The humane endpoint, as defined in our home office license, is the point at which mice started showing symptoms of sickness such as >15% weight loss, hair loss, lack of grooming or abnormal appearance, hunched posture, piloerection, lethargy on touching, facial grimace, respiratory distress, or neurological signs such as circling. The majority of treated and untreated MPSII mice demonstrated a gradual decline in health following the 6 month time point and demonstrating one or more of the above symptoms. A few mice in the long-term cohorts, including WT mice, demonstrated circling and were culled immediately and tissues harvested. MPSII controls and treated animals were harvested between 13 and 16 months on average. WT controls were typically harvested at 16–19 months. Six mice per group were anesthetized and transcardially perfused with 37°C PBS. One brain hemisphere was fixed in 4% paraformaldehyde (PFA) for 24 h and transferred to 30% sucrose and 2 mmol/L MgCl2/PBS solution for 48 h before freezing at −80°C. Samples of brain, spleen, heart, and liver were snap-frozen on dry ice. BM samples were collected by flushing one tibia and femur with 1 mL 2% fetal bovine serum (FBS)/PBS, filtered using a 70 μm cell strainer and lysed using red blood cell lysis buffer (150 mM NH_4Cl, 10 mM $KHCO_3$, 0.1 mM EDTA [pH 7.2–7.4]). For enzyme activity assays, samples were homogenized and sonicated in homogenization buffer (0.5 M NaCl, 0.02 M Tris, 0.1% Triton X-100 [pH 7]). Genomic DNA used for organ VCN analysis was extracted using the GenElute Mammalian Genomic DNA Miniprep kit (Sigma-Aldrich).

Enzyme activity assays

IDS enzyme activity was measured in a two-step protocol using the fluorescent substrate MU-αIdoA-2S (Carbosynth) and Aldurazyme (Genzyme), as previously described.[44] Starting material was standardized to 20 μg total protein or plasma; 40 μg for liver, heart, lung, spleen, and BM; and 60 μg for brain using a BCA assay (Thermo Fisher Scientific). For β-hexosaminidase activity, 1 μg total protein from brain or 2 μg from spleen and plasma were added to 0.5 mM 4-methylumbelliferyl-N-acetyl-β-d-glucosaminide substrate (Sigma-Aldrich), incubated for 40 min at 37°C, and stopped with 200 μL of 0.2 M carbonate buffer. Fluorescence was measured using the BioTek Synergy HT plate reader (excitation 360 nm, emission 460 nm).

Immunohistochemistry

Free-floating immunohistochemistry (IHC) was performed on 30 μm PFA-fixed coronal brain sections using rabbit anti-NeuN (1:1,000; ab177487; Abcam), rabbit anti-GFAP (1:1,500; Z0334; Dako, Stockport, UK), and rat anti-LAMP2 (1:500; ab13524; Abcam) primary antibodies using standard protocols.[12] ILB4 (5 μg/mL; L5391; Sigma-Aldrich) was visualized on 30 μm coronal brain sections using DAB substrate for 40 s (Vector, Peterborough, UK) using standard protocols.[13] Images were acquired on a 3DHISTECH Pannoramic-250 microscope slide-scanner using a 20×/0.30 Plan Achromat objective (Zeiss) with extended focus and the DAPI, fluorescein isothiocyanate (FITC), and tetramethyl rhodamine (TRITC) filter sets. Snapshots of the slide-scans were taken using CaseViewer software (3DHISTECH). Nonlinear adjustments were made to all immunofluorescence images equally to eliminate background: gamma 0.72 and input levels of 0–190. GFAP immunofluorescence was quantified using ImageJ software on four sections per mouse for cortex, 2 sections per mouse for striatum and one section per mouse for the hippocampus and amygdala (n = 3–6/group). Counts of ILB4-positive cells were performed on four sections per mouse (n = 3–6/group) at 20× magnification and counted manually using ImageJ software. LAMP2 area quantification was performed using CellProfiler software on 40× images capture using CaseViewer. H&E staining was performed at Histologix using validated GLP protocols.

Glycosaminoglycan analysis

Soluble brain and liver fractions were collected and processed as previously described.[45] HS chains were digested using 0.2 mI/U each of heparinase I, II, and III, and CS/DS chains were digested using 2 mI/U chondroitinase ABC (Sigma-Aldrich) in 50 mM Tris/50 mM NaCl (pH 7.9). Resulting disaccharides were freeze-dried and 2-aminoacridone (AMAC) labeled. HS and CS/DS disaccharides were separated by reverse-phase high-performance liquid chromatography (HPLC) using a Zorbax Eclipse XDB-C18 column (4.6 × 100 mm, 3.5 μm; Agilent), equilibrated in 95% 0.1 M ammonium acetate/5% acetonitrile on an Agilent 1200 Series HPLC system. Disaccharides were eluted over 5%–20% acetonitrile gradient at 0.2 mL/min. AMAC-labeled HS and CS/DS disaccharide standards (Iduron) were used for peak identification. Total fluorescence was compared with known quantities of HS or CS/DS to calculate absolute amounts of each disaccharide. Correction factors were calculated as described.[12]

VCN analysis

To determine the VCN in mice tissues, qDNA was extracted using GenElute Mammalian Genomic DNA Miniprep kit (Sigma-Aldrich) and number of vector integrations determined using qPCR using the WPRE and rodent GAPDH primer probe sets described,[46] and a standard curve was generated by serial dilution of DNA sample

derived from an EL4 cell line clone (ALS EL4 eGFP 2.2) containing two copies of integrated WPRE gene/cell.[13]

Bio-Plex multiplex immunoassay

The expression levels of mouse cytokines, chemokines, and growth factors were quantified using Bio-Plex Pro Mouse Cytokine 23-plex Assay kit (M60009RDPD; Bio-Rad), according to the manufacturer's instructions and read on the Bio-Plex 200 suspension array system using Luminex technology. Tissue samples were homogenized in 100 μL homogenization buffer (0.5 M NaCl, 0.02 M Tris [pH 7]) using a motor pestle in 1.5 mL Eppendorf tubes. The samples were then further processed by adding 100 μL homogenization buffer plus 0.1% Triton X-100 and sonicated on ice for 3 × 5 s at 5 μm amplitude using an MSE Soniprep 150 Plus Ultrasonic Disintegrator. Homogenized samples were then centrifuged at 2000 × g at 4°C for 20 min, and the supernatant was eluted and stored at −80°C until required. Prior to loading, protein concentrations were quantified using BCA assay (Thermo Fisher Scientific) and loaded at an optimized protein concentration of 5,000 μg. The data produced were analyzed using the Bio-Plex software manager.

Study approval

Animal experiments were ethically approved by the Manchester Research Ethics Committee and performed under UK Home Office regulations and PPLs 40/3658 and POC3AEEB0.

Statistics

Statistical analysis was performed using Prism 9 software (GraphPad, La Jolla, CA). One-way or two-way ANOVA was performed for multi-group analysis followed by Tukey's multi-comparisons test. Significance was set at $p < 0.05$. MPSII mutant mice and WT littermates were randomly assigned to control or transplantation groups, although transplantation group allocation was also partially determined by the number of donor animals and the amount of cells available for transplant.

DATA AND CODE AVAILABILITY

Raw data were generated at University of Manchester. Derived data supporting the findings of this study are available from the corresponding author (B.W.B.) on request if authorized by our study sponsor.

SUPPLEMENTAL INFORMATION

Supplemental information can be found online at https://doi.org/10.1016/j.omtm.2023.101127.

ACKNOWLEDGMENTS

The authors thank Shire Plc and Professor Jo Muenzer for facilitating the transfer of the MPSII mouse model, Suzanna Dickson for her assistance setting up the Bio-Plex instrument, the Biological Service Facility at the University of Manchester for maintenance and support of mouse colonies, and Histologix for performing the histology study. This work was funded by the Isaac Foundation, the National MPS Society, the Irish MPS Society, and the MRC via a confidence in concept award and Avrobio.

AUTHOR CONTRIBUTIONS

S.E., A.L., and B.W.B. wrote the manuscript. B.W.B. and H.G. conceived and designed the study. H.G. set up transplantation groups. A.L. and H.G. harvested tissues for processing. A.L., S.E., H.P., L.B., J.R., and S.W. acquired, analyzed, and interpreted data.

DECLARATION OF INTERESTS

B.W. and H.G. hold a patent for the use of the IDS.ApoEII in the treatment of MPSII. B.W.B., H.G., S.E., A.L., and S.W. receive royalty payments from AVROBIO for a license to this treatment, and B.W.B. is the recipient of an unrestricted clinical trial grant from AVROBIO for the treatment of MPSII with LV.IDS.ApoEII.

REFERENCES

1. Martin, R., Beck, M., Eng, C., Giugliani, R., Harmatz, P., Muñoz, V., and Muenzer, J. (2008). Recognition and diagnosis of mucopolysaccharidosis II (Hunter syndrome). Pediatrics *121*, e377–e386. https://doi.org/10.1542/peds.2007-1350.

2. Scarpa, M. (1993). Mucopolysaccharidosis Type II. In GeneReviews(®), M.P. Adam, D.B. Everman, G.M. Mirzaa, R.A. Pagon, S.E. Wallace, L.J.H. Bean, K.W. Gripp, and A. Amemiya, eds. (University of Washington). Seattle Copyright © 1993-2022, University of Washington, Seattle. GeneReviews is a registered trademark of the University of Washington, Seattle. All rights reserved.

3. Ellison, S., Parker, H., and Bigger, B. (2023). Advances in therapies for neurological Lysosomal Storage Disorders. J. Inherit. Metab. Dis. *46*, 874–905. https://doi.org/10.1002/jimd.12615.

4. Khan, S.A., Peracha, H., Ballhausen, D., Wiesbauer, A., Rohrbach, M., Gautschi, M., Mason, R.W., Giugliani, R., Suzuki, Y., Orii, K.E., et al. (2017). Epidemiology of mucopolysaccharidoses. Mol. Genet. Metabol. *121*, 227–240. https://doi.org/10.1016/j.ymgme.2017.05.016.

5. Parini, R., and Deodato, F. (2020). Intravenous Enzyme Replacement Therapy in Mucopolysaccharidoses: Clinical Effectiveness and Limitations. Int. J. Mol. Sci. *21*, 2975. https://doi.org/10.3390/ijms21082975.

6. Whiteman, D.A., and Kimura, A. (2017). Development of idursulfase therapy for mucopolysaccharidosis type II (Hunter syndrome): the past, the present and the future. Drug Des. Dev. Ther. *11*, 2467–2480. https://doi.org/10.2147/dddt.S139601.

7. Wood, S.R., and Bigger, B.W. (2022). Delivering gene therapy for mucopolysaccharide diseases. Front. Mol. Biosci. *9*, 965089. https://doi.org/10.3389/fmolb.2022.965089.

8. Bigger, B.W., Saif, M., and Linthorst, G.E. (2015). The role of antibodies in enzyme treatments and therapeutic strategies. Best Pract. Res. Clin. Endocrinol. Metabol. *29*, 183–194. https://doi.org/10.1016/j.beem.2015.01.006.

9. Hobbs, J.R., Hugh-Jones, K., Barrett, A.J., Byrom, N., Chambers, D., Henry, K., James, D.C., Lucas, C.F., Rogers, T.R., Benson, P.F., et al. (1981). Reversal of clinical features of Hurler's disease and biochemical improvement after treatment by bone-marrow transplantation. Lancet *2*, 709–712. https://doi.org/10.1016/s0140-6736(81)91046-1.

10. Guffon, N., Pettazzoni, M., Pangaud, N., Garin, C., Lina-Granade, G., Plault, C., Mottolese, C., Froissart, R., and Fouilhoux, A. (2021). Long term disease burden post-transplantation: three decades of observations in 25 Hurler patients successfully treated with hematopoietic stem cell transplantation (HSCT). Orphanet J. Rare Dis. *16*, 60. https://doi.org/10.1186/s13023-020-01644-w.

11. Horgan, C., Jones, S.A., Bigger, B.W., and Wynn, R. (2022). Current and Future Treatment of Mucopolysaccharidosis (MPS) Type II: Is Brain-Targeted Stem Cell Gene Therapy the Solution for This Devastating Disorder? Int. J. Mol. Sci. *23*, 4854.

12. Gleitz, H.F., Liao, A.Y., Cook, J.R., Rowlston, S.F., Forte, G.M., D'Souza, Z., O'Leary, C., Holley, R.J., and Bigger, B.W. (2018). Brain-targeted stem cell gene therapy corrects mucopolysaccharidosis type II via multiple mechanisms. EMBO Mol. Med. *10*, e8730. https://doi.org/10.15252/emmm.201708730.

13. Sergijenko, A., Langford-Smith, A., Liao, A.Y., Pickford, C.E., McDermott, J., Nowinski, G., Langford-Smith, K.J., Merry, C.L.R., Jones, S.A., Wraith, J.E., et al. (2013). Myeloid/Microglial driven autologous hematopoietic stem cell gene therapy corrects a neuronopathic lysosomal disease. Mol. Ther. *21*, 1938–1949. https://doi.org/10.1038/mt.2013.141.

14. Motas, S., Haurigot, V., Garcia, M., Marcó, S., Ribera, A., Roca, C., Sánchez, X., Sánchez, V., Molas, M., Bertolin, J., et al. (2016). CNS-directed gene therapy for the treatment of neurologic and somatic mucopolysaccharidosis type II (Hunter syndrome). JCI Insight *1*, e86696. https://doi.org/10.1172/jci.insight.86696.

15. Tomatsu, S., Gutierrez, M.A., Ishimaru, T., Peña, O.M., Montaño, A.M., Maeda, H., Velez-Castrillon, S., Nishioka, T., Fachel, A.A., Cooper, A., et al. (2005). Heparan sulfate levels in mucopolysaccharidoses and mucolipidoses. J. Inherit. Metab. Dis. *28*, 743–757. https://doi.org/10.1007/s10545-005-0069-y.

16. Parker, H., and Bigger, B.W. (2019). The role of innate immunity in mucopolysaccharide diseases. J. Neurochem. *148*, 639–651. https://doi.org/10.1111/jnc.14632.

17. Mandolfo, O., Parker, H., and Bigger, B. (2022). Innate Immunity in Mucopolysaccharide Diseases. Int. J. Mol. Sci. *23*, 1999. https://doi.org/10.3390/ijms23041999.

18. Parker, H., Ellison, S.M., Holley, R.J., O'Leary, C., Liao, A., Asadi, J., Glover, E., Ghosh, A., Jones, S., Wilkinson, F.L., et al. (2020). Haematopoietic stem cell gene therapy with IL-1Ra rescues cognitive loss in mucopolysaccharidosis IIIA. EMBO Mol. Med. *12*, e11185. https://doi.org/10.15252/emmm.201911185.

19. Mandolfo, O., Liao, A., Singh, E., O'Leary, C., Holley, R.J., and Bigger, B.W. (2023). Establishment of the Effectiveness of Early Versus Late Stem Cell Gene Therapy in Mucopolysaccharidosis II for Treating Central Versus Peripheral Disease. Hum. Gene Ther. https://doi.org/10.1089/hum.2023.002.

20. Fumagalli, F., Calbi, V., Natali Sora, M.G., Sessa, M., Baldoli, C., Rancoita, P.M.V., Ciotti, F., Sarzana, M., Fraschini, M., Zambon, A.A., et al. (2022). Lentiviral haematopoietic stem-cell gene therapy for early-onset metachromatic leukodystrophy: long-term results from a non-randomised, open-label, phase 1/2 trial and expanded access. Lancet *399*, 372–383. https://doi.org/10.1016/S0140-6736(21)02017-1.

21. Sessa, M., Lorioli, L., Fumagalli, F., Acquati, S., Redaelli, D., Baldoli, C., Canale, S., Lopez, I.D., Morena, F., Calabria, A., et al. (2016). Lentiviral haemopoietic stem-cell gene therapy in early-onset metachromatic leukodystrophy: an ad-hoc analysis of a non-randomised, open-label, phase 1/2 trial. Lancet *388*, 476–487. https://doi.org/10.1016/S0140-6736(16)30374-9.

22. Smith, M.C., Belur, L.R., Karlen, A.D., Erlanson, O., Podetz-Pedersen, K.M., McKenzie, J., Detellis, J., Gagnidze, K., Parsons, G., Robinson, N., et al. (2022). Phenotypic Correction of Murine Mucopolysaccharidosis Type II by Engraftment of Ex Vivo Lentiviral Vector-Transduced Hematopoietic Stem and Progenitor Cells. Hum. Gene Ther. *33*, 1279–1292. https://doi.org/10.1089/hum.2022.141.

23. Association for the Advancement of Blood & Biotherapies press release (2021). FDA clinical hold of phase 3 CALD trial.

24. Cartier, N., Hacein-Bey-Abina, S., Bartholomae, C.C., Bougnères, P., Schmidt, M., Kalle, C.V., Fischer, A., Cavazzana-Calvo, M., and Aubourg, P. (2012). Lentiviral hematopoietic cell gene therapy for X-linked adrenoleukodystrophy. Methods Enzymol. *507*, 187–198. https://doi.org/10.1016/B978-0-12-386509-0.00010-7.

25. Jones, S., Kinsella, J., Holley, R., Potter, J., Booth, C., Buckland, K., Rust, S., Church, H.J., Brammeier, K.L., Lee, H., et al. (2023). Sustained biochemical engraftment and early clinical outcomes following ex-vivo autologous stem cell gene therapy for mucopolysaccharidosis type IIIA. Mol. Genet. Metabol. *138*, 107171. https://doi.org/10.1016/j.ymgme.2022.107171.

26. Gleitz, H.F.E., Dugourd, A.J.F., Leimkühler, N.B., Snoeren, I.A.M., Fuchs, S.N.R., Menzel, S., Ziegler, S., Kröger, N., Triviai, I., Büsche, G., et al. (2020). Increased CXCL4 expression in hematopoietic cells links inflammation and progression of bone marrow fibrosis in MPN. Blood *136*, 2051–2064. https://doi.org/10.1182/blood.2019004095.

27. Holley, R.J., Ellison, S.M., Fil, D., O'Leary, C., McDermott, J., Senthivel, N., Langford-Smith, A.W.W., Wilkinson, F.L., D'Souza, Z., Parker, H., et al. (2018). Macrophage enzyme and reduced inflammation drive brain correction of mucopolysaccharidosis IIIB by stem cell gene therapy. Brain *141*, 99–116. https://doi.org/10.1093/brain/awx311.

28. Visigalli, I., Delai, S., Politi, L.S., Di Domenico, C., Cerri, F., Mrak, E., D'Isa, R., Ungaro, D., Stok, M., Sanvito, F., et al. (2010). Gene therapy augments the efficacy of hematopoietic cell transplantation and fully corrects mucopolysaccharidosis type I phenotype in the mouse model. Blood *116*, 5130–5139. https://doi.org/10.1182/blood-2010-04-278234.

29. Villegas, V.M., and Murray, T.G. (2021). Alphabet Soup: Clinical Pearls for the Retina Specialist-Ocular Toxicity of Advanced Antineoplastic Agents in Systemic Cancer Care. Ophthalmol. Retina *5*, 1181–1186. https://doi.org/10.1016/j.oret.2021.09.008.

30. Fraunfelder, F.T., and Meyer, S.M. (1983). Ocular toxicity of antineoplastic agents. Ophthalmology *90*, 1–3. https://doi.org/10.1016/s0161-6420(83)34600-5.

31. Matusiewicz, M., Neubauer, K., Bednarz-Misa, I., Gorska, S., and Krzystek-Korpacka, M. (2017). Systemic interleukin-9 in inflammatory bowel disease: Association with mucosal healing in ulcerative colitis. World J. Gastroenterol. *23*, 4039–4046. https://doi.org/10.3748/wjg.v23.i22.4039.

32. Gu, Z.W., Wang, Y.X., and Cao, Z.W. (2017). Neutralization of interleukin-9 ameliorates symptoms of allergic rhinitis by reducing Th2, Th9, and Th17 responses and increasing the Treg response in a murine model. Oncotarget *8*, 14314–14324. https://doi.org/10.18632/oncotarget.15177.

33. Vyas, S.P., and Goswami, R. (2018). A Decade of Th9 Cells: Role of Th9 Cells in Inflammatory Bowel Disease. Front. Immunol. *9*, 1139. https://doi.org/10.3389/fimmu.2018.01139.

34. Rauber, S., Luber, M., Weber, S., Maul, L., Soare, A., Wohlfahrt, T., Lin, N.Y., Dietel, K., Bozec, A., Herrmann, M., et al. (2017). Resolution of inflammation by interleukin-9-producing type 2 innate lymphoid cells. Nat. Med. *23*, 938–944. https://doi.org/10.1038/nm.4373.

35. Meng, H., Niu, R., You, H., Wang, L., Feng, R., Huang, C., and Li, J. (2022). Interleukin-9 attenuates inflammatory response and hepatocyte apoptosis in alcoholic liver injury. Life Sci. *288*, 120180. https://doi.org/10.1016/j.lfs.2021.120180.

36. Zheng, K., Zheng, X., and Yang, W. (2022). The Role of Metabolic Dysfunction in T-Cell Exhaustion During Chronic Viral Infection. Front. Immunol. *13*, 843242. https://doi.org/10.3389/fimmu.2022.843242.

37. Lombardi, A., Villa, S., Castelli, V., Bandera, A., and Gori, A. (2021). T-Cell Exhaustion in Mycobacterium tuberculosis and Nontuberculous Mycobacteria Infection: Pathophysiology and Therapeutic Perspectives. Microorganisms *9*, 2460. https://doi.org/10.3390/microorganisms9122460.

38. Goswami, T.K., Singh, M., Dhawan, M., Mitra, S., Emran, T.B., Rabaan, A.A., Mutair, A.A., Alawi, Z.A., Alhumaid, S., and Dhama, K. (2022). Regulatory T cells (Tregs) and their therapeutic potential against autoimmune disorders - Advances and challenges. Hum. Vaccines Immunother. *18*, 2035117. https://doi.org/10.1080/21645515.2022.2035117.

39. Rajendeeran, A., and Tenbrock, K. (2021). Regulatory T cell function in autoimmune disease. J. Transl. Autoimmun. *4*, 100130. https://doi.org/10.1016/j.jtauto.2021.100130.

40. Langford-Smith, A., Wilkinson, F.L., Langford-Smith, K.J., Holley, R.J., Sergijenko, A., Howe, S.J., Bennett, W.R., Jones, S.A., Wraith, J., Merry, C.L., et al. (2012). Hematopoietic stem cell and gene therapy corrects primary neuropathology and behavior in mucopolysaccharidosis IIIA mice. Mol. Ther. *20*, 1610–1621. https://doi.org/10.1038/mt.2012.82.

41. Biffi, A., Capotondo, A., Fasano, S., del Carro, U., Marchesini, S., Azuma, H., Malaguti, M.C., Amadio, S., Brambilla, R., Grompe, M., et al. (2006). Gene therapy of metachromatic leukodystrophy reverses neurological damage and deficits in mice. J. Clin. Invest. *116*, 3070–3082. https://doi.org/10.1172/JCI28873.

42. Siapati, E.K., Bigger, B.W., Miskin, J., Chipchase, D., Parsley, K.L., Mitrophanous, K., Themis, M., Thrasher, A.J., and Bonnet, D. (2005). Comparison of HIV- and EIAV-based vectors on their efficiency in transducing murine and human hematopoietic repopulating cells. Mol. Ther. *12*, 537–546. https://doi.org/10.1016/j.ymthe.2005.01.022.

43. Kuroda, H., Kutner, R.H., Bazan, N.G., and Reiser, J. (2009). Simplified lentivirus vector production in protein-free media using polyethylenimine-mediated transfection. J. Virol. Methods *157*, 113–121. https://doi.org/10.1016/j.jviromet.2008.11.021.

44. Lu, J.Z., Hui, E.K.W., Boado, R.J., and Pardridge, W.M. (2010). Genetic engineering of a bifunctional IgG fusion protein with iduronate-2-sulfatase. Bioconjugate Chem. *21*, 151–156. https://doi.org/10.1021/bc900382q.

45. Holley, R.J., Deligny, A., Wei, W., Watson, H.A., Niñonuevo, M.R., Dagälv, A., Leary, J.A., Bigger, B.W., Kjellén, L., and Merry, C.L.R. (2011). Mucopolysaccharidosis type I, unique structure of accumulated heparan sulfate and increased N-sulfotransferase activity in mice lacking alpha-l-iduronidase. J. Biol. Chem. *286*, 37515–37524. https://doi.org/10.1074/jbc.M111.287474.

46. Ellison, S.M., Liao, A., Wood, S., Taylor, J., Youshani, A.S., Rowlston, S., Parker, H., Armant, M., Biffi, A., Chan, L., et al. (2019). Pre-clinical Safety and Efficacy of Lentiviral Vector-Mediated Ex Vivo Stem Cell Gene Therapy for the Treatment of Mucopolysaccharidosis IIIA. Mol. Ther. Methods Clin. Dev. *13*, 399–413. https://doi.org/10.1016/j.omtm.2019.04.001.

OMTM, Volume *31*

Supplemental information

Sustained long-term disease correction

in a murine model of MPSII

following stem cell gene therapy

Stuart Ellison, Aiyin Liao, Hélène F.E. Gleitz, Helen Parker, Laura Booth, John Robinson, Shaun Wood, Jessica Taylor, Rebecca Holley, and Brian W. Bigger

Immunohistochemical findings by HistologiX

The eye, heart, kidney, liver, spleen and brain slides from treated and untreated mice underwent the standard HistologiX H&E staining protocol.

All H&E stained slides were checked for quality and repeated if required standards were not met.

Pathologist Review

Note: GLP compliance is not claimed for the Pathologist review.

Examination of the stained slides was conducted by a British (FRCPath) and American (Dipl.ACVP) board-certified Histopathologist at PathCelerate. Full toxicologic evaluation of tissues, including assessment of MPSII-mediated morphological change were recorded based upon morphology, severity, and distribution.

Semi-quantitative manual counting was performed on blood smears, with 100 white blood cells counted to give a differential lineage calculation. Undifferentiated lymphoblast quantities were also recorded.

1) Blood smears

Where evaluable, blood smears from all control and treated groups did not show evidence of test article-(inclusive of busulfan) related abnormality. Rare, scattered, and undifferentiated monocytes with expanded, foamy, cytoplasm, containing granular debris and similar in nature to a morphology consistent with “Alder anomaly”, were noted within the blood smears of animals in the MPSII control group, and, to a lesser degree, amongst animals in the WT-HSCT treatment group (Table S1 and Figure S1). This finding was considered to reflect the intra-cellular storage of GAG/breakdown product, amongst white blood cells of various lineage (Bhuyan et al., 2013).

Table S1: Blood smears – Pathologist’s comments

Group	Study	Mouse ID	Pathologist’s Comments
Wild-type	Medium Term (6 months)	238	NA
		239	NA
		240	NA
		265	NA
		266	NA
		267	NA
		286	Neutrophils (mature): 33; Neutrophils (immature): 8; Lymphocytes (mature): 59
	Long Term (12-16 months)	257	Neutrophils (mature): 25; Neutrophils (immature): 18; Lymphocytes (mature): 57
		261	Neutrophils (mature): 18; Neutrophils (immature): 27; Lymphocytes (mature): 55
		262	Neutrophils (mature): 31; Neutrophils (immature): 13; Lymphocytes (mature): 56
		271	Neutrophils (mature): 22; Neutrophils (immature): 12; Lymphocytes (mature): 66
		313	Neutrophils (mature): 24; Neutrophils (immature): 25; Lymphocytes (mature): 51
		328	Neutrophils (mature): 30; Neutrophils (immature): 15; Lymphocytes (mature): 55
		329	Neutrophils (mature): 31; Neutrophils (immature): 25; Lymphocytes (mature): 44
MPSII	Medium Term (6 months)	270	NA
		278	NA
		327	SI (cell number)
		332	Neutrophils (mature): 33; Neutrophils (immature): 17; Lymphocytes (mature): 23; Undifferentiated Lymphocytes/Monocytes (Alder Anomaly); 27
		333	SI (cell number) (Evidence in scattered cells with Alder Anomaly)
		334	Neutrophils (mature): 24; Neutrophils (immature): 12; Lymphocytes (mature): 21; Undifferentiated Lymphocytes/Monocytes (Alder Anomaly); 43
	Long Term (12-16 months)	256	SI (preservation) (Evidence in scattered cells with Alder Anomaly)
		259	Neutrophils (mature): 40; Neutrophils (immature): 18; Lymphocytes (mature): 36; Undifferentiated Lymphocytes/Monocytes (Alder Anomaly); 6
		260	Neutrophils (mature): 24; Neutrophils (immature): 25; Lymphocytes (mature): 39; Undifferentiated Lymphocytes/Monocytes (Alder Anomaly); 12
		346	Neutrophils (mature): 33; Neutrophils (immature): 17; Lymphocytes (mature): 30; Undifferentiated Lymphocytes/Monocytes (Alder Anomaly); 20
		348	Neutrophils (mature): 35; Neutrophils (immature): 25; Lymphocytes (mature): 28; Undifferentiated Lymphocytes/Monocytes (Alder Anomaly); 12
WT-HSCT	Medium Term (6 months)	285	NA
		287	NA
		317	Neutrophils (mature): 26; Neutrophils (immature): 38; Lymphocytes (mature): 30; Undifferentiated Lymphocytes/Monocytes (Alder Anomaly); 6

Group	Study	Mouse ID	Pathologist's Comments
		320	Neutrophils (mature): 28; Neutrophils (immature): 42; Lymphocytes (mature): 26; Undifferentiated Lymphocytes/Monocytes (Alder Anomaly); 4
		321	Neutrophils (mature): 35; Neutrophils (immature): 20; Lymphocytes (mature): 27; Undifferentiated Lymphocytes/Monocytes (Alder Anomaly); 18
		323	SI (cell number)
	Long Term (12-16 months)	301	Neutrophils (mature): 39; Neutrophils (immature): 27; Lymphocytes (mature): 29; Undifferentiated Lymphocytes/Monocytes (Alder Anomaly); 5
		302	Neutrophils (mature): 27; Neutrophils (immature): 45; Lymphocytes (mature): 22; Undifferentiated Lymphocytes/Monocytes (Alder Anomaly); 6
		309	Neutrophils (mature): 30; Neutrophils (immature): 31; Lymphocytes (mature): 39
		310	SI (preservation) (Evidence in rare cells with Alder Anomaly)
		314	NA
		315	SI (preservation) (Evidence in rare cells with Alder Anomaly)
		316	Neutrophils (mature): 32; Neutrophils (immature): 28; Lymphocytes (mature): 40
		421	Neutrophils (mature): 25; Neutrophils (immature): 41; Lymphocytes (mature): 34
		422	Neutrophils (mature): 24; Neutrophils (immature): 54; Lymphocytes (mature): 19; Undifferentiated Lymphocytes/Monocytes (Alder Anomaly); 3
LV.IDS	Medium Term (6 months)	324	Neutrophils (mature): 47; Neutrophils (immature): 26; Lymphocytes (mature): 27
		325	Neutrophils (mature): 35; Neutrophils (immature): 45; Lymphocytes (mature): 20
		361	NA
		362	NA
		363	NA
		364	NA
	Long Term (12-16 months)	355	NA
		341	Neutrophils (mature): 38; Neutrophils (immature): 18; Lymphocytes (mature): 44
		342	Neutrophils (mature): 20; Neutrophils (immature): 20; Lymphocytes (mature): 60
		350	Neutrophils (mature): 26; Neutrophils (immature): 15; Lymphocytes (mature): 59
		357	SI (cell number)
		386	NA
LV.IDS.ApoEII	Medium Term (6 months)	397	Neutrophils (mature): 27; Neutrophils (immature): 22; Lymphocytes (mature): 51
		399	Neutrophils (mature): 18; Neutrophils (immature): 34; Lymphocytes (mature): 48
		406	Neutrophils (mature): 31; Neutrophils (immature): 38; Lymphocytes (mature): 31
		407	Neutrophils (mature): 28; Neutrophils (immature): 20; Lymphocytes (mature): 52
		408	Neutrophils (mature): 16; Neutrophils (immature): 19; Lymphocytes (mature): 65
		409	Neutrophils (mature): 22; Neutrophils (immature): 20; Lymphocytes (mature): 58
	Long Term (12-16 months)	4060	NA
		424	NA
		425	NA
		426	NA
		452	NA
		454	NA
		465	NA

2) Eye

Where available, eye samples from individual animals in all groups co-administered busulfan showed evidence of cataract formation, ranging from minimal-to-moderate in severity, and characterized by lenticular fiber fragmentation, with sporadic epithelial hypertrophy/hyperplasia, and occasional Morgagnian globule formation. These were considered a secondary and adverse effect arising from the co-administration of busulfan (Fraunfelder & Meyer, 1983).

Table S2: Eye – Pathologist's comments

Group	Study	Mouse ID	Pathologist's Comments
Wild-type	Medium Term (6 months)	238	NA
		239	NA
		240	NA
		265	NA
		266	NA
		267	NA
		286	NA
	Long Term (12-16 months)	257	NA
		261	NA
		262	NA
		271	NA
		313	-
		328	-
		329	-
MPSII	Medium Term (6 months)	270	NA
		278	NA
		327	NA
		332	NA

Group	Study	Mouse ID	Pathologist's Comments
		333	NA
		334	NA
	Long Term (12-16 months)	256	Atrophy, retina (outer nuclear layer); 2 d
		259	Atrophy, retina (outer nuclear layer); 3 d
		260	Atrophy, retina (outer nuclear layer); 3 d
		346	Atrophy, retina (outer nuclear layer); 2 d
		348	Atrophy, retina (outer nuclear layer); 3 d
WT-HSCT	Medium Term (6 months)	285	NA
		287	NA
		317	NA
		320	NA
		321	NA
		323	NA
	Long Term (12-16 months)	301	NA
		302	NA
		309	Atrophy, retina (outer nuclear layer); 2 d. Cataract, lenticular; 3 mf
		310	Atrophy, retina (outer nuclear layer); 2 d. Cataract, lenticular; 1 mf
		314	Atrophy, retina (outer nuclear layer); 3 d. Cataract, lenticular; 1 f
		315	Atrophy, retina (outer nuclear layer); 2 d. Cataract, lenticular; 1 mf
		316	Atrophy, retina (outer nuclear layer); 1 d
		421	NA
		422	NA
LV.IDS	Medium Term (6 months)	324	NA
		325	NA
		361	-
		362	Cataract, lenticular; 1 f
		363	Atrophy, retina (outer nuclear layer); 1 d. Cataract, lenticular; 1 mf
		364	Atrophy, retina (outer nuclear layer); 1 d. Cataract, lenticular; 2 mf
	Long Term (12-16 months)	355	Atrophy, retina (outer nuclear layer); 1 d
		341	NA
		342	NA
		350	Atrophy, retina (outer nuclear layer); 1 d. Cataract, lenticular; 1 mf
		357	NA
		386	Atrophy, retina (outer nuclear layer); 1 d. Cataract, lenticular; 2 mf
LV.IDS.ApoEII	Medium Term (6 months)	397	Cataract, lenticular; 1 mf
		399	Atrophy, retina (outer nuclear layer); 1 d. Cataract, lenticular; 2 mf
		406	Atrophy, retina (outer nuclear layer); 1 d. Cataract, lenticular; 1 mf
		407	Cataract, lenticular; 1 mf
		408	Cataract, lenticular; 1 mf
		409	Cataract, lenticular; 1 mf
	Long Term (12-16 months)	4060	Atrophy, retina (outer nuclear layer); 1 d. Cataract, lenticular; 2 mf
		424	Atrophy, retina (outer nuclear layer); 1 d. Cataract, lenticular; 1 f
		425	Atrophy, retina (outer nuclear layer); 1 d. Cataract, lenticular; 1 mf
		426	Atrophy, retina (outer nuclear layer); 1 d. Cataract, lenticular; 1 mf
		452	Atrophy, retina (outer nuclear layer); 1 d. Cataract, lenticular; 2 mf
		454	Atrophy, retina (outer nuclear layer); 1 d. Cataract, lenticular; 2 mf
		465	Cataract, lenticular; 1 mf

3) Heart

Minimal interstitial aggregates of vacuolated macrophages were observed in animals from the MPSII group, at both time-points, in isolation.

Table S3: Heart - Pathologist's Comments

Group	Study	Mouse ID	Pathologist's Comments
	Medium Term (6 months)	238	-
		239	-
		240	-
		265	NA
		266	NA
		267	NA
		286	NA
	Long Term (12-16 months)	257	-
		261	-
		262	-
		271	-
		313	-
		328	-
		329	-
MPSII		270	NA
		278	NA

Group	Study	Mouse ID	Pathologist's Comments
	Medium Term (6 months)	327	Infiltrate, vacuolated macrophages, interstitium; 1 mf
		332	Infiltrate, vacuolated macrophages, interstitium; 1 mf
		333	NA
		334	Infiltrate, vacuolated macrophages, interstitium; 1 mf
	Long Term (12-16 months)	256	Infiltrate, vacuolated macrophages, interstitium; 1 mf
		259	Infiltrate, vacuolated macrophages, interstitium; 1 mf
		260	Infiltrate, vacuolated macrophages, interstitium; 1 mf
		346	Infiltrate, vacuolated macrophages, interstitium; 1 mf
		348	Infiltrate, vacuolated macrophages, interstitium; 1 mf
WT-HSCT	Medium Term (6 months)	285	-
		287	-
		317	-
		320	-
		321	-
		323	-
	Long Term (12-16 months)	301	-
		302	-
		309	-
		310	-
		314	-
		315	-
		316	-
		421	-
		422	Inflammation, vessel; 1 f
LV.IDS	Medium Term (6 months)	324	-
		325	-
		361	-
		362	-
		363	-
		364	-
	Long Term (12-16 months)	355	-
		341	-
		342	-
		350	-
		357	NA
		386	-
LV.IDS.ApoEII	Medium Term (6 months)	397	-
		399	-
		406	-
		407	-
		408	-
		409	-
	Long Term (12-16 months)	4060	Inflammation, vessel; 1 f
		424	-
		425	-
		426	-
		452	-
		454	-
		465	-

4) Kidney

Amongst MPSII mice, there was a spectrum of findings associated with the intra-cellular accumulation of GAG. This encompassed minimal-to-slight accumulations of vacuolated macrophages/histiocytes within the interstitium, spreading to involve the renal tubular epithelium, and glomeruli. At the 12-16 month time-point, there was accompanying inflammatory cell infiltration around local vascular profiles, indicative of mild inflammation (nephritis). In all treatment groups, and at both time-points, this spectrum of findings was attenuated, with an increased level of efficacy noted amongst animals administered LV.IDS or LV.IDS.ApoEII.

Table S4: Kidney- Pathologist's Comments

Group	Study	Mouse ID	Pathologist's Comments
Wild-type	Medium Term (6 months)	238	-
		239	-
		240	Infiltrate, inflammatory cell; 1 f
		265	Infiltrate, inflammatory cell; 1 f
		266	-
		267	Basophilia, tubule; 1 f
		286	NA
		257	-

Group	Study	Mouse ID	Pathologist's Comments
	Long Term (12-16 months)	261	Basophilia, tubule; 1 f
		262	-
		271	Infiltrate, inflammatory cell; 1 f
		313	-
		328	-
		329	-
MPSII	Medium Term (6 months)	270	Vacuolation, glomerular; 1 mf. Vacuolation, tubular; 2 mf. Infiltrate, vacuolated macrophages, interstitium; 1 mf
		278	Infiltrate, vacuolated macrophages, interstitium; 1 mf. Infiltrate, inflammatory cell; 1 f
		327	Vacuolation, glomerular; 1 mf. Vacuolation, tubular; 2 mf. Infiltrate, vacuolated macrophages, interstitium; 1 mf
		332	Vacuolation, glomerular; 1 mf. Vacuolation, tubular; 2 mf. Infiltrate, vacuolated macrophages, interstitium; 1 mf
		333	Vacuolation, glomerular; 1 mf. Infiltrate, vacuolated macrophages, interstitium; 1 mf
		334	Vacuolation, glomerular; 1 mf. Vacuolation, tubular; 1 mf. Infiltrate, vacuolated macrophages, interstitium; 1 mf. Infiltrate, inflammatory cell; 1 f
	Long Term (12-16 months)	256	Vacuolation, glomerular; 1 mf. Vacuolation, tubular; 2 mf. Infiltrate, vacuolated macrophages, interstitium; 2 mf. Infiltrate, inflammatory cell, perivascular; 1 mf
		259	Vacuolation, glomerular; 1 mf. Vacuolation, tubular; 1 mf. Infiltrate, vacuolated macrophages, interstitium; 1 mf. Infiltrate, inflammatory cell, perivascular; 1 mf
		260	Vacuolation, glomerular; 1 mf. Vacuolation, tubular; 1 mf. Infiltrate, vacuolated macrophages, interstitium; 2 mf. Infiltrate, inflammatory cell, perivascular; 1 f
		346	Vacuolation, glomerular; 1 mf. Vacuolation, tubular; 1 mf. Infiltrate, vacuolated macrophages, interstitium; 1 mf. Infiltrate, inflammatory cell, perivascular; 1 f
		348	NA
WT-HSCT	Medium Term (6 months)	285	Vacuolation, tubular; 1 mf
		287	-
		317	Vacuolation, glomerular; 1 mf. Vacuolation, tubular; 1 mf
		320	Vacuolation, glomerular; 1 mf. Vacuolation, tubular; 1 mf. Infiltrate, inflammatory cell; 1 f
		321	Vacuolation, tubular; 1 mf. Infiltrate, inflammatory cell; 1 f
		323	Vacuolation, tubular; 1 mf. Infiltrate, inflammatory cell; 1 f
	Long Term (12-16 months)	301	Vacuolation, glomerular; 1 mf. Vacuolation, tubular; 1 mf. Infiltrate, inflammatory cell; 1 f
		302	Vacuolation, tubular; 1 mf. Infiltrate, inflammatory cell; 1 f
		309	Vacuolation, tubular; 1 mf. Infiltrate, inflammatory cell; 1m f
		310	Vacuolation, tubular; 1 mf. Infiltrate, inflammatory cell; 1 f
		314	Vacuolation, tubular; 1 mf. Infiltrate, inflammatory cell; 1 f
		315	Vacuolation, glomerular; 1 mf. Vacuolation, tubular; 1 mf. Basophilia, tubule; 1 f
		316	Vacuolation, tubular; 1 mf
		421	Vacuolation, tubular; 1 mf. Infiltrate, inflammatory cell; 1 f
		422	Vacuolation, glomerular; 1 mf. Vacuolation, tubular; 2 mf. Infiltrate, inflammatory cell; 1 mf
LV.IDS	Medium Term (6 months)	324	Infiltrate, inflammatory cell; 1 f
		325	Vacuolation, tubular; 1 mf. Infiltrate, inflammatory cell; 1 mf
		361	Vacuolation, tubular; 1 mf. Infiltrate, inflammatory cell; 1 mf
		362	Vacuolation, tubular; 1 mf
		363	Vacuolation, tubular; 1 mf. Infiltrate, inflammatory cell; 1 mf
		364	Infiltrate, inflammatory cell; 1 f
	Long Term (12-16 months)	355	Infiltrate, inflammatory cell; 1 mf
		341	Infiltrate, inflammatory cell; 1 f
		342	Vacuolation, tubular; 1 mf. Infiltrate, inflammatory cell; 1 mf
		350	Vacuolation, glomerular; 1 mf. Vacuolation, tubular; 1 mf. Infiltrate, inflammatory cell; 1 mf. Basophilia, tubule; 1 f
		357	NA
		386	Vacuolation, tubular; 1 mf. Basophilia, tubule; 1 f
LV.IDS.ApoEII	Medium Term (6 months)	397	Vacuolation, tubular; 1 mf
		399	Vacuolation, tubular; 1 mf. Infiltrate, inflammatory cell; 1 mf
		406	Vacuolation, tubular; 1 mf. Infiltrate, inflammatory cell; 1 mf
		407	Infiltrate, inflammatory cell; 1 mf
		408	Vacuolation, tubular; 1 mf
		409	Vacuolation, tubular; 1 mf. Infiltrate, inflammatory cell; 1 mf
	Long Term (12-16 months)	4060	Infiltrate, inflammatory cell; 1 mf
		424	Infiltrate, inflammatory cell; 1 f
		425	Vacuolation, tubular; 1 mf. Infiltrate, inflammatory cell; 1 mf
		426	Infiltrate, inflammatory cell; 1 mf
		452	Infiltrate, inflammatory cell; 1 f
		454	Vacuolation, tubular; 1 mf. Infiltrate, inflammatory cell; 1 mf
		465	Vacuolation, tubular; 1 mf

5) Liver

Amongst MPSII mice, there were a spectrum of findings associated with the intra-cellular accumulation of GAG. This encompassed minimal-to-slight accumulations of vacuolated macrophages/histiocytes within the interstitium, spreading to involve hepatocytes, at minimal-to-moderate severity. At the 12-16 month time-point, there was accompanying minimal-to-slight hepatocellular necrosis. In all treatment groups, and at both time-points, this spectrum of findings was attenuated, with findings of minimal-to-slight hepatocellular vacuolation, in isolation.

Table S5: Liver- Pathologist's Comments

Group	Study	Mouse ID	Pathologist's Comments
Wild-type	Medium Term (6 months)	238	Infiltrate, inflammatory cell; 1 f
		239	-
		240	Infiltrate, inflammatory cell; 1 f
		265	-
		266	-
		267	-
		286	NA
	Long Term (10-12 months)	257	Accumulation, fat, hepatocyte; 1 d. Infiltrate, inflammatory cell; 1 f
		261	Accumulation, fat, hepatocyte; 2 d. Infiltrate, inflammatory cell; 1 mf
		262	Accumulation, fat, hepatocyte; 1 mf
		271	Accumulation, fat, hepatocyte; 1 mf. Infiltrate, inflammatory cell; 1 f
		313	Accumulation, fat, hepatocyte; 2 d. Infiltrate, inflammatory cell; 1 mf
		328	Accumulation, fat, hepatocyte; 2 d
		329	Accumulation, fat, hepatocyte; 2 mf
MPSII	Medium Term (6 months)	270	Vacuolation; hepatocyte; 1 d. Infiltrate, vacuolated macrophages, interstitium; 1 mf
		278	Vacuolation; hepatocyte; 1 mf. Infiltrate, vacuolated macrophages, interstitium; 1 mf
		327	Vacuolation; hepatocyte; 2 d. Infiltrate, vacuolated macrophages, interstitium; 1 mf
		332	Vacuolation; hepatocyte; 2 d. Infiltrate, vacuolated macrophages, interstitium; 1 mf
		333	Infiltrate, vacuolated macrophages, interstitium; 1 mf
		334	Vacuolation; hepatocyte; 1 d. Infiltrate, vacuolated macrophages, interstitium; 1 mf
	Long Term (10-12 months)	256	Vacuolation; hepatocyte; 3 d. Infiltrate, vacuolated macrophages, interstitium; 2 mf. Necrosis, hepatocyte; 1 mf
		259	Vacuolation; hepatocyte; 1 d. Infiltrate, vacuolated macrophages, interstitium; 1 mf. Necrosis, hepatocyte; 1 mf
		260	Vacuolation; hepatocyte; 1 mf. Infiltrate, vacuolated macrophages, interstitium; 2 mf. Necrosis, hepatocyte; 2 mf
		346	Vacuolation; hepatocyte; 2 d. Infiltrate, vacuolated macrophages, interstitium; 2 mf. Necrosis, hepatocyte; 1 f
		348	Vacuolation; hepatocyte; 1 mf. Infiltrate, vacuolated macrophages, interstitium; 2 mf. Necrosis, hepatocyte; 1 f
WT-HSCT	Medium Term (6 months)	285	Infiltrate, inflammatory cell; 1 f
		287	-
		317	Vacuolation; hepatocyte; 1 d
		320	Vacuolation; hepatocyte; 1 d. Infiltrate, inflammatory cell; 1 f
		321	Infiltrate, inflammatory cell; 1 f
		323	Vacuolation; hepatocyte; 1 d. Infiltrate, inflammatory cell; 1 f
	Long Term (10-12 months)	301	Infiltrate, inflammatory cell; 1 f
		302	Infiltrate, inflammatory cell; 1mf
		309	Vacuolation; hepatocyte; 1 d.
		310	Vacuolation; hepatocyte; 1 mf. Infiltrate, inflammatory cell; 1mf
		314	Vacuolation; hepatocyte; 1 d
		315	Vacuolation; hepatocyte; 2 d
		316	Vacuolation; hepatocyte; 1 d
		421	Vacuolation, hepatocyte; 1 d. Infiltrate, inflammatory cell; 1 f
		422	Vacuolation, hepatocyte; 1 mf. Infiltrate, inflammatory cell; 1 mf
LV.IDS	Medium Term (6 months)	324	-
		325	Vacuolation; hepatocyte; 1 d
		361	Vacuolation; hepatocyte; 1 d
		362	Vacuolation; hepatocyte; 1 d
		363	Vacuolation; hepatocyte; 1 d
		364	-
	Long Term (10-12 months)	355	Vacuolation; hepatocyte; 1 d
		341	Vacuolation; hepatocyte; 1 d
		342	Vacuolation; hepatocyte; 1 d. Infiltrate, inflammatory cell; 1 f
		350	Vacuolation; hepatocyte; 1 d. Infiltrate, inflammatory cell; 1 f
		357	NA
		386	Vacuolation; hepatocyte; 1 d
LV.IDS.ApoEII	Medium Term (6 months)	397	Vacuolation; hepatocyte; 1 d
		399	Vacuolation; hepatocyte; 1 d
		406	Vacuolation; hepatocyte; 1 d
		407	Infiltrate, inflammatory cell; 1 mf
		408	Infiltrate, inflammatory cell; 1 f

Group	Study	Mouse ID	Pathologist's Comments
		409	Infiltrate, inflammatory cell; 1 f
	Long Term (10-12 months)	4060	Vacuolation; hepatocyte; 1 d
		424	Vacuolation; hepatocyte; 2 d. Infiltrate, inflammatory cell; 1 mf
		425	Vacuolation; hepatocyte; 1 d
		426	Vacuolation; hepatocyte; 1 d
		452	Vacuolation; hepatocyte; 1 d
		454	Vacuolation; hepatocyte; 1 d. Infiltrate, inflammatory cell; 1 mf
		465	Vacuolation; hepatocyte; 2 d. Infiltrate, inflammatory cell; 1 mf

6) Spleen

Morphologic findings within the spleen were restricted to minimal findings of vacuolated macrophages/histiocytes within the red pulp, extending to the white pulp at up to slight severity, at the 12-16 month time-point, amongst MPSII mice only.

Table S6: Spleen- Pathologist's Comments

Group	Study	Mouse ID	Pathologist's Comments
Wild-type	Medium Term (6 months)	238	-
		239	Increased cellularity, germinal centers; 1 mf
		240	Increased cellularity, germinal centers; 2 mf
		265	-
		266	Increased cellularity, germinal centers; 1 mf
		267	Increased cellularity, germinal centers; 1 mf
		286	NA
	Long Term (10-12 months)	257	-
		261	-
		262	-
		271	-
		313	-
		328	-
		329	-
MPSII	Medium Term (6 months)	270	Infiltrate, vacuolated macrophages, red pulp; 1 mf
		278	Infiltrate, vacuolated macrophages, red pulp; 1 mf
		327	Infiltrate, vacuolated macrophages, red pulp; 1 mf. Increased cellularity, germinal centers; 1 mf
		332	Infiltrate, vacuolated macrophages, red pulp; 1 mf. Increased cellularity, germinal centers; 1 mf
		333	Infiltrate, vacuolated macrophages, red pulp; 1 mf
		334	Infiltrate, vacuolated macrophages, red pulp; 1 mf
	Long Term (10-12 months)	256	Infiltrate, vacuolated macrophages, red/white pulp; 2 mf
		259	Infiltrate, vacuolated macrophages, red/white pulp; 2 mf
		260	Infiltrate, vacuolated macrophages, red/white pulp; 2 mf
		346	Infiltrate, vacuolated macrophages, red/white pulp; 2 mf
		348	Infiltrate, vacuolated macrophages, red pulp; 1 mf
WT-HSCT	Medium Term (6 months)	285	Increased cellularity, germinal centers; 1 mf
		287	Increased cellularity, germinal centers; 1 mf
		317	-
		320	Increased cellularity, germinal centers; 1 mf
		321	-
		323	-
	Long Term (10-12 months)	301	-
		302	-
		309	-
		310	-
		314	-
		315	-
		316	-
		421	-
		422	-
LV.IDS	Medium Term (6 months)	324	NA
		325	NA
		361	Increased cellularity, germinal centers; 1 mf
		362	Increased cellularity, germinal centers; 1 mf
		363	Increased cellularity, germinal centers; 1 mf
		364	-
	Long Term (10-12 months)	355	-
		341	-
		342	-
		350	-
		357	NA
		386	NA
LV.IDS.ApoEII		397	-

Group	Study	Mouse ID	Pathologist's Comments
	Medium Term (6 months)	399	Increased cellularity, germinal centers; 1 mf
		406	-
		407	Increased cellularity, germinal centers; 1 mf
		408	-
		409	-
	Long Term (10-12 months)	4060	-
		424	-
		425	-
		426	-
		452	-
		454	-
		465	-

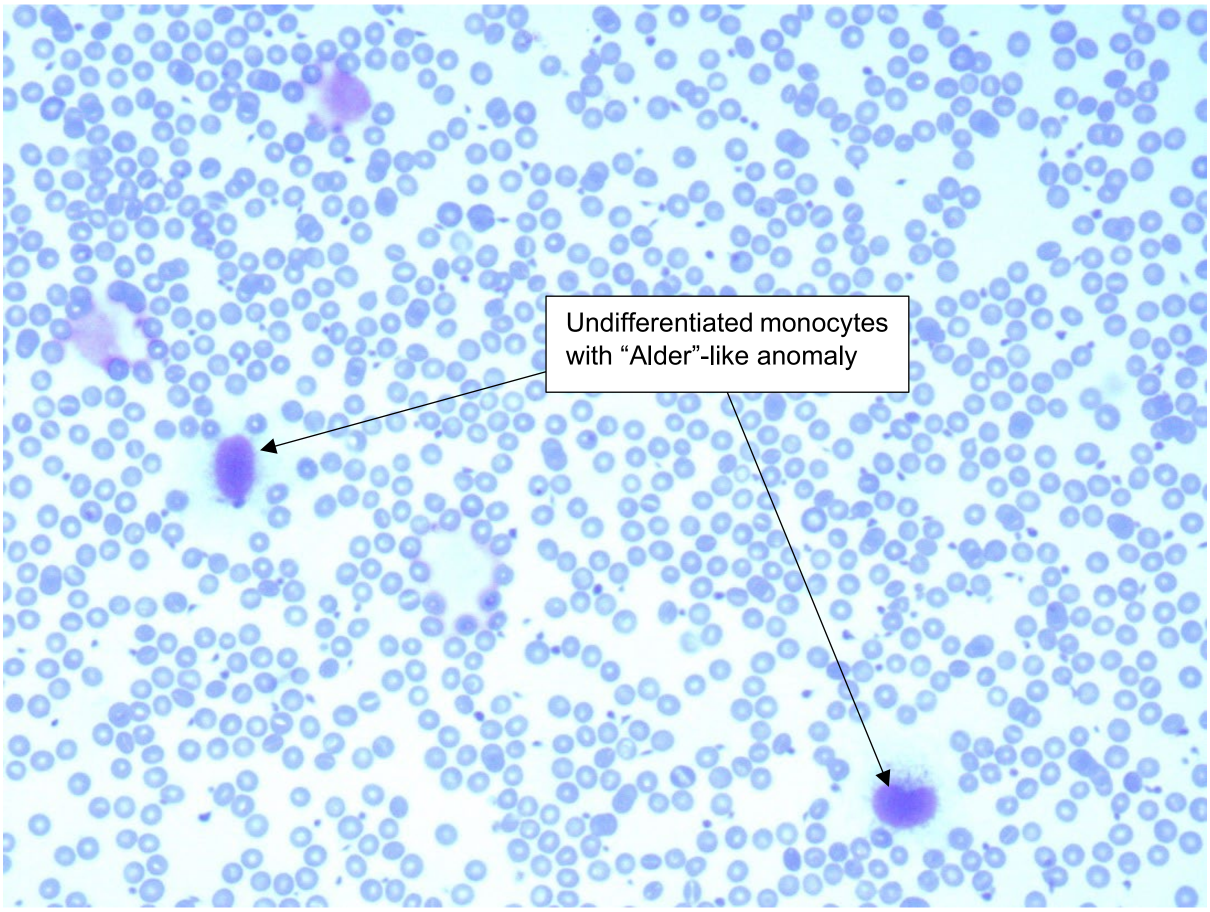

Figure S1: Specimen 334 (MPSII Group, 6 months); blood smear (40x Objective).
